# An Ethanol Extract of *Coptidis rhizoma* Induces Apoptotic Cell Death in Induced Pluripotent Stem Cells and Suppresses Teratoma Formation

**DOI:** 10.3390/nu15102364

**Published:** 2023-05-18

**Authors:** Aeyung Kim, Su-Jin Baek, Sarah Shin, Seo-Young Lee, Sun-Ku Chung

**Affiliations:** 1 Korean Medicine (KM) Application Center, Korea Institute of Oriental Medicine, Daegu 41062, Republic of Korea; 2 KM Data Division, Korea Institute of Oriental Medicine, Daejeon 34054, Republic of Korea; baeksj@kiom.re.kr; 3 KM Science Research Division, Korea Institute of Oriental Medicine, Daejeon 34054, Republic of Korea; s.sarah@kiom.re.kr (S.S.); 09seoyoung03@kiom.re.kr (S.-Y.L.); skchung@kiom.re.kr (S.-K.C.)

**Keywords:** *Coptidis rhizoma*, induced pluripotent stem cells, apoptosis, teratoma, genotoxicity

## Abstract

In cell-based regenerative medicine, induced pluripotent stem cells (iPSCs) generated from reprogrammed adult somatic cells have emerged as a useful cell source due to the lack of ethical concerns and the low risk of immune rejection. To address the risk of teratoma formation, which is a safety issue in iPSC-based cell therapy, it is essential to selectively remove undifferentiated iPSCs remaining in the iPSC-derived differentiated cell product prior to in vivo transplantation. In this study, we explored whether an ethanol extract of coptidis rhizoma (ECR) exhibited anti-teratoma activity and identified the active components involved in the selective elimination of undifferentiated iPSCs. Transcriptome analysis of iPSCs confirmed that cell death-related pathways were significantly altered by ECR treatment. Our results demonstrate that ECR effectively induced apoptotic cell death and DNA damage in iPSCs, and that reactive oxygen species generation, mitochondrial damage, caspase activation, and p53 activation were involved in ECR-mediated iPSC death. However, in iPSC-derived differentiated cells (iPSC-Diff), reduced cell viability and the DNA damage response were not observed after ECR treatment. We co-cultured iPSCs and iPSC-Diff and found that ECR treatment selectively removed iPSCs, whereas iPSC-Diff remained intact. Prior to in ovo implantation, ECR treatment of a mixed cell culture of iPSCs and iPSC-Diff significantly suppressed iPSC-derived teratoma formation. Among the main components of the ECR, berberine and coptisine showed selective cytotoxicity to iPSCs but not to iPSC-Diff. Together, these results indicate the usefulness of ECRs in preparing safe and effective iPSC-based therapeutic cell products with no risk of teratoma formation.

## 1. Introduction

Human induced pluripotent stem cells (iPSCs) and embryonic stem cells (ESCs) have the potential to proliferate indefinitely in vitro and to differentiate into various cell types; therefore, they are useful cell sources for regenerative medicine, drug screening, disease modeling, and toxicity prediction [1,2]. Unlike ESCs, which are developed by manipulating pre-implantation-stage embryos, iPSCs are produced from adult somatic cells such as blood or skin cells by introducing different combinations of reprogramming factors, cellular factors, proteins, peptides, miRNAs, or chemicals [3,4]. Because iPSCs are not associated with ethical questions associated with embryo use and can be customized for patients to avoid immune rejection, they are attracting attention in cell-based regenerative medicine [4,5]. However, following the in vitro differentiation of iPSCs, pluripotent undifferentiated iPSCs may remain in the final cell product, and after transplantation, they may form teratomas, which are considered to be risk factors for iPSC-based cell therapy in the clinical setting [6]. Therefore, to develop safe and effective iPSC-based cell therapies, it is essential to eliminate undifferentiated iPSCs from the final cell product without affecting the functional properties and viability of differentiated cells.

Various strategies have been proposed to selectively remove undifferentiated iPSCs, including treatment with cytotoxic antibodies, small molecules, chemical inhibitors, and the insertion of a miRNA or suicide gene into iPSCs [7,8,9]. However, these methods do not completely address concerns related to specificity, genotoxicity, efficacy, and cost, which are obstacles to clinical application. Recently, we reported that herbal extracts (e.g., *Magnolia cortex* and *Prunellae Spica*) and natural products (e.g., bee venom) suppress teratoma formation through the selective elimination of pluripotent iPSCs and induce low genotoxicity in iPSC-derived differentiated cells (iPSC-Diff) and normal cells [10,11,12]. These herbal extracts are safe for clinical use and low in cost, and are therefore attractive as potential anti-teratoma agents.

*Coptidis rhizoma* (CR), or *huanglian* in Chinese, is the dried root of *Coptis chinensis* Franch, *Coptis teeta* Wall, *Coptis deltoidea* C. Y. Cheng et Hsiao, or *Coptis japonica* Makino [13]. Due to its heat-clearing, damp-drying, fire-purging, and detoxification properties, CR has long been used to treat inflammation and stomach and liver ailments in traditional Chinese medicine in many Asian countries [13]. Recent studies have demonstrated that CR extracts exhibit broad pharmacological activities, including anti-microbial, anti-tumor, anti-inflammatory, anti-hepatic steatosis, anti-hypertension, anti-diabetic, anti-oxidant, and neuroprotective activities [14,15,16]. To date, more than 120 chemical components have been isolated and identified from CR, including alkaloids, lignans, flavonoids, organic acids, phenylpropanoids, and quinones [17]. Chemical studies have revealed that alkaloids, including berberine, coptisine, palmatine, and jatrorrhizine, which are abundant in CR, are its main active ingredients. However, studies of the effects of CR and its main components on iPSCs and teratoma formation have not been conducted.

In this study, we investigated the mechanism of action of a CR ethanol extract (ECR) on iPSCs through transcriptomic analyses and examined whether the ECR was selectively toxic to iPSCs in a mixed cell culture with iPSC-Diff, and consequently suppressed teratoma formation.

## 2. Materials and Methods

### 2.1. Preparation of ECR

We purchased lyophilized powder of ethanol extract of ECR from KOC Biotech (Cat. No. KOC201608-028, Daejeon, Korea). ECR powder was dissolved in 10% dimethyl sulfoxide (DMSO, Sigma Chemical Co., St. Louis, MO, USA) at 50 mg/mL, filtered through a 0.22-μm disk filter, and then stored at −20 °C.

### 2.2. Cells and Reagents

iPSCs were generated from human foreskin fibroblasts and cultured in mTeSR1 medium (Stem Cell Technologies, Vancouver, BC, Canada) on STO feeder cells (CRL-1503; ATCC, Manassas, VA, USA) for maintenance or Matrigel Matrix (#354277; Corning, Bedford, MA, USA) for experiments, as previously reported [18]. iPSC-Diff were generated and maintained as previously reported [10]. z-VAD-fmk and N-acetyl-L-cysteine (NAC) were purchased from Calbiochem (San Diego, CA, USA). Acridine orange (AO), ethidium bromide (EB), 4′,6-diamidino-2-phenylindole (DAPI), doxorubicin, pifithrin-ɑ (PFT-ɑ), coptisine chloride, palmatine chloride, and berberine chloride were obtained from Sigma-Aldrich (St. Louis, MO, USA).

### 2.3. RNA Isolation, Library Preparation, and Whole Transcriptome Profiling Using High-Throughput Sequencing

Total RNAs were isolated from iPSCs treated with 5 μg/mL and 10 μg/mL ECR using the RNeasy Mini Kit (Qiagen, Valencia, CA, USA), and RNA quality was assessed by 2100 Bioanalyzer Instrument (Agilent, Santa Clara, CA, USA). The library was prepared using the MGIEasy RNA Directional Library Prep Kit and sequenced using MGISEQ-2000 (MGI Tech, Shenzhen, China) to generate 100-bp paired-end reads. Reads were trimmed using Trim Galore to remove adapter sequences and reads with low sequence quality. High-quality sequence reads were mapped to the human genome (hg38), and the expression levels of mRNAs were quantified using the DESeq2 [19]. The differences in expression levels between ECR treatment and control groups, in terms of the rate of change (log transformation) and statistical significance (false discovery rate; FDR < 0.01), were analyzed using the edgeR package [20] in R. ClueGO [21], a Cytoscape plug-in tool, was used to functionally grouped gene ontology (GO) and pathway annotation networks.

### 2.4. Cell Viability Assay in Monolayer and Spheroid Culture

To examine cytotoxic effects in monolayer culture, cells including iPSCs and iPSC-Diff were seeded on 12-well culture plates, allowed to adhere, and then treated with indicated concentrations of ECR, doxorubicin, coptisine, palmatine, and berberine for 24 h. Cells were washed with phosphate-buffered saline (PBS) and stained with 0.5 mL crystal violet solution (0.2% crystal violet in 20% methanol) for 30 min at room temperature (RT). After washing with distilled water, stained cells were dissolved with 0.5 mL 1% sodium dodecyl sulfate (SDS) solution, and then the absorbance at 590 nm was measured using a SpectraMax3 microplate reader (Molecular Devices, Sunnyvale, CA, USA). For spheroid culture, cells were seeded on 96-well ultra-low attachment round-bottomed plates, centrifuged at 200× *g* for 3 min, and then incubated at 37 °C in CO_2_ incubator. ECR was added at the time of seeding (co-treatment) or after spheroid formation (post-treatment), and spheroid size was observed under an Olympus IX71 inverted fluorescent microscope (Olympus Optical Co., Ltd., Tokyo, Japan).

### 2.5. Detection of Apoptotic Cell Death by AO/EB and DAPI Staining

To examine the status of cell death, we used AO/EB mixture (each 100 μg/mL in PBS). iPSCs and iPSC-Diff cultured on 12-well culture plates were treated with ECR or doxorubicin for 24 h and then treated AO/EB mixture for 20 min at RT. After washing with mTeSR1 medium, cells were observed under an Olympus IX71 inverted fluorescent microscope. Viable, early apoptotic, late apoptotic, and necrotic cells were identified as green, condensed green, yellow to orange, and red, respectively. To detect apoptotic nuclei, cells grown on the confocal dishes were treated with ECR or doxorubicin for 24 h, fixed with 10% formalin for 30 min at RT, stained with DAPI solution (1 μg/mL) for 10 min at RT, and then observed under an Olympus IX71 inverted fluorescent microscope.

### 2.6. Immunofluorescence Analysis for the γ-H2AX Foci

iPSCs and iPSC-Diff cultured on the confocal dishes were treated with ECR for 24 h or doxorubicin for 6 h. Cells were washed with cold PBS three times, fixed with 10% formalin for 30 min at RT, permeabilized with 0.1% Triton X-100 in PBS for 30 min at RT, and blocked with 3% bovine serum albumin (BSA) in PBS for 1 h at RT. Cells were incubated with anti-p-H2AX antibody (1:1000 dilution, #2577, Cell Signaling Technology, Danvers, MA, USA) overnight at 4 °C, followed by staining with Alexa Fluor 594 anti-rabbit IgG antibody (1:1000 dilution, Thermo Scientific, Rockford, IL, USA) for 3 h at RT. After counter-staining nuclei with DAPI solution, γ-H2AX foci were observed under an Olympus IX71 inverted fluorescent microscope.

### 2.7. Measurement of Intracellular Reactive Oxygen Species (ROS)

iPSCs were grown on Matrigel-coated 96-well black/clear bottom plates (Thermo Scientific), treated with ECR for 6 h, and then measured intracellular ROS generation using ROS-ID Total ROS detection kit (Enzo Life Sciences, Farmingdale, NY, USA) according to the manufacturer’s protocol. After adding oxidative stress detection reagent into ECR-treated iPSCs, fluorescence intensities were measured at Ex/Em = 490/525 nm using a SpectraMax3 microplate reader.

### 2.8. Detection of Mitochondrial Membrane Potential (MMP)

iPSCs grown on confocal dishes were treated with ECR or doxorubicin for 12 h or 3 h, respectively. After washing with mTeSR1 medium, cells were incubated with JC-1 (5 μg/mL, Invitrogen/Molecular probes) in the dark for 10 min at 37 °C. Cells were washed with mTeSR1 medium and then observed under an Olympus IX71 inverted fluorescent microscope.

### 2.9. Western Blot Analysis

Whole-cell lysates were obtained using M-PER mammalian protein extraction reagent (Thermo Scientific), and the protein concentrations were determined using a bicinchoninic acid kit (Sigma-Aldrich). Cell lysates were separated by SDS-PAGE and immunoblotted using specific antibodies as described previously [12]. Antibodies against p-ATM (#5883), p-H2AX (#2577), p53 (#48818), p-p53 (#9284), cleaved caspase-3 (#9664), cleaved caspase-9 (#9505), PARP (#9542), and HRP-conjugated anti-mouse IgG (#7076) and anti-rabbit IgG (#7074) were obtained from Cell Signaling Technologies. Anti-β-actin (sc-47778) was obtained from Santa Cruz Biotechnology Inc. (Santa Cruz, CA, USA). Band intensities of representative immunoblots from two or more experiments were quantified using ImageJ software. Relative values of target proteins were obtained after normalization to the level of β-actin.

### 2.10. Assessment of Caspase Activity

The activities of caspase-3 and -9 in ECR-treated iPSCs were determined using caspase colorimetric assay kit (#K106 and #K119). In brief, iPSCs were treated with ECR for 24 h and then lysed with cell lysis buffer. Cell lysates (50 μg protein per 50 μL cell lysis buffer) were mixed with 50 μL 2× reaction buffer, and then 5 μL of caspase-3 substrate (DEVD-pNA) and caspase-9 substrate (LEHD-pNA) were added into the mixture. After incubation for 1 h at 37 °C, the absorbance was measured at 405 nm using the SpectraMax3 microplate reader.

### 2.11. Analysis of Selective Elimination of iPSC in the Mixed Population with iPSC-Diff

To co-culture iPSCs and iPSC-Diff, iPSCs were first seeded on Matrigel matrix-coated 12-well culture plates with mTeSR1 medium. After 24 h, iPSC-Diff labeled with CellTracker Green 5-chloromethylfluorescein diacetate (CMFDA) dye (Invitrogen, Waltham, MA, USA) were added to iPSCs and co-cultured in the presence or absence of ECR for 24 h. Cells were observed under an Olympus IX71 inverted fluorescent microscope and analyzed for green fluorescence by flow cytometry using an LSRFortessa X-20 (BD Biosciences, San Jose, CA, USA).

### 2.12. In Ovo Teratoma Formation Assay

Fertilized chicken eggs purchased from Pulmuone Co., Ltd. (Seoul, Korea) were incubated in an egg incubator (MX-190 CD; R-COM, Gimhae, Korea) at 37 °C with 65% humidity. The start day of incubation was set as embryonic development (ED) day 0. On ED day 4, albumin was removed using a syringe, and windows were made at the blunt end of eggs. After sealing windows with adhesive tape, eggs were further incubated in an egg incubator. On ED day 9, iPSCs and iPSC-Diff co-cultured at the ratio of 1:1 were treated with 10 μg/mL ECR for 24 h. On ED day 10, cells were all harvested, mixed with 50 μL cold Matrigel, solidified at 37 °C for 30 min, and then loaded on the chorioallantoic membrane (CAM) of eggs. After further incubating for 8 days, teratomas on CAMs were excised, photographed, and weighed.

### 2.13. Ultra High-Performance Liquid Chromatography (UHPLC) Analysis

For chromatographic analysis of ECR was carried out using an Agilent 1290 infinity UHPLC-DAD system (Waldbronn, Germany). ECR, coptisine, palmatine, and berberine were dissolved in methanol at 2 mg/mL, and separation was performed using a Phenomenex Luna C18 column. The detailed UHPLC analysis condition is shown in Table 1. In a quantitative analysis, calibration curves of three standard compounds showed good linearity with *r*^2^ = 0.999 in optimized concentration ranges (Table 2).

### 2.14. Statistical Analysis

Statistical significance was determined using GraphPad Prism 5 Software (GraphPad, San Diego, CA, USA). Two-tailed Student’s *t*-test and one-way analysis of variance (ANOVA) were used for comparison between two groups and three or more groups, respectively. A value of *p* < 0.05 was considered statistically significant.

## 3. Results and Discussion

### 3.1. Identification of Functional Clusters of ECR-Treated iPSCs

**Figure 1:**
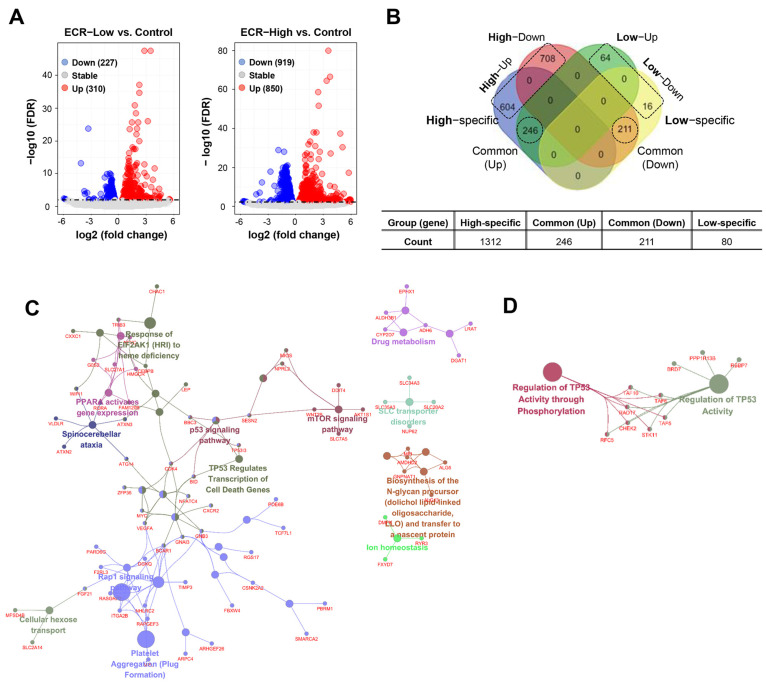
Expression profiling of ECR-treated iPSCs. (**A**) Volcano plot to visualize the DEGs between the ECR-treated iPSC (5 μg/mL (low) or 10 μg/mL (high)) and vehicle-treated iPSCs (control). Red and blue dots represent upregulated (up) and downregulated genes (down), respectively. (**B**) Venn diagram exhibits comparison of DEG numbers between High- and Low-ECR treatment. DEGs were classified as High-specific, Low-specific, common (up), and common (down). (**C**,**D**) Functional network showing the cluster of enriched GO and pathway associated with High-ECR-specific up genes (**C**) and down genes (**D**), respectively (FDR < 0.01).

Pharmacological studies have shown that CR and its main alkaloids exhibit anti-cancer, anti-diabetic, anti-inflammatory, anti-microbial, neuroprotective, and cardioprotective effects [22,23]. Recently, whole-genome transcriptome analyses have clarified the pharmaceutical mechanisms of ECRs. In RAW264.7 cells, pathway clustering analysis showed that top hits for ECRs include cell cycle, pyrimidine metabolism, DNA replication and base excision repair, and p53 signaling pathways [24]. In this study, to identify differentially expressed genes (DEGs) in iPSCs, we profiled gene expression in vehicle-treated iPSCs (control) and iPSCs treated with 5 μg/mL of an ECR (ECR-Low) or 10 μg/mL of an ECR (ECR-High). Each group consisted of five samples (n = 5/group). Approximately 1.38% of genes (310/22,401 genes) in the ECR-Low group were upregulated, and 1.01% of genes (227/22,401 genes) were downregulated compared with the control group (false discovery rate [FDR] < 0.01) (Figure 1A). In the ECR-High group, 3.79% of genes (850/22,401 genes) and 4.1% of genes (919/22,401 genes) were upregulated and downregulated compared with the control group, respectively. A total of 1849 DEGs were found to be regulated by ECR treatment (Table S1). Figure 1B shows the distribution of DEG counts for each group. To investigate the biological functions of these DEG groups, we performed functionally grouped network analysis (Table S2). The results showed that upregulated ECR-High-specific DEGs were involved in cell death-related pathways such as TP53 regulation of cell death gene transcription and the mTOR and Rap1 signaling pathways (Figure 1C), whereas downregulated ECR-High-specific DEGs were involved in TP53 activity regulation through phosphorylation and TP53 activity regulation (Figure 1D). These results suggest that the expression of genes involved in cell death-related pathways was significantly altered upon high-dose ECR treatment.

### 3.2. The ECR Showed Cytotoxicity in iPSCs in Two-Dimensional (2D) and Three-Dimensional (3D) Cultures and Induced Apoptotic Cell Death

**Figure 2:**
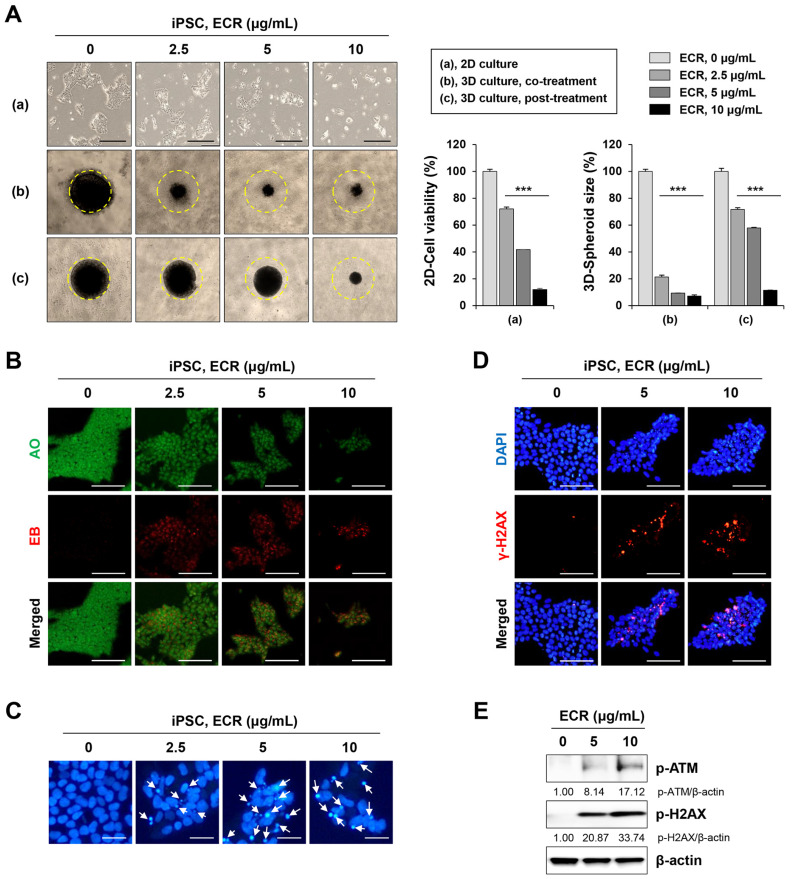
Cytotoxic effect of ECR on iPSCs in 2D and 3D cultures. (**A**) iPSCs were treated with ECR for 24 h, and relative cell viability was determined after crystal violet staining (**a**). For 3D culture, iPSCs were assembled to spheroids and then co-treated (**b**) or post-treated (**c**) with ECR. After 72 h, spheroid size was measured using ImageJ software. Data are shown as means ± SD (n = 3). *** *p* < 0.001 vs. vehicle-treated control. (**B**) iPSCs were treated with ECR for 24 h and stained with AO/EB mixture. (**C**) iPSCs were treated with ECR for 24 h. Apoptotic cells (white arrow) were observed after staining with DAPI solution. (**D**) iPSCs were treated with ECR for 24 h and stained for γ-H2AX foci formation. (**E**) iPSCs were treated with ECR for 24 h, and the protein levels were measured by Western blot analysis. Scale bar = 100 μm.

Because whole-transcriptome analysis revealed that the ECR regulated cell death-related signaling pathways in iPSCs, we investigated the effects of the ECR on cytotoxicity, apoptosis, and DNA damage in iPSCs. In 2D culture, the ECR significantly decreased cell viability in a dose-dependent manner and induced the morphological disruption of iPSCs, whereas no effect was observed for the vehicle control with up to 0.02% DMSO (Figure 2A). In 3D culture, spheroids co-treated with 2.5, 5, and 10 μg/mL of ECR were dramatically reduced in size by approximately 78.7%, 90.7%, and 92.9%, respectively, compared with untreated spheroids. Spheroids post-treated with ECR were also reduced in size in a dose-dependent manner, although to a lesser degree (Figure 2A). To determine the mode of ECR-mediated cell death in iPSCs, ECR-treated and -untreated cells were stained with an AO/EB mixture and observed under fluorescence microscopy. AO penetrates both live and dead cells, binds to their DNA, and emits green fluorescence, whereas EB enters cells whose cell membrane integrity has collapsed and marks their nuclei with red fluorescence. Following treatment with AO/EB, viable cells with intact membranes were evenly stained with green fluorescence in the nuclei, and early apoptotic cells with intact membranes and damaged DNA showed green condensed nuclei. Late apoptotic cells with disrupted membranes exhibited condensed yellow to orange nuclei, and necrotic cells showed condensed red fluorescence [25]. Early and late apoptotic cells in iPSC populations increased gradually in number following ECR treatment in a dose-dependent manner; at concentrations above 5 μg/mL of ECR, nearly all iPSCs exhibited the same pattern as late apoptotic cells (Figure 2B). Next, to observe morphological changes in the nucleus, ECR-treated and -untreated iPSCs were stained with a cell-permeable DNA dye and observed under a fluorescence microscope. ECR-untreated iPSCs showed normal, intact nuclei with light blue fluorescence (Figure 2C). In ECR-treated iPSCs, the proportion of apoptotic nuclei showing nuclear condensation and fragmentation with dark blue fluorescence increased gradually in a dose-dependent manner to 18.2%, 40.7%, and 47.4% at 2.5, 5, and 10 μg/mL of ECR, respectively. Next, we determined whether the ECR induced DNA damage in iPSCs. Genotoxic anti-cancer agents (e.g., doxorubicin and cisplatin) and γ-irradiation cause DNA double-strand breaks and phosphorylate H2AX through ATM phosphorylation, resulting in the accumulation of γ-H2AX foci at the site of DNA damage [26,27,28]. We confirmed that doxorubicin reduced cell viability and increased apoptotic nuclei in iPSCs (Supplementary Figure S1A,B). Doxorubicin markedly increased the phosphorylation of ATM followed by that of H2AX (Supplementary Figure S1C), resulting in the formation of γ-H2AX foci at the region of DNA damage (Supplementary Figure S1D). Investigation of the DNA damage response in ECR-treated iPSCs revealed that ECR treatment markedly increased punctate γ-H2AX foci in the nucleus (Figure 2D) and increased the protein levels of p-ATM and p-H2AX in a dose-dependent manner (Figure 2E). These data collectively demonstrate that the ECR effectively induced apoptotic cell death and DNA damage in iPSCs.

### 3.3. The ECR Induced Intracellular ROS Generation, Mitochondrial Damage, and Caspase-3/-9 Activation in iPSCs

**Figure 3:**
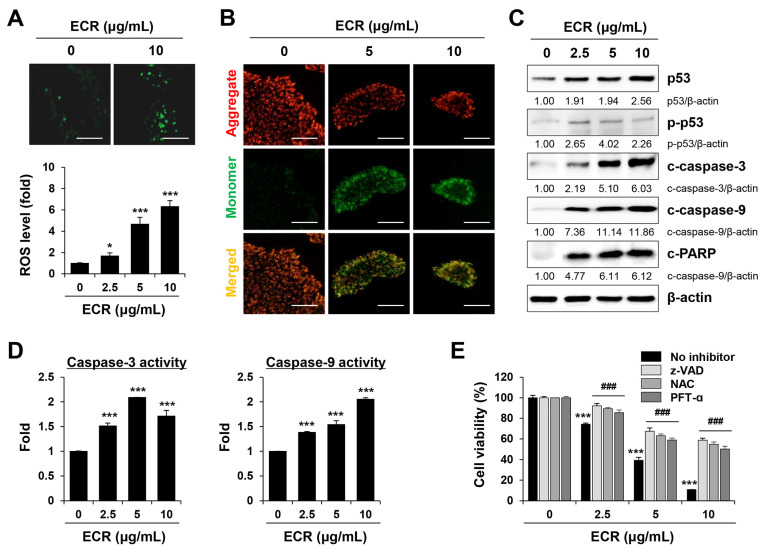
Induction of ROS generation, mitochondrial damage, and caspase-3/-9 activation by ECR in iPSCs. (**A**) iPSCs treated with ECR were detected for oxidative stress. Relative ROS levels compared with ECR-untreated iPSCs were presented as means ± SD (n = 3). (**B**) iPSCs treated with ECR for 12 h were stained with JC-1 for detection of mitochondrial membrane potential. (**C**) Protein levels in ECR-treated iPSCs were measured by Western blot analysis. (**D**) iPSCs were treated with ECR for 24 h, and the caspase-3 and caspase-9 activities were measured. Relative activities compared with WCR-untreated iPSCs were presented as means ± SD (n = 3). (**E**) After pre-treatment with z-VAD (10 μM), NAC (50 μM), and PFT-α (10 μM) for 30 min, iPSCs were exposed to ECR for 24 h. After staining cells, relative cell viability was presented as means ± SD (n = 3). * *p* < 0.05 and *** *p* < 0.001 vs. vehicle-treated control. ### *p* < 0.001 vs. no inhibitor. Scale bar = 100 μm.

CR has beneficial effects against various cancers through the induction of apoptosis [13,29]. CR and its main constituent, berberine, reduce cell viability, alter the expression of anti-apoptotic (e.g., Bcl2 and Mcl-1) and pro-apoptotic (e.g., Bax and Bak) proteins, and enhance caspase-3 activity in many cancer cells, including human gastric cancer, pancreatic cancer, glioblastoma, and osteosarcoma [30]. ROS generation and mitochondrial membrane disruption are also involved in CR- and berberine-induced apoptosis [29,31]. In this study, to determine the mechanism of the ECR-mediated apoptotic death of iPSCs, we assessed the effect of the ECR on the induction of oxidative stress in iPSCs. Green fluorescence intensity corresponding to intracellular ROS levels was significantly elevated by ECR treatment, resulting in an approximately 7.3-fold increase at 10 μg/mL of the ECR, compared with that of control iPSCs (Figure 3A). Next, using the fluorescent dye JC-1, we measured the ECR-mediated alteration of MMP. MMP maintenance is important for generating ATP; a loss of MMP leads to energy depletion and cell death [32]. In cells with a high MMP, JC-1 accumulates in the mitochondria as an aggregate of red fluorescence, whereas in cells with a low MMP, it is observed as a monomeric green fluorescence [33]. Following ECR treatment, JC-1 green fluorescence increased dramatically, whereas JC-1 red fluorescence decreased, indicating that the ECR caused a severe drop in MMP in iPSCs (Figure 3B). In doxorubicin-treated iPSCs, we also observed a significant drop in MMP (Supplementary Figure S1E). Western blotting revealed that in iPSCs, the ECR increased the levels of p53 and p-p53, cleaved forms of caspase-3 and -9, and PARP in a dose-dependent manner (Figure 3C). Consistent with this increase in the active forms of caspase-3 and -9, their activity in iPSCs was increased significantly by ECR treatment (Figure 3D). Pre-treatment with the pan-caspase inhibitor z-VAD, the ROS scavenger NAC, and the p53 inhibitor PFT-ɑ efficiently prevented an ECR-mediated decrease in cell viability in iPSCs, confirming that p53 and caspase activation is critical for ECR-induced apoptosis in iPSCs (Figure 3E).

### 3.4. The ECR Did Not Induce Cell Death or Genotoxicity in iPSC-Diff

**Figure 4:**
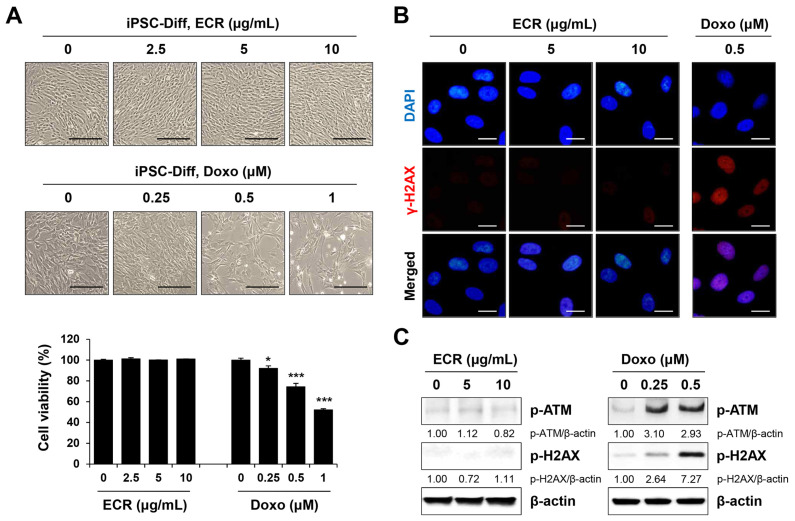
Non-cytotoxic and no DNA-damaging effects of ECR in iPSC-Diff. (**A**) iPSC-Diff were treated with ECR and doxorubicin (Doxo) for 24 h. Relative cell viability compared with ECR- or Doxo-untreated iPSC-Diff was determined and shown as means ± SD (n = 3). (**B**) iPSC-Diff were treated with ECR and Doxo for 24 h, and then γ-H2AX foci were observed. (**C**) The protein levels in ECR and Doxo-treated iPSC-Diff were measured by Western blot analysis. * *p* < 0.05 and *** *p* < 0.001 vs. vehicle-treated control. Scale bar = 100 μm.

Next, to confirm the selective cytotoxicity of the ECR in iPSCs, we examined whether the ECR was non-cytotoxic and non-genotoxic in iPSC-Diff using doxorubicin as a control. Treatment with up to 10 μg/mL of the ECR had no cytotoxic effect on cell morphology or viability in iPSC-Diff, whereas doxorubicin significantly reduced cell proliferation in a dose-dependent manner (Figure 4A). Doxorubicin formed robust γ-H2AX foci in nuclei and markedly increased the protein levels of p-ATM and p-H2AX in iPSC-Diff, at similar levels to those in iPSCs (Figure 4C). By contrast, γ-H2AX foci formation and increased expression of p-ATM and p-H2AX were not observed in ECR-treated iPSC-Diff. These results confirm that the ECR induced cytotoxicity in iPSCs without causing a DNA damage response in iPSC-Diff.

### 3.5. The ECR Selectively Eliminated iPSCs but Not iPSC-Diff in a Mixed Cell Population and Suppressed in Ovo Teratoma Formation

**Figure 5:**
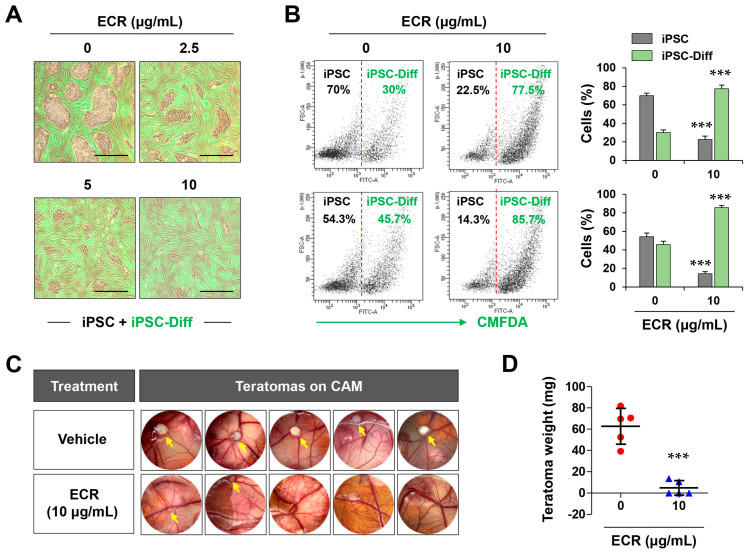
Selective killing of iPSCs in a mixed cell culture with iPSC-Diff and inhibition of teratoma formation by ECR. (**A**) After treating ECR for 24 h with a mixed cell culture of iPSCs and green dye-labeled iPSC-Diff, cells were observed under a fluorescence microscope. (**B**) Various numbers of iPSCs and green dye-labeled iPSC-Diff were co-cultured. After treating ECR for 24 h, the remaining cells were analyzed by flow cytometry. (**C**) On ED day 10, ECR-treated mixed cell population of iPSCs and iPSC-Diff were loaded on CAMs. After 8 days, teratomas formed on CAMs were photographed. (**D**) The weights of teratomas were weighed, and data were expressed as means ± SD (n = 5). *** *p* < 0.001 vs. vehicle-treated control. Scale bar = 100 μm.

Even following differentiation, multi-potent undifferentiated iPSCs may remain in the final cell product and may form teratomas, which is a major difficulty in stem cell-based regenerative medicine. For effective and safe teratoma-free iPSC-based cell therapy, it is essential to selectively and completely remove iPSCs before grafting them into patients, without compromising the viability, functional properties, and genetic stability of iPSC-Diff. Therefore, it is important to optimize the process of removing any remaining iPSCs by adjusting the treatment concentration and time according to the type of differentiated cells and the amount of iPSCs remaining in the final cell product [6]. To determine whether the ECR selectively eliminated residual iPSCs in iPSC-based stem cell therapy, we co-cultured iPSCs and different ratios of CMFDA dye-labeled iPSC-Diff in the same culture plate, followed by ECR treatment. As shown in Figure 5A, unlabeled iPSCs decreased gradually through ECR treatment in a dose-dependent manner, whereas green fluorescence-labeled iPSC-Diff continued to proliferate and showed no cytotoxicity, even at 10 μg/mL of ECR. Selective killing of iPSCs by the ECR was also observed in co-culture with green fluorescence-labeled iPSCs and unlabeled iPSC-Diff (Supplementary Figure S2A). Next, we performed flow cytometry to quantify the remaining iPSCs in a mixed population. iPSCs co-cultured with green fluorescence-labeled iPSC-Diff at different ratios (initial iPSC:iPSC-Diff ratios of 7:3 and 5:5) were treated with 10 μg/mL of the ECR for 24 h, harvested, and analyzed for green fluorescence. In mixed populations, the iPSCs fell from an initial proportion of ~70% to ~22.5% and from an initial proportion of 55% to 14.3% following treatment with 10 μg/mL of ECR. By contrast, green fluorescence-labeled iPSC-Diff increased to ~77.5–85.7% in mixed populations after ECR treatment (Figure 5B). In the presence of the ECR, green fluorescence-labeled iPSCs were significantly decreased (Supplementary Figure S2B). In particular, an initial proportion of ~20% iPSCs was selectively and almost completely removed by ECR treatment. These results indicate that ECR may be useful for preparing teratoma-free cells. Next, to demonstrate the anti-teratoma activity of the ECR, mixed cell populations of iPSCs and iPSC-Diff were loaded onto the CAM of fertilized eggs and then further incubated for 8 days. On ED day 18, ECR-untreated cells formed sizable teratomas on CAMs, whereas ECR-treated cells rarely formed teratomas (Figure 5C). The teratomas from ECR-untreated cells had a volume of 142.11 ± 21.32 mm^3^, whereas those from ECR-treated cells had a volume of 2.45 ± 3.50 mm^3^. Teratomas from ECR-untreated and -treated cells weighed 52.25 ± 16.82 mg and 5.73 ± 6.76 mg, respectively (Figure 5D). Consistent with our previous findings [25], teratomas generated from ECR-untreated cells had three distinct germ layers, including endodermal, mesodermal, and ectodermal tissues, which are differentiated from iPSCs (Supplementary Figure S3). These results indicate that the ECR selectively removed teratoma-forming iPSCs from a mixed population, thereby efficiently suppressing teratoma formation.

### 3.6. Coptisine and Berberine in the ECR Exhibited Selective Cytotoxicity in iPSCs but Not in iPSC-Diff

Various secondary metabolites have been isolated from CR through chemical investigation, among which alkaloids such as berberine, coptisine, jatrorrhizine, and palmatine are the main active components [24]. Berberine has been reported to have hypoglycemic and hypolipidemic effects and to suppress various cancers and the inflammatory response [30,34,35]. Coptisine has also been reported to have anti-cancer, anti-inflammatory, and anti-diabetic effects [36,37], and palmatine has been reported to have neuroprotective and anti-inflammatory effects [38]. However, as with CR, the anti-teratoma activities of these components have not been explored.

**Figure 6:**
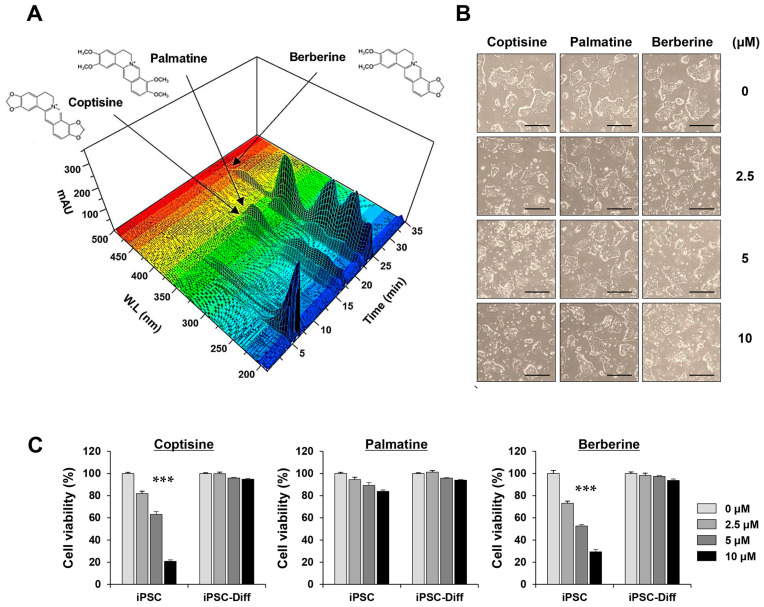
Three-dimensional chromatogram of three major compounds in ECR and their cytotoxic effects on iPSCs and iPSC-Diff. (**A**) Using UHPLC-DAD system, coptisine, palmatine, and berberine were identified in ECR. (**B**) After treating iPSCs with coptisine, palmatine, and berberine for 24 h up to 10 μM, morphological changes were observed. (**C**) Relative cell viability was determined after crystal violet staining and presented as means ± SD (n = 3). *** *p* < 0.001 vs. vehicle-treated control. Scale bar = 100 μm.

In this study, we used an established UHPLC-DAD method to analyze coptisine, palmatine, and berberine in the ECR, which had peak retention times of 17.54, 21.81, and 23.56 min, respectively (Figure 6A). The content measurements for these three compounds are listed in Table 3. Next, to identify the major constituents in the ECR contributing to the selective killing of iPSCs, we treated iPSCs and iPSC-Diff with the three compounds at 2.5, 5, and 10 μM for 24 h. As shown in Figure 6B, coptisine and berberine significantly induced the morphological disruption of iPSCs, whereas palmatine induced little morphological change, even at 10 μM. Cell viability quantitation revealed that coptisine and berberine decreased the viability of iPSCs in a dose-dependent manner, with little toxicity to iPSC-Diff. By contrast, palmatine showed no serious cytotoxicity in either iPSCs or iPSC-Diff. Together, these results suggest that coptisine and berberine were the major components in the ECR contributing to the selective removal of iPSCs.

Whole-genome transcriptome analyses were recently performed to clarify the pharmaceutical mechanisms of ECRs and identify the functional differences among the main CR alkaloids. In a pathway clustering analysis of RAW264.7 cells, the top hits for ECRs included the cell cycle, pyrimidine metabolism, DNA replication and base excision repair, and p53 signaling pathways, and berberine and coptisine showed similar trends to the ECR [24]. Consistent with our transcriptome analysis results, the ECR, berberine, and coptisine induced severe cytotoxicity in several cell lines, including HepG2, 3T3-L1, and Raw264.7 cells, whereas palmatine showed no cytotoxicity even at 100 μM. Our transcriptome analysis in iPSCs also revealed that the ECR regulated pathways related to the cell cycle, cell proliferation, and cell death, which is similar to the pathways related to Raw264.7 cells, and that the ECR exerted selective cytotoxic effects in iPSCs but not in iPSC-Diff, along with berberine and coptisine. This study is the first to perform a transcriptome analysis of ECR-treated iPSCs and to demonstrate the beneficial effects of an ECR on the inhibition of iPSC-derived teratoma formation. To further understand the pharmacological mechanisms involved in the anti-teratoma activity of ECR, we intend to perform transcriptome analysis of the main ECR alkaloids in iPSCs and identify functional differences and similarities in a future study. In addition, for use in regenerative medicine, it is necessary to determine whether the functions and characteristics of differentiated target cells are not adversely affected by treatment with ECR or its components. If these issues are addressed, ECR could be used as an effective anti-teratoma agent in both clinical and laboratory applications.

## Figures and Tables

**Table 1:** UHPLC condition for analysis.

Parameter		Analytical Conditions	
Column temperature		30 °C	
UV		DAD at 350 nm	
Spectra range		190 to 500 nm	
Injection volume		2 µL	
Flow rate		0.5 mL/min	
Column		Phenomenex Luna C_18_ (4.6 × 250 mm, 5 µm)	
Sample reconstruction		2 mg/mL in MeOH	
Mobile phase	Time (min)	A (%) (0.1% formic acid in water)	B (%) (Acetonitrile)
	0	72	28
	35	90	10
	50	Washing and equilibrium	

**Table 2:** Quantitation parameters.

Constituent	Linear Range (µg/mL)	Regression Equation ^(a)^		Correlation Coefficient, *r*^2^	LOD (µg/mL)	LOQ (µg/mL)
		Slope	Intercept			
Coptisine	2–200	24,405	8.067	0.9999	0.541	1.638
Palmatine	5–200	15,315	5.335	0.9999	0.890	2.696
Berberine	1–200	35,455	15.435	0.9999	0.263	0.797

^(a)^ y = ax + b, y, and x indicate the peak area and the concentration of sample (μg/mL), respectively.

**Table 3:** The amount of each constituent in ECR.

Compound	Content (mg/g)
Coptisine	31.62 ± 0.09
Palmatine	48.56 ± 0.23
Berberine	84.01 ± 0.11

## Data Availability

Total RNA sequencing data were deposited in the NCBI Gene Expression Omnibus (GEO, https://www.ncbi.nlm.nih.gov/geo/ accessed on 15 May 2023) with accession number GSE225709.

## Supplementary Materials

The following supporting information can be downloaded at https://www.mdpi.com/article/10.3390/nu15102364/s1. Figure S1: Induction of apoptotic cell death by doxorubicin in iPSCs; Figure S2: Selective killing of iPSCs in a mixed population with iPSC-Diff at various ratios; Figure S3: Histological analysis of teratomas generated from ECR-untreated cells after hematoxylin-eosin (H&E) staining; Figure S4: Uncropped western blot image; Table S1: Altered expressed gene list in iPSC treated with high and low dose ECR; Table S2: Biological function of DEG groups.
