# Supplementary material for: An Ethanol Extract of Coptidis rhizoma Induces Apoptotic Cell Death in Induced Pluripotent Stem Cells and Suppresses Teratoma Formation
Source: Nutrients. 2023 May 18;15(10):2364. doi: 10.3390/nu15102364 (PMC10221726; doi:10.3390/nu15102364)
Supplement: Supplementary file 1 [file nutrients-15-02364-s001.zip › Supplementary Figure S1, S2, S3, S4.pdf]

Supplementary Materials for

An Ethanol Extract of Coptidis rhizoma induces Apoptotic Cell Death in Induced Pluripotent Stem Cells and Suppresses Teratoma Formation

Aeyung Kim, Su-Jin Baek, Sarah Shin, Seo-Young Lee and Sun-Ku Chung

*Corresponding Author: Aeyung Kim (aykim71@kiom.re.kr)

This PDF file includes:

Figure S1 to Figure S4

Figure S1

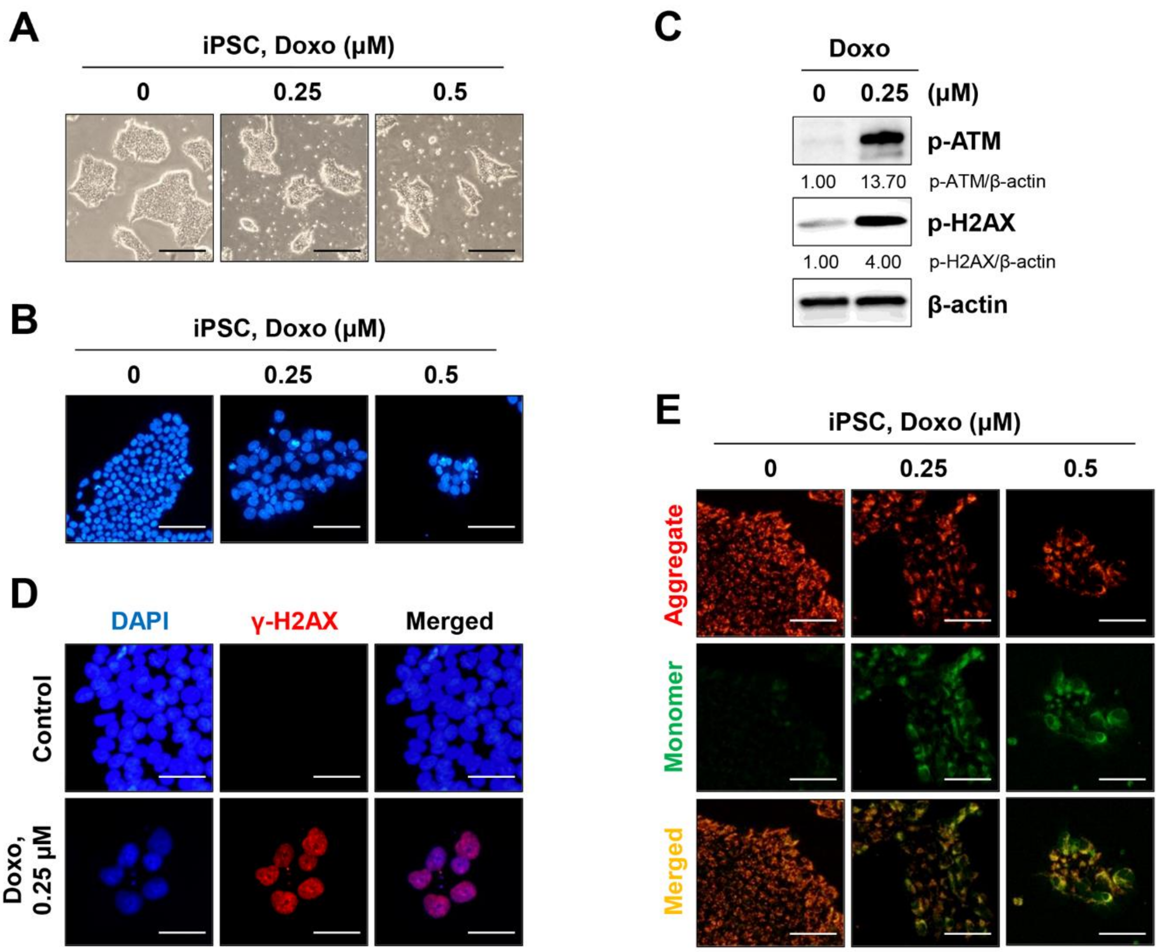

Figure S1. Induction of apoptotic cell death by doxorubicin in iPSCs. **(A)** After treatment of iPSCs with 0.25 and 0.5 µM doxorubicin for 24 h, cells were observed under an inverted microscope. **(B)** Apoptotic cell death was detected by DAPI staining. **(C)** iPSCs were treated with 0.25 µM doxorubicin for 6 h. Protein levels of p-ATM and p-H2AX were determined by western blotting. **(D)** γ-H2AX foci formation in doxorubicin-treated iPSCs was observed under a fluorescence microscope. **(E)** iPSCs were treated with doxorubicin for 6 h and then stained with JC-1. Disruption of mitochondrial membrane potential was observed under a fluorescence microscope. Scale bar = 100 µm

Figure S2

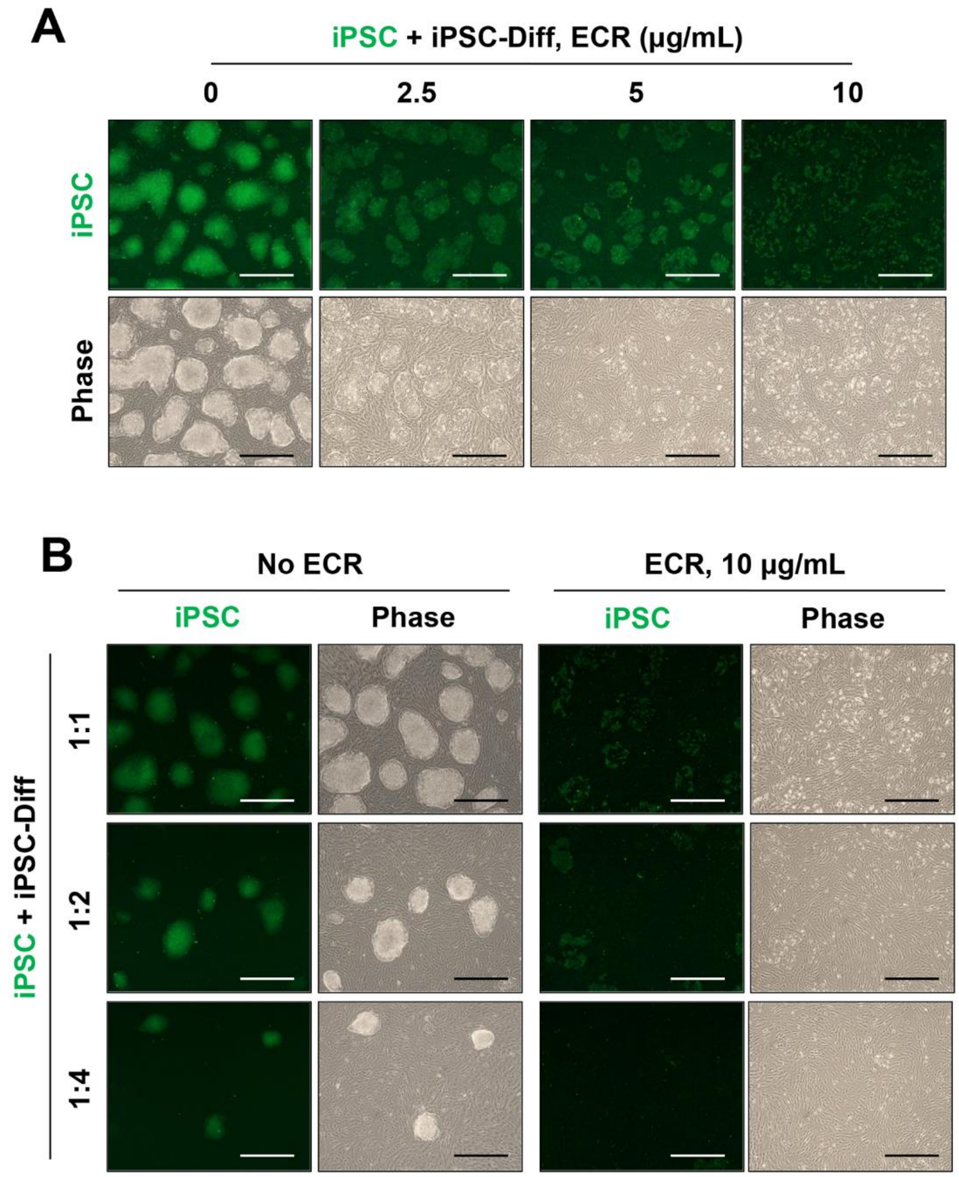

Figure S2. Selective killing of iPSCs in a mixed population with iPSC-Diff at various ratios. **(A)** CellTracker green CMFDA dye-labeled iPSCs and un-labeled iPSC-Diff were co-cultured at a ratio of 1:1 and treated with 2.5, 5, and 10 µg/mL ECR for 24 h. **(B)** Green dye-labeled iPSCs and un-labeled iPSC-Diff were co-cultured at a ratio of 1:1, 1:2, and 1:4, and treated with 10 µg/mL ECR for 24 h. Remaining green-labeled iPSCs were observed under a fluorescence microscope. Scale bar = 100 µm

Figure S3

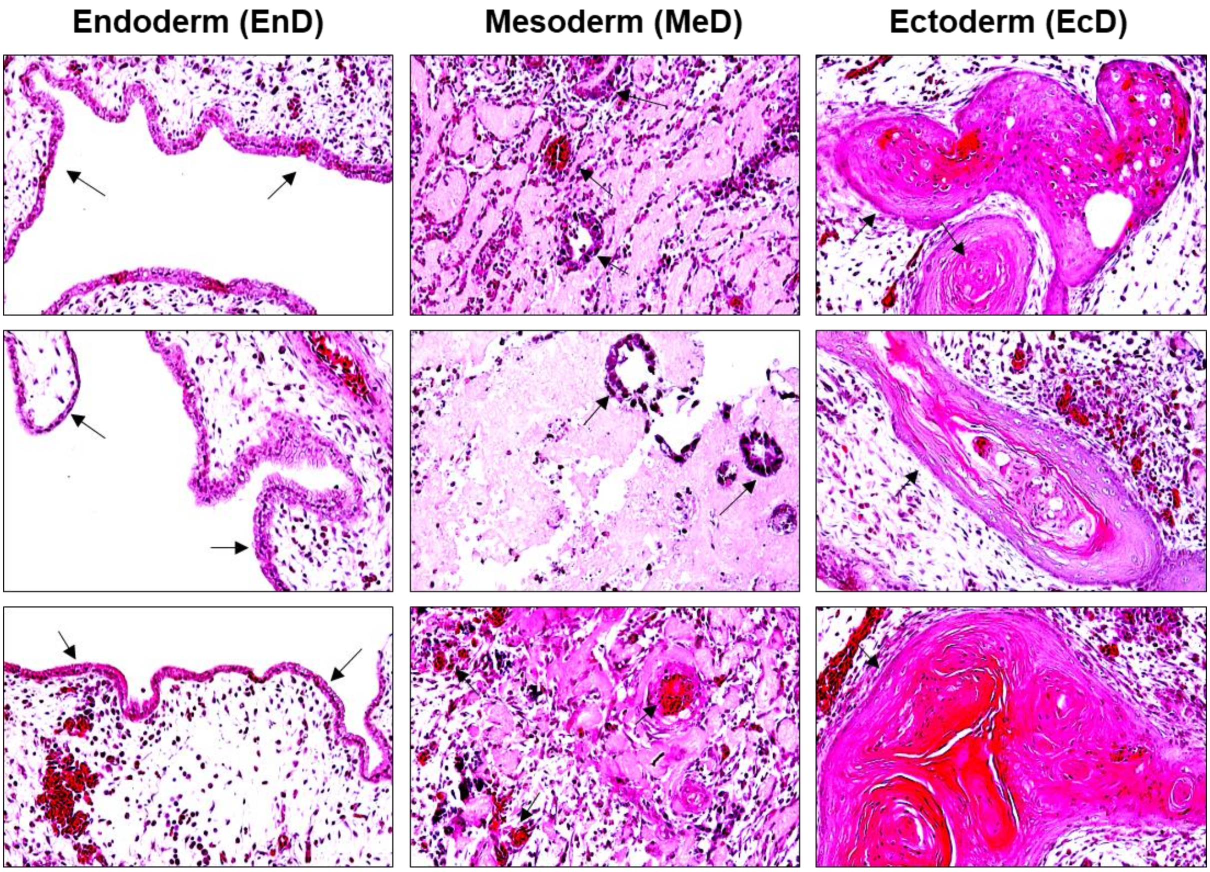

Figure S3. Histological analysis of teratomas generated from ECR-untreated cells after hematoxylin-eosin (H&E) staining. Arrows indicate cells of three germ layers, including endoderm (EnD), mesoderm (MeD), and ectoderm (EcD).

Figure S4

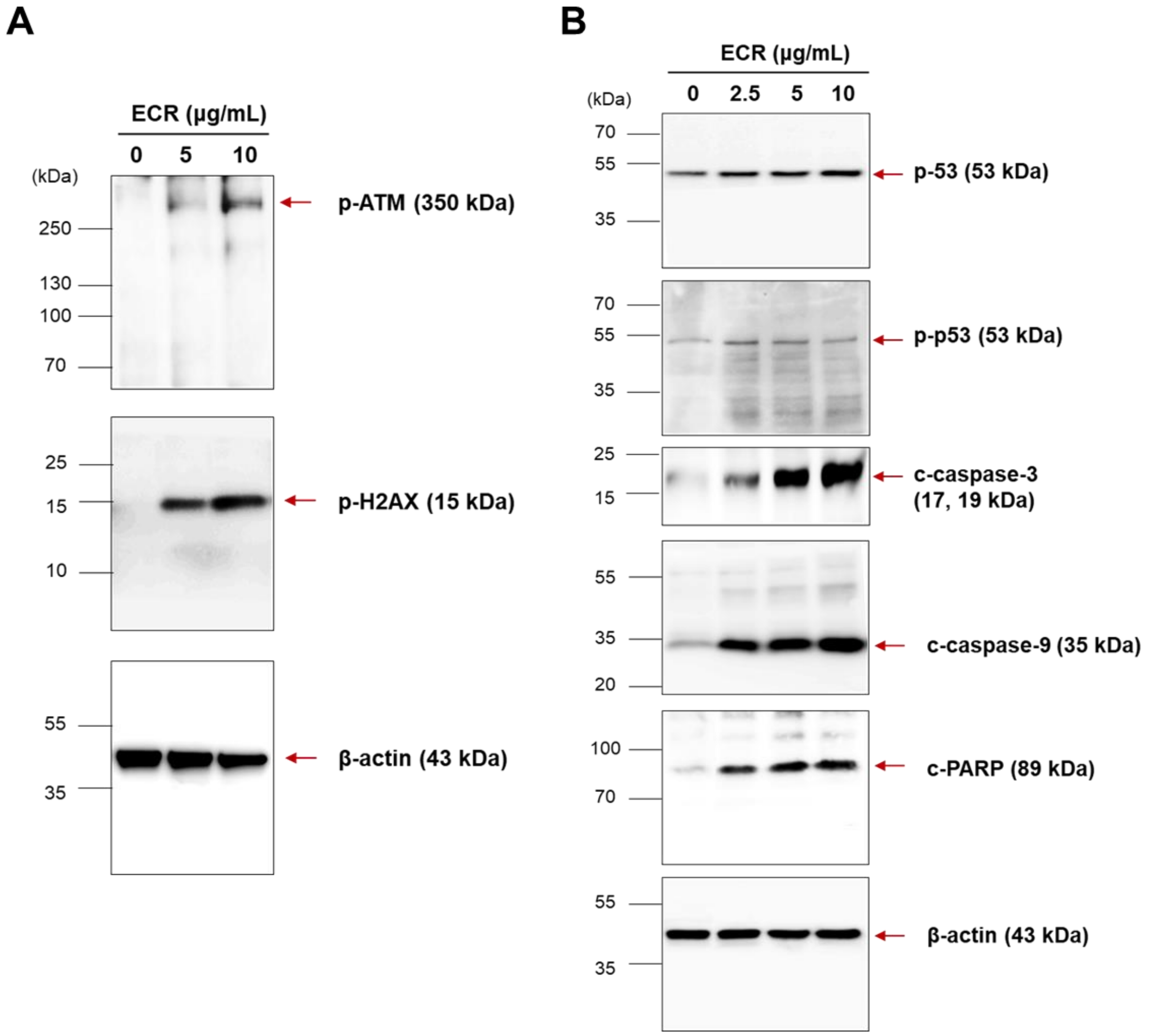

A
ECR (µg/mL)
0
5
10
(kDa)
250
130
100
70
p-ATM (350 kDa)
25
15
10
p-H2AX (15 kDa)
55
35
β-actin (43 kDa)
B
ECR (µg/mL)
(kDa)
0
2.5
5
10
70
55
35
p-53 (53 kDa)
p-p53 (53 kDa)
25
15
c-caspase-3
(17, 19 kDa)
35
20
c-caspase-9 (35 kDa)
100
c-PARP (89 kDa)
β-actin (43 kDa)

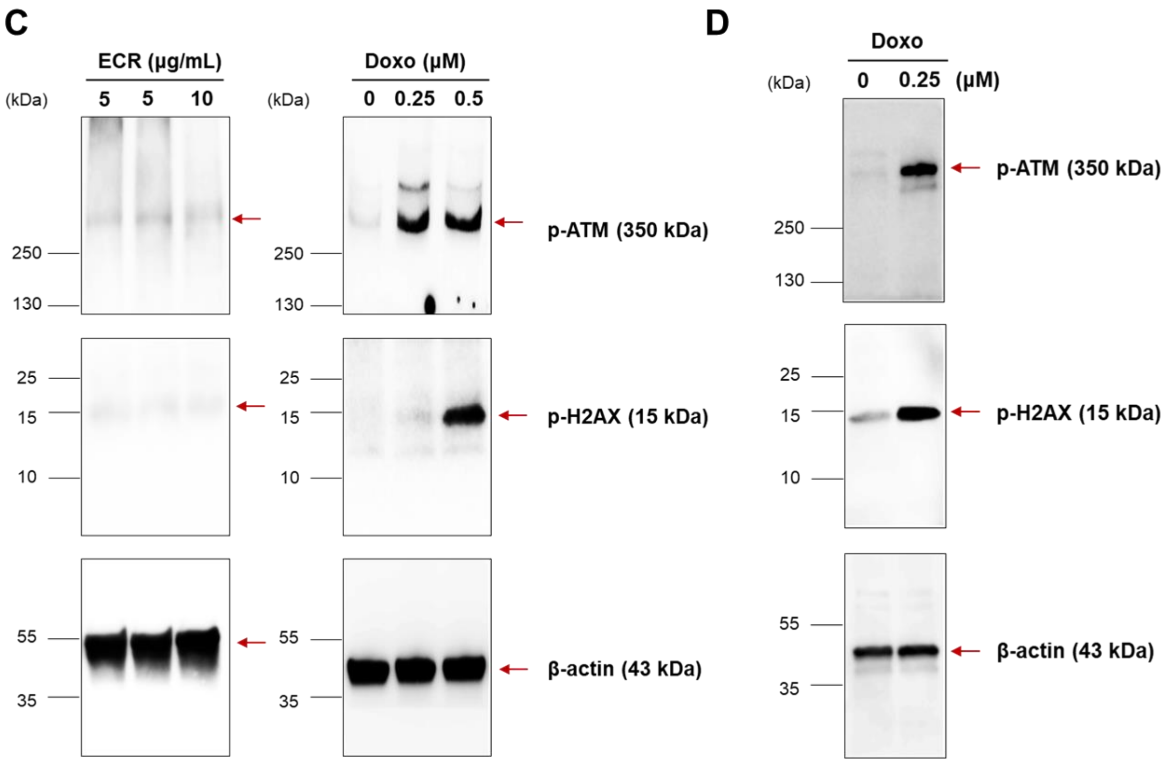

Figure S4. Uncropped western blot images corresponding to Figure 2E **(A)**, Figure 3C **(B)**, Figure 4C **(C)**, and Figure S1C **(D)**.
