# Supplementary material for: An Ethanol Extract of Coptidis rhizoma Induces Apoptotic Cell Death in Induced Pluripotent Stem Cells and Suppresses Teratoma Formation
Source: Nutrients. 2023 May 18;15(10):2364. doi: 10.3390/nu15102364 (PMC10221726; doi:10.3390/nu15102364)
Supplement: Supplementary file 1 [file nutrients-15-02364-s001.zip › Supplementary Table S1, S2.pdf]

Table S1. Altered expressed gene list in iPSC treated with high and low dose ECR

| Category | | High_vs_control | | Low_vs_control | |
|---|---|---|---|---|---|
| *Type* | *gene* | *FC(fold change)* | *FDR* | *FC(fold change)* | *FDR* |
| **High_specific_down** | FAM83D | -5.665651115 | 0.001610166 | 0.353951847 | 0.864010027 |
| **High_specific_down** | ALS2 | -5.643689802 | 0.003428432 | -0.395073299 | 0.885931229 |
| **High_specific_down** | ELMO1 | -5.576963518 | 0.001523775 | -3.515760215 | 0.056479398 |
| **High_specific_down** | MARS2 | -5.417636708 | 0.004981369 | -0.279789611 | 0.921483665 |
| **High_specific_down** | ADAT3 | -5.413138146 | 0.006372026 | -1.380750949 | 0.520451701 |
| **High_specific_down** | C2CD4C | -5.314853912 | 0.005269252 | -1.301348688 | 0.564277861 |
| **High_specific_down** | TCAP | -3.890503964 | 0.004617632 | -1.733962556 | 0.309349599 |
| **High_specific_down** | AC069444.1 | -3.49409388 | 0.003333909 | -2.423298422 | 0.079498927 |
| **High_specific_down** | C17orf97 | -2.936097739 | 0.007540935 | -0.839529326 | 0.58584825 |
| **High_specific_down** | UHRF1 | -2.788818503 | 0.006866209 | -0.857561766 | 0.530228782 |
| **High_specific_down** | LINC01668 | -2.699329086 | 0.001102329 | -1.690550985 | 0.213683315 |
| **High_specific_down** | S100A13 | -2.651505812 | 0.0004442 | -1.424711414 | 0.253104845 |
| **High_specific_down** | SSR1 | -2.331597139 | 0.007503625 | -0.543322037 | 0.671840554 |
| **High_specific_down** | TECPR1 | -2.28058828 | 3.16E-11 | -1.232293186 | 0.012120168 |
| **High_specific_down** | SMO | -2.108599976 | 0.007239433 | -0.824819012 | 0.471068066 |
| **High_specific_down** | FHAD1-AS1 | -2.022435701 | 0.001137076 | -0.92000355 | 0.190587957 |
| **High_specific_down** | AC244517.4 | -1.873231487 | 7.26E-05 | -0.808918639 | 0.242033094 |
| **High_specific_down** | CRTC3-AS1 | -1.868028972 | 0.003247327 | -1.010473058 | 0.213527715 |
| **High_specific_down** | PLXNB2 | -1.745995387 | 0.00258572 | -0.613692158 | 0.476550324 |
| **High_specific_down** | MAN2A1 | -1.736252369 | 0.000657706 | -0.948227661 | 0.145210203 |
| **High_specific_down** | AC091100.1 | -1.701726017 | 6.43E-07 | -0.571505329 | 0.192932694 |
| **High_specific_down** | AC005544.2 | -1.638890214 | 1.02E-05 | -1.208793144 | 0.075410887 |
| **High_specific_down** | AC100786.1 | -1.635382362 | 0.000131717 | -0.760303939 | 0.304364105 |
| **High_specific_down** | MGST2 | -1.600948118 | 0.009638298 | -0.903683671 | 0.284099415 |
| **High_specific_down** | RELL1 | -1.577381239 | 0.006445857 | -1.234297223 | 0.101035516 |
| **High_specific_down** | AC007728.2 | -1.513084484 | 0.002179537 | -0.858076889 | 0.212587261 |
| **High_specific_down** | ARL2BPP10 | -1.506369375 | 0.001099065 | -1.298574236 | 0.021140819 |
| **High_specific_down** | AL845552.2 | -1.472409263 | 3.69E-05 | -0.256325346 | 0.696967977 |
| **High_specific_down** | MGC4859 | -1.419772098 | 0.001134993 | -0.602420191 | 0.171371343 |
| **High_specific_down** | C12orf57 | -1.402486935 | 0.003314867 | -1.110428474 | 0.092074983 |
| **High_specific_down** | HSPA4 | -1.372368511 | 0.002554038 | -0.432638653 | 0.69838776 |
| **High_specific_down** | AC092384.2 | -1.327135633 | 0.003122309 | -1.095106464 | 0.0321171 |
| **High_specific_down** | CR392039.4 | -1.309580668 | 1.23E-05 | -0.955251812 | 0.021411657 |
| **High_specific_down** | AJ003147.2 | -1.270473213 | 0.002762653 | -1.011453776 | 0.048682637 |
| **High_specific_down** | AC005339.1 | -1.258867208 | 0.000191937 | -0.645532325 | 0.154478105 |
| **High_specific_down** | AC010327.4 | -1.257858647 | 3.22E-05 | -0.750234872 | 0.149732387 |
| **High_specific_down** | UBE2Q2P1 | -1.244248149 | 4.60E-06 | -0.828763577 | 0.016255865 |
| **High_specific_down** | MYB-AS1 | -1.236565486 | 0.000701457 | -0.706485911 | 0.20671477 |
| **High_specific_down** | TATDN2 | -1.219703749 | 7.65E-07 | -0.840564097 | 0.017491627 |
| **High_specific_down** | RBM38 | -1.219591158 | 0.003303645 | -0.311878565 | 0.68556574 |
| **High_specific_down** | AC010336.2 | -1.211392657 | 0.000410229 | -0.869167343 | 0.178762819 |
| **High_specific_down** | AL121758.1 | -1.20573203 | 0.005469883 | -0.784109119 | 0.188765262 |
| **High_specific_down** | AFDN-DT | -1.200231533 | 3.64E-06 | -0.621040162 | 0.031515728 |
| **High_specific_down** | PAQR7 | -1.177259901 | 0.008507194 | -1.119019487 | 0.114487073 |
| **High_specific_down** | DDX24 | -1.166551737 | 7.73E-05 | -0.540405171 | 0.284049842 |
| **High_specific_down** | AC068987.3 | -1.165548668 | 1.59E-11 | -0.59781005 | 0.016182356 |
| **High_specific_down** | ZNF565 | -1.151491507 | 0.006372026 | -0.242420375 | 0.770097382 |
| **High_specific_down** | TBL1X | -1.14655292 | 0.000161029 | -0.848523925 | 0.029118561 |
| **High_specific_down** | LRRC8A | -1.141422582 | 0.000369404 | -0.603937703 | 0.183444278 |
| **High_specific_down** | AC008747.1 | -1.133672108 | 0.004351909 | -0.494753244 | 0.444264412 |

| Category | | High_vs_control | | Low_vs_control | |
|---|---|---|---|---|---|
| *Type* | *gene* | *FC(fold change)* | *FDR* | *FC(fold change)* | *FDR* |
| **High_specific_down** | ITFG2-AS1 | -1.132549889 | 2.46E-06 | -0.546015568 | 0.126841658 |
| **High_specific_down** | LPIN3 | -1.119588763 | 2.72E-10 | -0.623630141 | 0.100269483 |
| **High_specific_down** | AC022150.3 | -1.108430076 | 8.06E-06 | -0.612587526 | 0.053486794 |
| **High_specific_down** | AL359317.1 | -1.081347619 | 0.003401905 | -0.789030681 | 0.297257218 |
| **High_specific_down** | AL121830.1 | -1.078156065 | 0.006910105 | -0.398750839 | 0.551752781 |
| **High_specific_down** | SMAD1 | -1.072840338 | 0.003114412 | -0.558538637 | 0.239828317 |
| **High_specific_down** | PDZD8 | -1.066696794 | 4.27E-07 | -0.616892045 | 0.035727809 |
| **High_specific_down** | PYCARD-AS1 | -1.051424556 | 3.65E-07 | -0.565659501 | 0.065687563 |
| **High_specific_down** | AL807752.2 | -1.044404441 | 4.46E-20 | -0.519435408 | 0.02479182 |
| **High_specific_down** | DENND1A | -1.030483716 | 1.55E-07 | -0.629713824 | 0.012911674 |
| **High_specific_down** | HMGB1 | -1.02128806 | 0.003619662 | -0.726900688 | 0.104898987 |
| **High_specific_down** | CLHC1 | -1.017684248 | 0.001699112 | -0.56418197 | 0.212001638 |
| **High_specific_down** | PHLPP2 | -1.01585401 | 8.69E-12 | -0.482713391 | 0.012178395 |
| **High_specific_down** | CSRP3-AS1 | -1.005944231 | 0.00084345 | -0.883337946 | 0.011760078 |
| **High_specific_down** | JAKMIP2 | -1.003994168 | 1.09E-08 | -0.608657682 | 0.031876252 |
| **High_specific_down** | IER3-AS1 | -1.003677473 | 3.50E-11 | -0.529844986 | 0.062807153 |
| **High_specific_down** | KLHL18 | -1.0015578 | 0.009301487 | -1.141478559 | 0.016078774 |
| **High_specific_down** | PRICKLE3 | -0.995841177 | 0.000922524 | -0.447968004 | 0.380565724 |
| **High_specific_down** | AC008569.1 | -0.970045336 | 0.000116293 | -0.701793927 | 0.025643345 |
| **High_specific_down** | RNU2-27P | -0.955771143 | 9.39E-05 | -0.848762857 | 0.013231177 |
| **High_specific_down** | AP003396.3 | -0.951599727 | 2.05E-07 | -0.306403833 | 0.369783199 |
| **High_specific_down** | AL355581.1 | -0.949387609 | 0.000372633 | -0.675410216 | 0.024970644 |
| **High_specific_down** | TPGS2 | -0.94632514 | 0.000157235 | -0.590834102 | 0.105143352 |
| **High_specific_down** | AC090772.4 | -0.944529816 | 1.05E-05 | -0.456990649 | 0.119024215 |
| **High_specific_down** | PLP2 | -0.935593294 | 0.003024031 | -0.496798988 | 0.264656615 |
| **High_specific_down** | AC010336.1 | -0.932196691 | 4.13E-06 | -0.460707399 | 0.168824889 |
| **High_specific_down** | NR2C2AP | -0.92953481 | 7.73E-06 | -0.557567494 | 0.045714085 |
| **High_specific_down** | RFC5 | -0.92832503 | 6.18E-07 | -0.470880858 | 0.093388951 |
| **High_specific_down** | LYRM2 | -0.92510112 | 0.000170964 | -0.56784989 | 0.084237664 |
| **High_specific_down** | BSG | -0.917277036 | 6.34E-05 | -0.559733947 | 0.071871696 |
| **High_specific_down** | AL161663.2 | -0.912128137 | 0.000151668 | -0.588720345 | 0.0413443 |
| **High_specific_down** | JSRP1 | -0.908311045 | 6.19E-09 | -0.385270575 | 0.056479398 |
| **High_specific_down** | XKR6 | -0.90709168 | 0.005792963 | -0.08556708 | 0.930247026 |
| **High_specific_down** | USP3-AS1 | -0.902350321 | 6.83E-05 | -0.644963839 | 0.058611293 |
| **High_specific_down** | AC006942.1 | -0.898084651 | 4.20E-09 | -0.475872078 | 0.040397488 |
| **High_specific_down** | BRD7 | -0.895281782 | 0.000582656 | -0.643835521 | 0.055471481 |
| **High_specific_down** | TSSK2 | -0.894494399 | 0.001377661 | -1.144013229 | 0.028367579 |
| **High_specific_down** | SLC15A3 | -0.89315535 | 0.002469937 | -0.793571536 | 0.043426881 |
| **High_specific_down** | PIAS1 | -0.891795793 | 5.85E-06 | -0.511990481 | 0.020900852 |
| **High_specific_down** | BCCIP | -0.88894518 | 0.000377737 | -0.459232131 | 0.442001685 |
| **High_specific_down** | ANGPTL6 | -0.885918031 | 2.45E-05 | -0.518023911 | 0.011810825 |
| **High_specific_down** | EPB41 | -0.885898813 | 7.28E-09 | -0.562819548 | 0.017823521 |
| **High_specific_down** | CCDC152 | -0.885246265 | 0.000304819 | -0.452837279 | 0.247571446 |
| **High_specific_down** | INKA2 | -0.881514302 | 0.002670491 | -0.498602095 | 0.45347298 |
| **High_specific_down** | AL160270.1 | -0.878632074 | 1.59E-12 | -0.445033542 | 0.043427211 |
| **High_specific_down** | RANBP1 | -0.876645322 | 1.23E-08 | -0.408180525 | 0.035012169 |
| **High_specific_down** | AC103858.3 | -0.87509981 | 0.007726833 | -0.402223357 | 0.404832404 |
| **High_specific_down** | CKB | -0.872319501 | 0.004485825 | -0.583332582 | 0.089846785 |
| **High_specific_down** | AC092143.2 | -0.871996879 | 4.34E-05 | -0.028622969 | 0.970962849 |
| **High_specific_down** | ACTN4 | -0.869776111 | 3.27E-07 | -0.556772549 | 0.017809883 |
| **High_specific_down** | AC138466.2 | -0.863493013 | 0.004620075 | -0.226017748 | 0.686247168 |
| **High_specific_down** | AC026771.1 | -0.86054562 | 2.41E-11 | -0.56581796 | 0.047757415 |

| Category | | High_vs_control | | Low_vs_control | |
|---|---|---|---|---|---|
| *Type* | *gene* | *FC(fold change)* | *FDR* | *FC(fold change)* | *FDR* |
| **High_specific_down** | BOK-AS1 | -0.851069076 | 1.70E-05 | -0.367713218 | 0.29637933 |
| **High_specific_down** | AC002463.1 | -0.850795592 | 0.007783126 | -0.676472054 | 0.264656615 |
| **High_specific_down** | ERLNC1 | -0.842555674 | 2.46E-06 | -0.229679062 | 0.554235987 |
| **High_specific_down** | AL049829.2 | -0.841475706 | 6.09E-06 | -0.47775506 | 0.034308628 |
| **High_specific_down** | CEP290 | -0.841114757 | 0.007666427 | -0.292397423 | 0.620942054 |
| **High_specific_down** | AIDA | -0.84027931 | 0.003863633 | -0.706215696 | 0.021313858 |
| **High_specific_down** | TRIM25 | -0.838691599 | 0.003622444 | -0.389529769 | 0.398047008 |
| **High_specific_down** | Z99716.1 | -0.836679654 | 0.001566602 | -0.323341748 | 0.296268411 |
| **High_specific_down** | AP001029.2 | -0.836446977 | 5.53E-06 | -0.431069647 | 0.149792179 |
| **High_specific_down** | AC135048.4 | -0.83485923 | 2.55E-08 | -0.402443174 | 0.055570779 |
| **High_specific_down** | U47924.1 | -0.834092469 | 2.77E-05 | -0.21359377 | 0.672027899 |
| **High_specific_down** | SLC9A5 | -0.832812306 | 5.52E-11 | -0.460682431 | 0.01879541 |
| **High_specific_down** | NEFM | -0.832630946 | 0.002092808 | -0.450559576 | 0.290674819 |
| **High_specific_down** | PDGFRL | -0.83141283 | 0.003572693 | -0.314536759 | 0.575206452 |
| **High_specific_down** | AC108451.2 | -0.830519534 | 0.001865896 | -0.580611477 | 0.099531633 |
| **High_specific_down** | STK16 | -0.827213128 | 0.001228939 | -0.603877381 | 0.072568043 |
| **High_specific_down** | AP000943.2 | -0.825652341 | 1.18E-05 | -0.52943055 | 0.036540957 |
| **High_specific_down** | RUVBL1 | -0.823314423 | 3.93E-11 | -0.52416982 | 0.02374798 |
| **High_specific_down** | AL139398.1 | -0.822748416 | 8.80E-06 | -0.492744768 | 0.062479067 |
| **High_specific_down** | AP000688.1 | -0.821731042 | 2.70E-08 | -0.43349245 | 0.116580553 |
| **High_specific_down** | DUSP6 | -0.821514415 | 0.000133434 | -0.45031272 | 0.145986398 |
| **High_specific_down** | CDIP1 | -0.820258278 | 0.006353783 | -0.882896589 | 0.029260526 |
| **High_specific_down** | AL121603.2 | -0.817830998 | 0.000340305 | -0.682665667 | 0.022510952 |
| **High_specific_down** | MPP5 | -0.816387557 | 0.000171866 | -0.54762681 | 0.053457132 |
| **High_specific_down** | TMEM147-AS1 | -0.812128422 | 1.25E-07 | -0.469210586 | 0.049017425 |
| **High_specific_down** | CRB2 | -0.811366347 | 5.35E-05 | -0.281651701 | 0.432458087 |
| **High_specific_down** | AL136309.3 | -0.809379792 | 0.001099065 | -0.392940754 | 0.332562425 |
| **High_specific_down** | MCF2L2 | -0.807640275 | 0.00026637 | -0.312897127 | 0.465370202 |
| **High_specific_down** | CDH23 | -0.802999023 | 1.30E-06 | -0.353482632 | 0.15280389 |
| **High_specific_down** | DNALI1 | -0.801748682 | 0.000376025 | -0.401905648 | 0.190609749 |
| **High_specific_down** | THAP11 | -0.801485983 | 0.005367329 | -0.486695464 | 0.195601893 |
| **High_specific_down** | AC009118.2 | -0.800310364 | 0.000226525 | -0.449808132 | 0.178517478 |
| **High_specific_down** | AC011472.3 | -0.798261345 | 1.32E-06 | -0.512722236 | 0.037125308 |
| **High_specific_down** | AC016722.2 | -0.795563877 | 2.57E-10 | -0.392705266 | 0.06280974 |
| **High_specific_down** | GFAP | -0.794151095 | 0.000376057 | -0.46958974 | 0.154009842 |
| **High_specific_down** | AC114291.1 | -0.791928599 | 7.75E-05 | -0.537850728 | 0.022044189 |
| **High_specific_down** | ANKRD36BP1 | -0.78780265 | 7.86E-06 | -0.435204885 | 0.020900852 |
| **High_specific_down** | CLBA1 | -0.786395509 | 1.18E-07 | -0.467965165 | 0.028130519 |
| **High_specific_down** | TCTE1 | -0.782838898 | 5.25E-05 | -0.511535387 | 0.028130519 |
| **High_specific_down** | C22orf15 | -0.7815016 | 1.31E-06 | -0.528712077 | 0.032985473 |
| **High_specific_down** | H2AC16 | -0.78133084 | 0.003190931 | -0.182944776 | 0.746480696 |
| **High_specific_down** | AC016825.1 | -0.781311696 | 1.99E-05 | -0.586500529 | 0.010341544 |
| **High_specific_down** | HADHA | -0.779067516 | 0.00657364 | -0.819480689 | 0.011167387 |
| **High_specific_down** | RABL3 | -0.778391934 | 0.000881971 | -0.51727328 | 0.127454965 |
| **High_specific_down** | AC018695.9 | -0.77821018 | 0.00890026 | -0.797947936 | 0.053248215 |
| **High_specific_down** | RFXANK | -0.776363094 | 0.000486462 | -0.455449388 | 0.223269907 |
| **High_specific_down** | NAA15 | -0.772120492 | 0.000267624 | -0.307146178 | 0.381018517 |
| **High_specific_down** | PCBP1-AS1 | -0.771067286 | 3.73E-15 | -0.37424949 | 0.047680152 |
| **High_specific_down** | AL035252.4 | -0.768293712 | 0.000204266 | -0.523599417 | 0.051309485 |
| **High_specific_down** | MED22 | -0.766548506 | 0.00065855 | -0.43762182 | 0.218558891 |
| **High_specific_down** | PROSER2-AS1 | -0.764413299 | 0.00812981 | -0.50466008 | 0.178836219 |
| **High_specific_down** | INE2 | -0.764336679 | 0.005681592 | -0.403263219 | 0.32298676 |

| Category | | High_vs_control | | Low_vs_control | |
|---|---|---|---|---|---|
| *Type* | *gene* | *FC(fold change)* | *FDR* | *FC(fold change)* | *FDR* |
| **High_specific_down** | AC020659.1 | -0.7626075 | 0.000169787 | -0.607633209 | 0.017300584 |
| **High_specific_down** | C6orf47-AS1 | -0.761081895 | 0.000497159 | -0.506414001 | 0.023778366 |
| **High_specific_down** | TENM3-AS1 | -0.760406567 | 0.009016957 | -0.364548839 | 0.388085314 |
| **High_specific_down** | AC018521.2 | -0.758074964 | 4.49E-05 | -0.302476442 | 0.405278048 |
| **High_specific_down** | NUDT4 | -0.756725097 | 3.53E-05 | -0.482623228 | 0.096863064 |
| **High_specific_down** | DHX32 | -0.754090605 | 1.51E-08 | -0.448015158 | 0.072459307 |
| **High_specific_down** | AC009061.1 | -0.754059094 | 0.001733925 | -0.31314982 | 0.493899996 |
| **High_specific_down** | AP003550.1 | -0.753566614 | 0.000168244 | -0.547210254 | 0.035269401 |
| **High_specific_down** | AP4B1-AS1 | -0.752915864 | 2.94E-07 | -0.478034837 | 0.023434502 |
| **High_specific_down** | AL160408.2 | -0.75158621 | 1.54E-10 | -0.427440933 | 0.017079381 |
| **High_specific_down** | AC073333.1 | -0.75008947 | 0.000296599 | -0.409293897 | 0.205455313 |
| **High_specific_down** | MTIF3 | -0.74790764 | 0.002321069 | -0.274284013 | 0.519596205 |
| **High_specific_down** | ACD | -0.745031719 | 0.002000775 | -0.620136696 | 0.066127701 |
| **High_specific_down** | AC011484.1 | -0.743529652 | 0.001728108 | -0.310780225 | 0.449204487 |
| **High_specific_down** | AL162742.1 | -0.742804 | 7.83E-05 | -0.513721433 | 0.030240734 |
| **High_specific_down** | AC010230.1 | -0.742029712 | 0.000587012 | -0.391448335 | 0.183131855 |
| **High_specific_down** | AL162586.1 | -0.740385943 | 0.006400354 | -0.274311221 | 0.545324216 |
| **High_specific_down** | ATP6V0E2-AS1 | -0.738047592 | 1.95E-05 | -0.373868821 | 0.055955007 |
| **High_specific_down** | FO704657.1 | -0.735137863 | 9.79E-08 | -0.402800219 | 0.233907062 |
| **High_specific_down** | ACAP3 | -0.734189151 | 6.72E-06 | -0.363220567 | 0.149677993 |
| **High_specific_down** | AP003068.3 | -0.732928572 | 0.000201172 | -0.605267241 | 0.025979074 |
| **High_specific_down** | EXOC3 | -0.730336649 | 0.000126082 | -0.458323152 | 0.16233381 |
| **High_specific_down** | NUFIP2 | -0.729493723 | 0.006353783 | 0.019293146 | 0.983289738 |
| **High_specific_down** | AC100791.3 | -0.729433322 | 8.59E-07 | -0.395877122 | 0.03437846 |
| **High_specific_down** | AC020931.1 | -0.727093004 | 0.001155878 | -0.32039114 | 0.309077337 |
| **High_specific_down** | AC008760.1 | -0.726334926 | 5.93E-06 | -0.518358567 | 0.092370992 |
| **High_specific_down** | EME1 | -0.72555133 | 1.32E-06 | -0.401068146 | 0.124453823 |
| **High_specific_down** | TRMT112 | -0.72384953 | 0.000127535 | -0.430317768 | 0.092922851 |
| **High_specific_down** | DAAM2-AS1 | -0.722561446 | 0.002299596 | -0.402251973 | 0.316148183 |
| **High_specific_down** | CD24 | -0.721933295 | 0.005012919 | -0.218828016 | 0.519429876 |
| **High_specific_down** | ZNF337-AS1 | -0.721471114 | 2.51E-08 | -0.377611871 | 0.119170821 |
| **High_specific_down** | RBPJL | -0.719704136 | 0.009791058 | -0.124267752 | 0.847583659 |
| **High_specific_down** | HSD17B1 | -0.718745634 | 1.43E-07 | -0.465335302 | 0.025465381 |
| **High_specific_down** | POLR2L | -0.718143157 | 3.24E-05 | -0.302266916 | 0.317206432 |
| **High_specific_down** | SUGP2 | -0.718102621 | 4.29E-06 | -0.409890095 | 0.035172821 |
| **High_specific_down** | AC027319.1 | -0.717562401 | 4.58E-07 | -0.500170354 | 0.019494334 |
| **High_specific_down** | MIEN1 | -0.716864986 | 5.06E-05 | -0.507916717 | 0.031140737 |
| **High_specific_down** | AP000777.3 | -0.715614491 | 4.72E-09 | -0.509438432 | 0.010081087 |
| **High_specific_down** | AC078795.2 | -0.715469016 | 0.006570188 | -0.301899519 | 0.535865926 |
| **High_specific_down** | AC114498.2 | -0.714536675 | 0.009565459 | -0.760567279 | 0.125938689 |
| **High_specific_down** | GPR137 | -0.714508808 | 0.000285225 | -0.450403354 | 0.0332905 |
| **High_specific_down** | ENOSF1 | -0.714475627 | 1.96E-09 | -0.394380766 | 0.047680152 |
| **High_specific_down** | AL356804.1 | -0.71363693 | 0.000124921 | -0.338313708 | 0.180253089 |
| **High_specific_down** | FAAP20 | -0.713611415 | 1.78E-08 | -0.472490078 | 0.01667087 |
| **High_specific_down** | AC087457.1 | -0.713396556 | 0.00170637 | -0.362227941 | 0.318884279 |
| **High_specific_down** | AC002401.1 | -0.713145449 | 0.001519201 | -0.567100908 | 0.071642741 |
| **High_specific_down** | CUL1 | -0.7120913 | 0.001964275 | -0.321706699 | 0.452540134 |
| **High_specific_down** | AC015712.2 | -0.711914368 | 1.18E-05 | -0.338930127 | 0.243651353 |
| **High_specific_down** | SPTY2D1OS | -0.711352856 | 2.58E-06 | -0.498576648 | 0.010876848 |
| **High_specific_down** | ITGB1BP1 | -0.71123294 | 0.000344095 | -0.453553804 | 0.141705538 |
| **High_specific_down** | AC003991.2 | -0.709816477 | 1.54E-06 | -0.477669806 | 0.01740783 |
| **High_specific_down** | SPTAN1 | -0.708516016 | 0.000611527 | -0.546269905 | 0.061033663 |

| Category | | High_vs_control | | Low_vs_control | |
|---|---|---|---|---|---|
| *Type* | *gene* | *FC(fold change)* | *FDR* | *FC(fold change)* | *FDR* |
| **High_specific_down** | RETREG3 | -0.707545685 | 4.51E-06 | -0.342796199 | 0.199807489 |
| **High_specific_down** | AC106820.5 | -0.70657172 | 4.01E-06 | -0.400698279 | 0.01276343 |
| **High_specific_down** | AC004707.1 | -0.706042499 | 5.70E-08 | -0.442039121 | 0.034907568 |
| **High_specific_down** | AC002347.2 | -0.701570261 | 0.000272627 | -0.455876449 | 0.099774246 |
| **High_specific_down** | AP001453.2 | -0.701142135 | 1.34E-06 | -0.278704471 | 0.302669579 |
| **High_specific_down** | AL022328.4 | -0.698884295 | 0.000591622 | -0.375824491 | 0.220920979 |
| **High_specific_down** | SLA2 | -0.69631661 | 2.41E-05 | -0.566242015 | 0.012040088 |
| **High_specific_down** | AL136084.1 | -0.695359638 | 0.004482755 | -0.332961395 | 0.466682863 |
| **High_specific_down** | STK11 | -0.694484589 | 0.00039991 | -0.384534409 | 0.188765262 |
| **High_specific_down** | AC004596.1 | -0.693078936 | 0.000360912 | -0.498331167 | 0.014463435 |
| **High_specific_down** | HCG20 | -0.693068225 | 0.000828303 | -0.194804788 | 0.671159729 |
| **High_specific_down** | GLB1L3 | -0.691072463 | 1.02E-05 | -0.462042567 | 0.023434502 |
| **High_specific_down** | RPAIN | -0.690933025 | 0.008404876 | -0.389430088 | 0.339373623 |
| **High_specific_down** | DOC2A | -0.690506682 | 1.41E-05 | -0.507093316 | 0.021033627 |
| **High_specific_down** | AC072028.1 | -0.690054095 | 0.001044675 | -0.473937033 | 0.135986097 |
| **High_specific_down** | PEX14 | -0.689018475 | 0.000182896 | -0.511671732 | 0.02656821 |
| **High_specific_down** | FKBP14-AS1 | -0.686982312 | 3.64E-06 | -0.380848047 | 0.097287559 |
| **High_specific_down** | GGNBP1 | -0.683978157 | 1.50E-06 | -0.595902644 | 0.01044216 |
| **High_specific_down** | AL512791.1 | -0.683078763 | 8.74E-10 | -0.457948807 | 0.012040088 |
| **High_specific_down** | TUBB | -0.682877462 | 0.002523341 | -0.286764742 | 0.499798898 |
| **High_specific_down** | GPRC5B | -0.681793697 | 0.005184782 | -0.765963965 | 0.011682559 |
| **High_specific_down** | HMGN3-AS1 | -0.681239615 | 3.27E-10 | -0.529709988 | 0.033727139 |
| **High_specific_down** | AL049569.2 | -0.680147211 | 1.25E-08 | -0.535073137 | 0.010193158 |
| **High_specific_down** | ABHD1 | -0.678129832 | 8.62E-05 | -0.541613468 | 0.016248893 |
| **High_specific_down** | PSMB7 | -0.676906953 | 0.001543819 | -0.352674817 | 0.261563546 |
| **High_specific_down** | AC009163.1 | -0.676359426 | 0.000532479 | -0.371117191 | 0.123626655 |
| **High_specific_down** | AC117503.1 | -0.676351334 | 0.001916164 | -0.212833983 | 0.598105845 |
| **High_specific_down** | CACNA1D | -0.674826232 | 0.000841145 | -0.412117023 | 0.124453823 |
| **High_specific_down** | TRAF7 | -0.674518746 | 0.00110554 | -0.192005624 | 0.659886596 |
| **High_specific_down** | TLCD3A | -0.674394348 | 0.000263111 | -0.409093921 | 0.088873296 |
| **High_specific_down** | TAX1BP1 | -0.672579273 | 0.000881971 | -0.20922548 | 0.596457781 |
| **High_specific_down** | KCP | -0.671001157 | 2.09E-06 | -0.417191817 | 0.044779325 |
| **High_specific_down** | AC027277.1 | -0.67007979 | 7.16E-07 | -0.387729906 | 0.140270842 |
| **High_specific_down** | AC092140.2 | -0.668949928 | 8.46E-05 | -0.43298681 | 0.046767083 |
| **High_specific_down** | CRYAB | -0.668749418 | 6.91E-05 | -0.306266153 | 0.247571446 |
| **High_specific_down** | CFAP410 | -0.666786532 | 0.004142249 | -0.537782674 | 0.055938957 |
| **High_specific_down** | AL513190.1 | -0.666512473 | 0.001916164 | -0.455272467 | 0.038731094 |
| **High_specific_down** | AL020993.1 | -0.666418396 | 0.002752217 | -0.39801431 | 0.263095917 |
| **High_specific_down** | BAZ1A | -0.665204119 | 0.000546415 | -0.545215707 | 0.046649568 |
| **High_specific_down** | P2RX5-TAX1BP3 | -0.664817016 | 1.08E-10 | -0.376416333 | 0.049221788 |
| **High_specific_down** | SNX22 | -0.662563615 | 2.53E-11 | -0.340021505 | 0.037220315 |
| **High_specific_down** | H1-10-AS1 | -0.66217734 | 6.24E-07 | -0.269692577 | 0.295381814 |
| **High_specific_down** | AC122108.1 | -0.66167283 | 3.76E-08 | -0.366415419 | 0.111155369 |
| **High_specific_down** | AC073263.1 | -0.659446056 | 1.55E-08 | -0.424463756 | 0.020575705 |
| **High_specific_down** | JMJD8 | -0.658568557 | 3.85E-07 | -0.378281543 | 0.017639854 |
| **High_specific_down** | AL391650.1 | -0.658174542 | 8.94E-06 | -0.435434224 | 0.01667087 |
| **High_specific_down** | SPCS1 | -0.657659049 | 4.87E-06 | -0.387356212 | 0.076821409 |
| **High_specific_down** | PRICKLE2-AS1 | -0.656816355 | 0.001303855 | -0.302291753 | 0.410769937 |
| **High_specific_down** | AC025283.2 | -0.654190981 | 0.002028873 | -0.508423337 | 0.051171157 |
| **High_specific_down** | CCND1 | -0.650535354 | 0.001430511 | -0.479435663 | 0.099774246 |
| **High_specific_down** | AC012073.1 | -0.650209058 | 0.00015615 | -0.64690705 | 0.028341204 |
| **High_specific_down** | GNAS-AS1 | -0.649342265 | 4.79E-09 | -0.311376327 | 0.11610065 |

| Category | | High_vs_control | | Low_vs_control | |
|---|---|---|---|---|---|
| *Type* | *gene* | *FC(fold change)* | *FDR* | *FC(fold change)* | *FDR* |
| **High_specific_down** | PRKCZ | -0.649133235 | 4.76E-05 | -0.525624686 | 0.021810167 |
| **High_specific_down** | AC008655.1 | -0.647968695 | 6.11E-07 | -0.379778714 | 0.060514922 |
| **High_specific_down** | SARNP | -0.647854548 | 0.007262063 | -0.321920901 | 0.470719032 |
| **High_specific_down** | RNF207 | -0.647624342 | 0.000451084 | -0.321938672 | 0.312334069 |
| **High_specific_down** | FARSA-AS1 | -0.647158428 | 8.16E-05 | -0.414190043 | 0.040392108 |
| **High_specific_down** | BRD3 | -0.646589359 | 0.004397598 | -0.603512623 | 0.026421878 |
| **High_specific_down** | AC090061.1 | -0.646151926 | 0.000377335 | -0.467386564 | 0.100425011 |
| **High_specific_down** | AC078777.1 | -0.645725512 | 3.36E-07 | -0.464076183 | 0.010064492 |
| **High_specific_down** | RBBP7 | -0.643587155 | 0.002035425 | -0.448221962 | 0.158984794 |
| **High_specific_down** | AC008453.1 | -0.643405122 | 0.001789729 | -0.441658385 | 0.202586752 |
| **High_specific_down** | PRR19 | -0.642996072 | 0.000360912 | -0.221920762 | 0.446604223 |
| **High_specific_down** | AL034550.1 | -0.641492462 | 0.000738442 | -0.443919768 | 0.098017695 |
| **High_specific_down** | AL121749.2 | -0.640558192 | 0.003742782 | -0.485490421 | 0.112938627 |
| **High_specific_down** | STEAP3-AS1 | -0.640030172 | 7.06E-07 | -0.174675543 | 0.539544262 |
| **High_specific_down** | AC093512.1 | -0.639659922 | 2.65E-05 | -0.44590509 | 0.033729423 |
| **High_specific_down** | AL121845.1 | -0.636290268 | 0.000306353 | -0.204082485 | 0.659886596 |
| **High_specific_down** | AC010536.3 | -0.634792997 | 0.000247013 | -0.144840672 | 0.688066309 |
| **High_specific_down** | SPATA21 | -0.634204681 | 8.87E-09 | -0.408634269 | 0.011002952 |
| **High_specific_down** | HTRA2 | -0.631031894 | 0.000640612 | -0.286888841 | 0.347258235 |
| **High_specific_down** | AC124248.2 | -0.628425561 | 3.62E-05 | -0.539749657 | 0.020413116 |
| **High_specific_down** | CENPT | -0.627971625 | 3.17E-06 | -0.388053955 | 0.020639402 |
| **High_specific_down** | UBE2E1 | -0.627905435 | 0.00476589 | -0.505974113 | 0.109034555 |
| **High_specific_down** | AC008761.1 | -0.627028466 | 4.54E-09 | -0.395990534 | 0.040109738 |
| **High_specific_down** | AC005253.2 | -0.626787009 | 1.03E-05 | -0.325220504 | 0.182577607 |
| **High_specific_down** | RSPH10B | -0.625431455 | 0.001021704 | -0.249196724 | 0.570706732 |
| **High_specific_down** | CNOT10-AS1 | -0.623689937 | 0.007737488 | -0.39901732 | 0.25444223 |
| **High_specific_down** | AC022167.2 | -0.621781936 | 3.83E-08 | -0.337967736 | 0.049652956 |
| **High_specific_down** | AC135050.7 | -0.620796974 | 0.000215763 | -0.31704559 | 0.238690668 |
| **High_specific_down** | AL354813.1 | -0.62074296 | 0.001903981 | -0.210426887 | 0.600035461 |
| **High_specific_down** | FBXO11 | -0.620362091 | 1.30E-05 | -0.351251113 | 0.048045743 |
| **High_specific_down** | SLC7A6OS | -0.619615504 | 6.45E-06 | -0.423415522 | 0.013352283 |
| **High_specific_down** | M6PR | -0.61930383 | 0.001025496 | -0.284634917 | 0.378636498 |
| **High_specific_down** | AL592146.1 | -0.618852076 | 1.71E-06 | -0.395426828 | 0.135113853 |
| **High_specific_down** | AC138430.2 | -0.618777644 | 0.004646906 | -0.438985523 | 0.159083012 |
| **High_specific_down** | C16orf92 | -0.617883212 | 0.000318582 | -0.267141494 | 0.375153156 |
| **High_specific_down** | COL4A2-AS1 | -0.617672858 | 0.000291418 | -0.246734203 | 0.355181178 |
| **High_specific_down** | AL451070.1 | -0.616053231 | 1.19E-05 | -0.335713831 | 0.102166173 |
| **High_specific_down** | TMPO | -0.615841324 | 0.002119782 | -0.276713718 | 0.389302427 |
| **High_specific_down** | AP001972.2 | -0.615270814 | 2.45E-05 | -0.211183117 | 0.59213274 |
| **High_specific_down** | KLF2P2 | -0.615098864 | 0.000247013 | -0.444622985 | 0.078078331 |
| **High_specific_down** | AC006111.1 | -0.612624 | 0.001411687 | -0.157248343 | 0.775448644 |
| **High_specific_down** | SVIL-AS1 | -0.612073782 | 0.000158199 | -0.453636928 | 0.139824377 |
| **High_specific_down** | ATG4D | -0.611619313 | 1.67E-05 | -0.46702933 | 0.022752786 |
| **High_specific_down** | SERPINE3 | -0.610484804 | 5.47E-05 | -0.360164065 | 0.175067176 |
| **High_specific_down** | METTL5 | -0.609886757 | 3.12E-10 | -0.389601049 | 0.029142974 |
| **High_specific_down** | TSPOAP1-AS1 | -0.609791294 | 7.90E-07 | -0.411907252 | 0.012788338 |
| **High_specific_down** | COX11 | -0.609413068 | 1.30E-06 | -0.400303397 | 0.060483187 |
| **High_specific_down** | LIX1-AS1 | -0.609362774 | 0.001647259 | -0.648191595 | 0.01213067 |
| **High_specific_down** | AC012184.1 | -0.608909283 | 0.000157761 | -0.404700415 | 0.029118561 |
| **High_specific_down** | IFT74 | -0.605113439 | 0.000636728 | -0.474199083 | 0.075410887 |
| **High_specific_down** | TSR2 | -0.605104395 | 1.47E-06 | -0.298737099 | 0.191372351 |
| **High_specific_down** | PHYKPL | -0.604862408 | 8.22E-07 | -0.526223695 | 0.032938465 |

| Category | | High_vs_control | | Low_vs_control | |
|---|---|---|---|---|---|
| *Type* | *gene* | *FC(fold change)* | *FDR* | *FC(fold change)* | *FDR* |
| **High_specific_down** | AC010336.3 | -0.604654067 | 8.32E-07 | -0.330468911 | 0.290243896 |
| **High_specific_down** | TAF6 | -0.6044708 | 0.000194859 | -0.484767369 | 0.067770525 |
| **High_specific_down** | AC097382.2 | -0.604309208 | 4.25E-08 | -0.355873798 | 0.036290727 |
| **High_specific_down** | ZNF839 | -0.602590757 | 0.008433186 | -0.389008017 | 0.22245173 |
| **High_specific_down** | PSD | -0.601248454 | 0.005370452 | -0.370406911 | 0.240922291 |
| **High_specific_down** | AC009630.2 | -0.600667361 | 2.46E-06 | -0.364072331 | 0.060483187 |
| **High_specific_down** | PAQR4 | -0.59931968 | 1.50E-06 | -0.380208661 | 0.060188926 |
| **High_specific_down** | NCBP2-AS1 | -0.598531833 | 0.000256257 | -0.354269364 | 0.181475015 |
| **High_specific_down** | PSMC3IP | -0.598469147 | 9.37E-06 | -0.369445002 | 0.08826069 |
| **High_specific_down** | AC013489.3 | -0.596855105 | 2.38E-06 | -0.354816401 | 0.080338666 |
| **High_specific_down** | CHEK2 | -0.596775245 | 0.000767999 | -0.278040791 | 0.405919591 |
| **High_specific_down** | AC089999.1 | -0.596706143 | 0.000177736 | -0.338786587 | 0.166301401 |
| **High_specific_down** | AL157871.1 | -0.596358805 | 0.000130391 | -0.461201866 | 0.018913545 |
| **High_specific_down** | CCDC103 | -0.596104938 | 0.001023577 | -0.375860038 | 0.088384667 |
| **High_specific_down** | AC092794.2 | -0.595551885 | 3.49E-05 | -0.378000432 | 0.062534121 |
| **High_specific_down** | AC068620.2 | -0.595407539 | 7.41E-06 | -0.399389958 | 0.074111027 |
| **High_specific_down** | AC004623.1 | -0.595080685 | 0.000150156 | -0.428125988 | 0.032985473 |
| **High_specific_down** | AC106820.6 | -0.594901558 | 9.37E-05 | -0.324490753 | 0.128139753 |
| **High_specific_down** | TEFM | -0.594185205 | 0.00028254 | -0.48224469 | 0.064312833 |
| **High_specific_down** | LINC01018 | -0.593188973 | 0.000471812 | -0.479965339 | 0.012402119 |
| **High_specific_down** | AC091180.3 | -0.588620191 | 4.59E-07 | -0.362866464 | 0.027189949 |
| **High_specific_down** | EPHA1 | -0.587658495 | 1.12E-07 | -0.343821166 | 0.036054252 |
| **High_specific_down** | EIF2AK4 | -0.587496719 | 0.009525232 | -0.347297235 | 0.342062981 |
| **High_specific_down** | SYVN1 | -0.587465893 | 0.000227511 | -0.273247357 | 0.421723889 |
| **High_specific_down** | NUCB1-AS1 | -0.587134705 | 2.21E-07 | -0.325419632 | 0.075108512 |
| **High_specific_down** | AC013356.2 | -0.586216193 | 2.58E-05 | -0.3852675 | 0.044267788 |
| **High_specific_down** | VPS35L | -0.585424118 | 3.64E-06 | -0.383098629 | 0.011810825 |
| **High_specific_down** | NME6 | -0.584022761 | 0.002315303 | -0.032362971 | 0.973984804 |
| **High_specific_down** | C12orf65 | -0.582481262 | 0.000791567 | -0.330986424 | 0.225655769 |
| **High_specific_down** | PDZD7 | -0.58165548 | 0.00666823 | -0.392713593 | 0.311651881 |
| **High_specific_down** | AGBL5 | -0.578832692 | 1.15E-06 | -0.460094353 | 0.074523851 |
| **High_specific_down** | PIGL | -0.578488177 | 2.77E-05 | -0.453779408 | 0.011487672 |
| **High_specific_down** | AL139095.4 | -0.578208259 | 0.000285225 | -0.437845022 | 0.074523851 |
| **High_specific_down** | AL354892.2 | -0.576922794 | 0.009526473 | -0.219959532 | 0.574330788 |
| **High_specific_down** | SH2D7 | -0.576694551 | 0.00015615 | -0.238136909 | 0.428795214 |
| **High_specific_down** | UQCC2 | -0.576637415 | 2.99E-06 | -0.34636995 | 0.050367102 |
| **High_specific_down** | AL121928.1 | -0.576424269 | 0.000262568 | -0.322924663 | 0.182851058 |
| **High_specific_down** | AL356652.1 | -0.573097741 | 0.000305317 | -0.076464889 | 0.887595729 |
| **High_specific_down** | LINC00337 | -0.572632127 | 0.000196729 | -0.487725769 | 0.010789044 |
| **High_specific_down** | RAD17 | -0.572286099 | 0.000757896 | -0.411516745 | 0.206700819 |
| **High_specific_down** | NKIRAS1 | -0.572214742 | 7.13E-05 | -0.481846903 | 0.022044189 |
| **High_specific_down** | AC010226.1 | -0.570753696 | 1.49E-05 | -0.375661266 | 0.085926906 |
| **High_specific_down** | TBCB | -0.569793183 | 0.009360052 | -0.483377197 | 0.051643987 |
| **High_specific_down** | AC008440.1 | -0.569685542 | 2.32E-07 | -0.365029878 | 0.040431519 |
| **High_specific_down** | AC087276.2 | -0.569565497 | 6.43E-07 | -0.383144821 | 0.043304743 |
| **High_specific_down** | TAF5 | -0.569479592 | 7.28E-06 | -0.39509795 | 0.084743439 |
| **High_specific_down** | AC018693.1 | -0.569472416 | 0.009051563 | -0.482103557 | 0.068719288 |
| **High_specific_down** | MIP | -0.568526715 | 0.002796372 | -0.065306453 | 0.897519645 |
| **High_specific_down** | AC087280.2 | -0.568439183 | 0.00825553 | -0.554530413 | 0.082033505 |
| **High_specific_down** | AL133406.2 | -0.568343524 | 0.003556795 | -0.347202201 | 0.18040423 |
| **High_specific_down** | MIR6126 | -0.568271738 | 0.002087787 | -0.3853833 | 0.153691822 |
| **High_specific_down** | SPAG17 | -0.567976131 | 2.95E-05 | -0.512925863 | 0.02479182 |

| Category | | High_vs_control | | Low_vs_control | |
|---|---|---|---|---|---|
| *Type* | *gene* | *FC(fold change)* | *FDR* | *FC(fold change)* | *FDR* |
| **High_specific_down** | AC005082.2 | -0.567829779 | 3.55E-05 | -0.396276735 | 0.067212121 |
| **High_specific_down** | AC027309.1 | -0.567816294 | 0.006910105 | -0.518510787 | 0.024711888 |
| **High_specific_down** | L3MBTL2-AS1 | -0.567334728 | 1.67E-05 | -0.383313337 | 0.079211794 |
| **High_specific_down** | ASB6 | -0.567184975 | 0.000155556 | -0.365030047 | 0.092370992 |
| **High_specific_down** | SPG11 | -0.5660304 | 0.00142938 | -0.394371176 | 0.10313652 |
| **High_specific_down** | ATF5 | -0.564395452 | 0.000589163 | -0.409123756 | 0.024711888 |
| **High_specific_down** | AC011466.3 | -0.564265505 | 6.29E-05 | -0.238316094 | 0.399669517 |
| **High_specific_down** | AP001793.1 | -0.563864106 | 9.94E-05 | -0.477116107 | 0.040431519 |
| **High_specific_down** | LHFPL5 | -0.563851917 | 1.81E-06 | -0.332206923 | 0.108313017 |
| **High_specific_down** | AC011558.1 | -0.56267615 | 2.90E-09 | -0.435855436 | 0.026421878 |
| **High_specific_down** | SOD2 | -0.562556441 | 0.000582502 | -0.418301662 | 0.084871959 |
| **High_specific_down** | TMED2-DT | -0.562037888 | 0.000411383 | -0.352384523 | 0.044769865 |
| **High_specific_down** | AC021092.1 | -0.561983489 | 0.000397486 | -0.392272384 | 0.099157308 |
| **High_specific_down** | ATP6V1C2 | -0.561573023 | 1.19E-05 | -0.356754486 | 0.071527883 |
| **High_specific_down** | AC093484.3 | -0.561032153 | 0.000205058 | -0.429821378 | 0.011843563 |
| **High_specific_down** | CHMP1B-AS1 | -0.559648622 | 5.93E-06 | -0.397441475 | 0.091816186 |
| **High_specific_down** | SEC61A2 | -0.559179601 | 1.19E-05 | -0.341570309 | 0.104017368 |
| **High_specific_down** | AL031714.1 | -0.558621662 | 5.34E-08 | -0.304823497 | 0.229437972 |
| **High_specific_down** | AC073863.1 | -0.557949121 | 0.001672796 | -0.322001253 | 0.22777345 |
| **High_specific_down** | AC015802.6 | -0.557716799 | 2.57E-07 | -0.356183075 | 0.067100916 |
| **High_specific_down** | RNU4-5P | -0.557687501 | 0.002833673 | -0.398403498 | 0.211949224 |
| **High_specific_down** | AC011446.1 | -0.55733704 | 6.65E-06 | -0.355737192 | 0.099157308 |
| **High_specific_down** | AC073254.1 | -0.555226935 | 0.000146571 | -0.389183172 | 0.02824833 |
| **High_specific_down** | SPDYA | -0.55426124 | 0.009095604 | -0.375612824 | 0.171935652 |
| **High_specific_down** | AC087588.3 | -0.552556665 | 0.000563305 | -0.098683552 | 0.810944592 |
| **High_specific_down** | AC011603.3 | -0.551569829 | 1.42E-06 | -0.312993804 | 0.106471899 |
| **High_specific_down** | KCNK15 | -0.551444291 | 0.004157487 | -0.365801338 | 0.124751981 |
| **High_specific_down** | SMIM15-AS1 | -0.551364326 | 0.000844326 | -0.311438526 | 0.214506812 |
| **High_specific_down** | CCDC159 | -0.551234572 | 0.000255206 | -0.338990629 | 0.149627929 |
| **High_specific_down** | NDUFC1 | -0.549610453 | 0.000252549 | -0.241281658 | 0.356848458 |
| **High_specific_down** | BNIP3P17 | -0.549097084 | 0.001599576 | -0.352082365 | 0.156145735 |
| **High_specific_down** | PLTP | -0.548582259 | 0.001762941 | -0.335624857 | 0.169957704 |
| **High_specific_down** | AC087439.1 | -0.54851716 | 0.004393528 | -0.307174631 | 0.363353664 |
| **High_specific_down** | ARHGEF19-AS1 | -0.547872923 | 0.000879439 | -0.435192765 | 0.044108381 |
| **High_specific_down** | ARL14EPP1 | -0.547368093 | 0.007033623 | -0.297602961 | 0.345375975 |
| **High_specific_down** | AL662797.1 | -0.546459361 | 1.58E-07 | -0.389976062 | 0.010789044 |
| **High_specific_down** | CHRNA3 | -0.545145275 | 0.000905386 | -0.488267829 | 0.020324065 |
| **High_specific_down** | AC125611.3 | -0.544691064 | 4.12E-06 | -0.298607617 | 0.159143039 |
| **High_specific_down** | AC087289.2 | -0.544603539 | 0.000648507 | -0.42607778 | 0.079498927 |
| **High_specific_down** | TSPAN31 | -0.544561873 | 8.59E-07 | -0.390405216 | 0.016646045 |
| **High_specific_down** | AC090607.1 | -0.544356033 | 0.003114412 | -0.244024296 | 0.452536125 |
| **High_specific_down** | STK17A | -0.544355653 | 0.002554038 | -0.563523646 | 0.013844736 |
| **High_specific_down** | AC008119.1 | -0.543463688 | 9.60E-06 | -0.327317105 | 0.073688956 |
| **High_specific_down** | AL671710.1 | -0.542361746 | 0.000339653 | -0.166565356 | 0.609116436 |
| **High_specific_down** | AL080276.2 | -0.541295275 | 0.000196569 | -0.397013646 | 0.121291049 |
| **High_specific_down** | AC120114.2 | -0.539918945 | 6.62E-05 | -0.310379548 | 0.176377636 |
| **High_specific_down** | CYFIP1 | -0.539716293 | 1.99E-05 | -0.420173684 | 0.032204358 |
| **High_specific_down** | AC018754.1 | -0.539346898 | 0.004125092 | -0.166909181 | 0.720963821 |
| **High_specific_down** | DNMBP-AS1 | -0.537945823 | 0.003343621 | -0.30144546 | 0.333356518 |
| **High_specific_down** | AC004696.1 | -0.537573013 | 0.001320093 | -0.384817295 | 0.095072763 |
| **High_specific_down** | TBC1D10B | -0.536999635 | 0.003769391 | -0.495375609 | 0.041828948 |
| **High_specific_down** | HEXB | -0.536363766 | 5.06E-05 | -0.316276805 | 0.16187698 |

| Category | | High_vs_control | | Low_vs_control | |
|---|---|---|---|---|---|
| *Type* | *gene* | *FC(fold change)* | *FDR* | *FC(fold change)* | *FDR* |
| **High_specific_down** | AL008726.1 | -0.536015127 | 9.16E-05 | -0.4169438 | 0.026999253 |
| **High_specific_down** | C1orf54 | -0.535740821 | 0.000255616 | -0.270347137 | 0.225495396 |
| **High_specific_down** | RSPH9 | -0.535335394 | 0.000141353 | -0.391734209 | 0.040644258 |
| **High_specific_down** | AC034102.5 | -0.535000325 | 0.000324741 | -0.301339063 | 0.403796252 |
| **High_specific_down** | AC011731.1 | -0.534548263 | 0.000416819 | -0.490239645 | 0.021313858 |
| **High_specific_down** | DND1 | -0.533789475 | 0.000416922 | -0.455397833 | 0.020451934 |
| **High_specific_down** | AL122035.1 | -0.533178182 | 0.000302008 | -0.286397299 | 0.337480609 |
| **High_specific_down** | LMO7-AS1 | -0.532754132 | 0.000607116 | -0.433025879 | 0.029118561 |
| **High_specific_down** | HDLBP | -0.53249473 | 0.001113181 | -0.160977407 | 0.567612542 |
| **High_specific_down** | AC005786.3 | -0.532322183 | 0.000555261 | -0.381172466 | 0.05346052 |
| **High_specific_down** | ATG7 | -0.531812394 | 0.000213463 | -0.417628855 | 0.067640158 |
| **High_specific_down** | ESRRA | -0.530782373 | 0.00247215 | -0.278094408 | 0.41173555 |
| **High_specific_down** | AC084125.4 | -0.530177774 | 0.000304819 | -0.299777422 | 0.126041143 |
| **High_specific_down** | AC002550.1 | -0.530138242 | 1.47E-06 | -0.419827654 | 0.022309213 |
| **High_specific_down** | CD81-AS1 | -0.529523866 | 0.000463363 | -0.268761578 | 0.187529129 |
| **High_specific_down** | AP001372.3 | -0.529328717 | 0.006680812 | -0.184455256 | 0.675429364 |
| **High_specific_down** | SNHG7 | -0.528468659 | 4.04E-05 | -0.222666195 | 0.337480609 |
| **High_specific_down** | AL354696.2 | -0.528304221 | 0.004586351 | -0.308990296 | 0.277945382 |
| **High_specific_down** | SMAD1-AS1 | -0.527836538 | 0.000456648 | -0.430855872 | 0.015621329 |
| **High_specific_down** | STAB1 | -0.526924051 | 1.88E-06 | -0.249458942 | 0.243039991 |
| **High_specific_down** | PLCG1-AS1 | -0.526915208 | 2.84E-06 | -0.340891021 | 0.053248215 |
| **High_specific_down** | NCOA4 | -0.526530343 | 0.000458711 | -0.603909356 | 0.024711888 |
| **High_specific_down** | NME7 | -0.526129565 | 0.008620931 | -0.368261076 | 0.238690668 |
| **High_specific_down** | TBCE | -0.525107715 | 0.000263111 | -0.351052842 | 0.154009842 |
| **High_specific_down** | C22orf23 | -0.524189767 | 0.000669076 | -0.212823026 | 0.596377081 |
| **High_specific_down** | HEATR4 | -0.523598538 | 5.96E-05 | -0.486577139 | 0.019361119 |
| **High_specific_down** | AP000757.1 | -0.522843401 | 0.000540137 | -0.231792087 | 0.520393508 |
| **High_specific_down** | AP001001.1 | -0.522681589 | 0.003126331 | -0.331465781 | 0.310066892 |
| **High_specific_down** | MIR762HG | -0.521904459 | 0.000475824 | -0.244036505 | 0.32599407 |
| **High_specific_down** | AP005329.1 | -0.521376404 | 0.000369083 | -0.293393567 | 0.172113594 |
| **High_specific_down** | SNX32 | -0.51994223 | 0.000126082 | -0.267792481 | 0.238690668 |
| **High_specific_down** | ETFRF1 | -0.518016107 | 0.000620544 | -0.439490624 | 0.019720907 |
| **High_specific_down** | UHRF1BP1 | -0.517897306 | 0.000216063 | -0.368194528 | 0.082033505 |
| **High_specific_down** | GTF3C2-AS1 | -0.517692505 | 1.53E-05 | -0.29622526 | 0.096325065 |
| **High_specific_down** | SRP14 | -0.517520825 | 0.004046608 | -0.251716687 | 0.383511825 |
| **High_specific_down** | TYMSOS | -0.517154207 | 0.002202644 | -0.239521459 | 0.368801655 |
| **High_specific_down** | AC037459.3 | -0.51714835 | 0.001399578 | -0.355102126 | 0.16266626 |
| **High_specific_down** | PMCH | -0.516658699 | 0.000485634 | -0.381220968 | 0.112018731 |
| **High_specific_down** | AC139530.1 | -0.516372495 | 9.56E-06 | -0.272557464 | 0.144855643 |
| **High_specific_down** | MYG1-AS1 | -0.516257315 | 0.000753351 | -0.355525778 | 0.113153134 |
| **High_specific_down** | IDH3A | -0.516090531 | 0.001527626 | -0.370980006 | 0.129030789 |
| **High_specific_down** | MAP3K15 | -0.514944313 | 6.03E-06 | -0.356992146 | 0.125938689 |
| **High_specific_down** | GEMIN7-AS1 | -0.514482741 | 0.00044158 | -0.275927894 | 0.183970972 |
| **High_specific_down** | AL354956.1 | -0.513881459 | 9.20E-05 | -0.238042569 | 0.261193556 |
| **High_specific_down** | AC005540.1 | -0.513680551 | 0.00058895 | -0.307363608 | 0.113683526 |
| **High_specific_down** | AC006435.2 | -0.51277113 | 0.000209061 | -0.304862493 | 0.199679609 |
| **High_specific_down** | ORAI2 | -0.512084891 | 0.005367329 | -0.515332006 | 0.032012401 |
| **High_specific_down** | AC037459.2 | -0.511348519 | 1.32E-05 | -0.296810604 | 0.106974231 |
| **High_specific_down** | NOP9 | -0.509805455 | 0.009033274 | -0.366101949 | 0.089181035 |
| **High_specific_down** | ENO3 | -0.509730004 | 2.46E-06 | -0.260395201 | 0.182577607 |
| **High_specific_down** | MXD3 | -0.509453833 | 8.74E-06 | -0.286846931 | 0.167035037 |
| **High_specific_down** | PNKP | -0.509174533 | 9.77E-06 | -0.194928457 | 0.474809364 |

| Category | | High_vs_control | | Low_vs_control | |
|---|---|---|---|---|---|
| *Type* | *gene* | *FC(fold change)* | *FDR* | *FC(fold change)* | *FDR* |
| **High_specific_down** | BMP6 | -0.50912545 | 0.000135555 | -0.24852035 | 0.385478667 |
| **High_specific_down** | AC005480.1 | -0.508847382 | 0.005515159 | -0.273825374 | 0.348667262 |
| **High_specific_down** | AC245052.4 | -0.508271839 | 8.45E-06 | -0.292018316 | 0.124453823 |
| **High_specific_down** | ETFDH | -0.508161614 | 0.002179537 | -0.42273224 | 0.097500314 |
| **High_specific_down** | BUD31 | -0.507737347 | 0.002317923 | -0.307809887 | 0.184204783 |
| **High_specific_down** | B3GNT4 | -0.507125308 | 2.99E-06 | -0.333740445 | 0.113451929 |
| **High_specific_down** | AC022098.1 | -0.506895497 | 0.00012597 | -0.300483105 | 0.146648362 |
| **High_specific_down** | AC023055.1 | -0.506483566 | 0.000317076 | -0.27993725 | 0.23994282 |
| **High_specific_down** | AC020978.9 | -0.505890724 | 0.002019556 | -0.363915955 | 0.16912136 |
| **High_specific_down** | AP000648.4 | -0.505020311 | 0.000833863 | -0.259084722 | 0.285371944 |
| **High_specific_down** | CABP7 | -0.504070406 | 0.003206255 | -0.341343986 | 0.364518746 |
| **High_specific_down** | NCMAP-DT | -0.503761613 | 0.003265256 | -0.269332951 | 0.570895875 |
| **High_specific_down** | DNAJC9-AS1 | -0.503735092 | 0.000203536 | -0.409922615 | 0.021209009 |
| **High_specific_down** | AL512770.1 | -0.502629385 | 1.47E-05 | -0.38723112 | 0.113066414 |
| **High_specific_down** | C2 | -0.502251323 | 0.000930356 | -0.238868531 | 0.373774074 |
| **High_specific_down** | LOXL3 | -0.502168404 | 0.00142676 | -0.47181919 | 0.019361119 |
| **High_specific_down** | AC008764.8 | -0.501723123 | 0.005372699 | -0.369856138 | 0.2059685 |
| **High_specific_down** | TMPO-AS1 | -0.501160727 | 0.000385336 | -0.267295455 | 0.236850964 |
| **High_specific_down** | AC069234.3 | -0.500270274 | 0.000107963 | -0.375626867 | 0.032012401 |
| **High_specific_down** | TMEM79 | -0.50016355 | 0.000161814 | -0.169024552 | 0.636749209 |
| **High_specific_down** | ABRAXAS1 | -0.499608327 | 0.000133434 | -0.336053159 | 0.07062179 |
| **High_specific_down** | PLPP1 | -0.499484621 | 0.004614399 | -0.268877593 | 0.470566301 |
| **High_specific_down** | DLGAP4-AS1 | -0.498746508 | 0.001471564 | -0.310786583 | 0.206947007 |
| **High_specific_down** | MRPS9-AS1 | -0.497965987 | 2.58E-05 | -0.355088494 | 0.102166173 |
| **High_specific_down** | AC130456.5 | -0.497895097 | 0.000437441 | -0.384642116 | 0.099531633 |
| **High_specific_down** | MEA1 | -0.497842659 | 5.32E-05 | -0.356365748 | 0.060483187 |
| **High_specific_down** | DCAF13 | -0.49769479 | 0.009834536 | -0.253771381 | 0.503579654 |
| **High_specific_down** | TMEM223 | -0.496921578 | 0.001529259 | -0.554792958 | 0.016182356 |
| **High_specific_down** | PDAP1 | -0.496303414 | 0.001124495 | -0.060254528 | 0.903711793 |
| **High_specific_down** | AP002893.1 | -0.495271588 | 0.001154354 | -0.191932513 | 0.601310667 |
| **High_specific_down** | AC114546.3 | -0.494398856 | 3.83E-05 | -0.37777164 | 0.04057584 |
| **High_specific_down** | PHC1 | -0.493876411 | 0.000157785 | -0.389850765 | 0.016108092 |
| **High_specific_down** | NIFK-AS1 | -0.492058915 | 7.20E-05 | -0.506288827 | 0.016078774 |
| **High_specific_down** | DDB2 | -0.491992712 | 0.007042435 | -0.360643458 | 0.206700819 |
| **High_specific_down** | AC136469.2 | -0.491131564 | 0.001808815 | -0.46736674 | 0.128035235 |
| **High_specific_down** | VRK2 | -0.491043418 | 0.000405674 | -0.423386707 | 0.035172821 |
| **High_specific_down** | AC007620.2 | -0.490971975 | 0.005803897 | -0.345854605 | 0.182460446 |
| **High_specific_down** | AL157392.3 | -0.490401743 | 0.000161227 | -0.336922361 | 0.100507675 |
| **High_specific_down** | AC005104.1 | -0.489550105 | 0.000211597 | -0.243466651 | 0.37223621 |
| **High_specific_down** | KLC3 | -0.489504975 | 0.000101305 | -0.229929416 | 0.255988399 |
| **High_specific_down** | AC005306.1 | -0.489108857 | 6.72E-05 | -0.090317166 | 0.83813189 |
| **High_specific_down** | MIR3661 | -0.487738297 | 0.000452289 | -0.292582107 | 0.14253758 |
| **High_specific_down** | AC022400.7 | -0.486891358 | 2.99E-06 | -0.270923851 | 0.153012698 |
| **High_specific_down** | PCOLCE-AS1 | -0.486633404 | 0.000554221 | -0.324071665 | 0.085000933 |
| **High_specific_down** | ASB16 | -0.485774466 | 0.009258195 | -0.412550103 | 0.066885028 |
| **High_specific_down** | YIPF2 | -0.485633854 | 7.73E-06 | -0.27159195 | 0.164565311 |
| **High_specific_down** | MRAP | -0.484489862 | 0.007416155 | -0.134923939 | 0.74572512 |
| **High_specific_down** | AC097448.1 | -0.482121116 | 5.26E-05 | -0.359595569 | 0.071563482 |
| **High_specific_down** | Z97986.1 | -0.481601861 | 3.49E-05 | -0.290804581 | 0.181475015 |
| **High_specific_down** | SIRT3 | -0.48145858 | 0.003587392 | -0.280412177 | 0.559996948 |
| **High_specific_down** | AC106886.5 | -0.479439662 | 5.90E-06 | -0.347747318 | 0.046843979 |
| **High_specific_down** | AL137186.2 | -0.478790953 | 0.006141955 | -0.293994224 | 0.235947817 |

| Category | | High_vs_control | | Low_vs_control | |
|---|---|---|---|---|---|
| *Type* | *gene* | *FC(fold change)* | *FDR* | *FC(fold change)* | *FDR* |
| **High_specific_down** | AC015674.1 | -0.477769293 | 4.27E-05 | -0.307540211 | 0.118920483 |
| **High_specific_down** | PTOV1-AS2 | -0.477345599 | 0.000137917 | -0.147865079 | 0.636905375 |
| **High_specific_down** | DIS3L | -0.476125781 | 0.000211597 | -0.524306164 | 0.02985885 |
| **High_specific_down** | RNF39 | -0.475617226 | 0.008599401 | -0.296935741 | 0.42925951 |
| **High_specific_down** | AC116913.1 | -0.475191119 | 0.001157928 | -0.185163487 | 0.516695098 |
| **High_specific_down** | DGUOK-AS1 | -0.475022383 | 0.000967838 | -0.309387525 | 0.167002694 |
| **High_specific_down** | AC017083.2 | -0.473772754 | 0.000278261 | -0.396679075 | 0.043427211 |
| **High_specific_down** | NCAPD3 | -0.47298585 | 7.55E-05 | -0.315465702 | 0.116556985 |
| **High_specific_down** | AL354864.1 | -0.472904308 | 0.003712167 | -0.366003943 | 0.103933551 |
| **High_specific_down** | AC005899.8 | -0.472870696 | 0.00060697 | -0.328914598 | 0.128517725 |
| **High_specific_down** | AC005523.2 | -0.471761689 | 5.42E-06 | -0.308297587 | 0.072643763 |
| **High_specific_down** | COG8 | -0.471253641 | 0.000496723 | -0.168224147 | 0.499800062 |
| **High_specific_down** | AL355999.1 | -0.471173925 | 0.004131362 | -0.343331785 | 0.209993546 |
| **High_specific_down** | TSTD3 | -0.471075253 | 0.00267478 | -0.320058519 | 0.144028218 |
| **High_specific_down** | C6orf120 | -0.470756389 | 0.004913132 | -0.516360004 | 0.018781584 |
| **High_specific_down** | KANSL1L | -0.470646813 | 0.003832608 | -0.336409286 | 0.22245173 |
| **High_specific_down** | AP001885.3 | -0.470261683 | 0.000531986 | -0.418277456 | 0.012120168 |
| **High_specific_down** | AC091564.2 | -0.470114282 | 0.002196564 | -0.222021078 | 0.460135842 |
| **High_specific_down** | LCTL | -0.470114242 | 0.0036016 | -0.392647054 | 0.063146986 |
| **High_specific_down** | CCDC157 | -0.469952475 | 0.001053548 | -0.288482802 | 0.128694411 |
| **High_specific_down** | AC092171.2 | -0.469695731 | 0.00011652 | -0.408851828 | 0.011843563 |
| **High_specific_down** | AC078795.1 | -0.468904749 | 0.000387535 | -0.306313967 | 0.202080961 |
| **High_specific_down** | AC010285.3 | -0.468785466 | 0.00111415 | -0.2882178 | 0.322104633 |
| **High_specific_down** | AC018761.2 | -0.468166551 | 0.0002035 | -0.280419859 | 0.25523685 |
| **High_specific_down** | AC011005.4 | -0.467692664 | 0.004120245 | -0.402035167 | 0.124623932 |
| **High_specific_down** | AC114271.1 | -0.465938149 | 0.00059992 | -0.323067526 | 0.080418423 |
| **High_specific_down** | LAMTOR5-AS1 | -0.465868558 | 0.004548364 | -0.230100128 | 0.434076373 |
| **High_specific_down** | ACSBG1 | -0.465196669 | 4.85E-05 | -0.293717347 | 0.122439232 |
| **High_specific_down** | TMEM50B | -0.464329433 | 0.001662843 | -0.267987138 | 0.338140397 |
| **High_specific_down** | SLIRP | -0.463843992 | 0.001052292 | -0.378694673 | 0.116580553 |
| **High_specific_down** | AC107081.3 | -0.463407353 | 0.007573772 | -0.289878082 | 0.311241713 |
| **High_specific_down** | RABEP1 | -0.463355845 | 0.00030566 | -0.287127278 | 0.214625477 |
| **High_specific_down** | CHRNE | -0.463059102 | 0.0022537 | -0.357133858 | 0.144415004 |
| **High_specific_down** | WDR6 | -0.463058851 | 0.006665814 | -0.315455661 | 0.261193556 |
| **High_specific_down** | AP001107.1 | -0.462900545 | 0.000376608 | -0.251834667 | 0.356848458 |
| **High_specific_down** | SYNC | -0.462650469 | 9.02E-05 | -0.284131645 | 0.181475015 |
| **High_specific_down** | CCNK | -0.462398636 | 0.003122309 | -0.26184965 | 0.301330077 |
| **High_specific_down** | PPP1R13B | -0.461489028 | 0.006402461 | -0.139422461 | 0.779385646 |
| **High_specific_down** | OR7E12P | -0.460246263 | 0.002643909 | -0.376682286 | 0.124718478 |
| **High_specific_down** | SAMD11 | -0.459182626 | 0.000665808 | -0.28695411 | 0.162453148 |
| **High_specific_down** | REST | -0.458946212 | 0.005388727 | -0.142599947 | 0.679615113 |
| **High_specific_down** | MIF-AS1 | -0.458459258 | 0.000277088 | -0.248731945 | 0.287101353 |
| **High_specific_down** | ZNF814 | -0.457410941 | 0.000316637 | -0.203394933 | 0.335598941 |
| **High_specific_down** | ICAM3 | -0.457405267 | 0.003832608 | -0.443498776 | 0.141586951 |
| **High_specific_down** | AL161747.2 | -0.456621824 | 1.49E-05 | -0.372097292 | 0.016255865 |
| **High_specific_down** | AL357140.1 | -0.456583076 | 0.006947127 | -0.119108768 | 0.74411784 |
| **High_specific_down** | VTI1B | -0.45603844 | 0.000942463 | -0.436217758 | 0.021313858 |
| **High_specific_down** | ALG1 | -0.456006722 | 0.000472436 | -0.284296953 | 0.251665161 |
| **High_specific_down** | DLEU2 | -0.455674991 | 0.000961924 | -0.472149774 | 0.052407387 |
| **High_specific_down** | AP003396.1 | -0.454944279 | 2.65E-05 | -0.204902758 | 0.355529646 |
| **High_specific_down** | CYSTM1 | -0.454872871 | 0.003282468 | -0.252181109 | 0.453708317 |
| **High_specific_down** | AC020913.1 | -0.454853381 | 0.000622984 | -0.328562169 | 0.106471899 |

| Category | | High_vs_control | | Low_vs_control | |
|---|---|---|---|---|---|
| *Type* | *gene* | *FC(fold change)* | *FDR* | *FC(fold change)* | *FDR* |
| **High_specific_down** | AC093503.1 | -0.454757281 | 1.12E-05 | -0.320322079 | 0.153012698 |
| **High_specific_down** | AP003465.1 | -0.454387472 | 0.001278188 | -0.38748381 | 0.094265671 |
| **High_specific_down** | PPP3CB-AS1 | -0.453780934 | 0.00010762 | -0.377446533 | 0.017809883 |
| **High_specific_down** | COL18A1 | -0.453399903 | 0.007042435 | -0.294758932 | 0.296789627 |
| **High_specific_down** | NUDT17 | -0.452867309 | 0.009301487 | -0.38417447 | 0.20671477 |
| **High_specific_down** | AL359922.2 | -0.451306402 | 0.001874314 | -0.322493384 | 0.199807489 |
| **High_specific_down** | AC008870.3 | -0.450864748 | 0.001215365 | -0.373742759 | 0.072902328 |
| **High_specific_down** | AC010834.1 | -0.449978565 | 0.003178012 | -0.369342726 | 0.073886587 |
| **High_specific_down** | AC007314.1 | -0.449773144 | 0.000617945 | -0.324015554 | 0.098503913 |
| **High_specific_down** | AC007193.3 | -0.448715891 | 0.006769277 | -0.166929072 | 0.632633227 |
| **High_specific_down** | MMACHC | -0.447695135 | 0.000460376 | -0.229585495 | 0.331979892 |
| **High_specific_down** | GSE1 | -0.446775365 | 0.008685713 | -0.339417191 | 0.29847954 |
| **High_specific_down** | PPP2R5D | -0.445575389 | 0.001377661 | -0.275524897 | 0.391531644 |
| **High_specific_down** | SWI5 | -0.445557204 | 0.001920312 | -0.191496842 | 0.533560201 |
| **High_specific_down** | GPSM2 | -0.445453589 | 0.000394943 | -0.301339881 | 0.142355761 |
| **High_specific_down** | AC006369.1 | -0.445396673 | 0.00738678 | -0.504215753 | 0.051249933 |
| **High_specific_down** | SHARPIN | -0.444882057 | 0.009719424 | -0.418230013 | 0.216255649 |
| **High_specific_down** | AL138724.1 | -0.443913988 | 0.000738442 | -0.19294803 | 0.391807537 |
| **High_specific_down** | GPANK1 | -0.442180609 | 0.005189521 | -0.327544031 | 0.131263573 |
| **High_specific_down** | PTOV1-AS1 | -0.441952196 | 0.000762579 | -0.417445623 | 0.028215232 |
| **High_specific_down** | TVP23A | -0.43994981 | 0.003222526 | -0.22042473 | 0.497478402 |
| **High_specific_down** | AC011511.3 | -0.439061057 | 0.000411144 | -0.235347551 | 0.326640226 |
| **High_specific_down** | AC009630.3 | -0.438927988 | 0.000794489 | -0.187790227 | 0.368800612 |
| **High_specific_down** | AC010320.2 | -0.438550004 | 0.000404321 | -0.186310689 | 0.509331577 |
| **High_specific_down** | AC011495.2 | -0.438434308 | 0.005642288 | -0.356310537 | 0.060483187 |
| **High_specific_down** | ZNF276 | -0.438288484 | 0.001129821 | -0.304802904 | 0.091750135 |
| **High_specific_down** | DDTL | -0.438220024 | 0.000617945 | -0.232746034 | 0.360196046 |
| **High_specific_down** | MRPL20-AS1 | -0.438067504 | 0.002202815 | -0.362278092 | 0.11213417 |
| **High_specific_down** | AL049780.1 | -0.437918724 | 0.006657576 | -0.352471203 | 0.306129298 |
| **High_specific_down** | GOLM1 | -0.435619516 | 0.009016957 | -0.334905888 | 0.201747767 |
| **High_specific_down** | STK24 | -0.435567071 | 0.000263192 | -0.224175995 | 0.335636676 |
| **High_specific_down** | RRM1-AS1 | -0.434333054 | 0.002903966 | -0.186752995 | 0.538983326 |
| **High_specific_down** | AC011270.2 | -0.433505123 | 0.009301487 | -0.191637265 | 0.582757442 |
| **High_specific_down** | IPO9-AS1 | -0.433002634 | 0.002275773 | -0.2356858 | 0.298915511 |
| **High_specific_down** | ITFG2 | -0.432950406 | 0.00226207 | -0.266821905 | 0.238690668 |
| **High_specific_down** | CTSA | -0.43285915 | 0.006347764 | -0.278824268 | 0.283864336 |
| **High_specific_down** | CTNS | -0.432604508 | 0.004642178 | -0.24129209 | 0.377595052 |
| **High_specific_down** | AC064836.3 | -0.429958827 | 8.57E-05 | -0.331575854 | 0.235947817 |
| **High_specific_down** | LNX1 | -0.429541275 | 0.009888368 | -0.247679286 | 0.352817794 |
| **High_specific_down** | AL122035.2 | -0.429476199 | 0.001636483 | -0.277776013 | 0.20671477 |
| **High_specific_down** | AL355312.2 | -0.428750516 | 0.002961091 | -0.359585618 | 0.071040333 |
| **High_specific_down** | AC011498.3 | -0.42789532 | 0.001154549 | -0.206960803 | 0.350554846 |
| **High_specific_down** | AC090617.3 | -0.427894904 | 0.001137815 | -0.392730257 | 0.091595192 |
| **High_specific_down** | AP001107.2 | -0.427757205 | 7.73E-05 | -0.25145474 | 0.283694938 |
| **High_specific_down** | TAF10 | -0.427672876 | 0.000153735 | -0.363261115 | 0.022309213 |
| **High_specific_down** | U47924.2 | -0.427299314 | 0.002509026 | -0.31868787 | 0.114487073 |
| **High_specific_down** | AC025262.2 | -0.426796225 | 0.005677329 | -0.299482194 | 0.242822434 |
| **High_specific_down** | AC134772.1 | -0.426753101 | 0.002175999 | -0.203603799 | 0.365074935 |
| **High_specific_down** | C19orf44 | -0.426742252 | 0.000183146 | -0.240282433 | 0.220880027 |
| **High_specific_down** | AC074117.1 | -0.426698392 | 0.002414469 | -0.298051446 | 0.182460446 |
| **High_specific_down** | WRAP53 | -0.426512631 | 0.001496293 | -0.192202556 | 0.455467493 |
| **High_specific_down** | AP002449.1 | -0.426465363 | 0.001889288 | -0.255841754 | 0.296667794 |

| Category | | High_vs_control | | Low_vs_control | |
|---|---|---|---|---|---|
| *Type* | *gene* | *FC(fold change)* | *FDR* | *FC(fold change)* | *FDR* |
| **High_specific_down** | GPS2 | -0.425926776 | 0.000570137 | -0.202124359 | 0.405053702 |
| **High_specific_down** | AL606760.2 | -0.424725341 | 0.002579973 | -0.229108286 | 0.315808638 |
| **High_specific_down** | DNAH11 | -0.424467291 | 0.000349701 | -0.244481178 | 0.254082374 |
| **High_specific_down** | SLC12A6 | -0.423474577 | 0.006824519 | -0.378589275 | 0.119024215 |
| **High_specific_down** | GATC | -0.422695876 | 0.00168606 | -0.256606889 | 0.219515921 |
| **High_specific_down** | PPP1R12B | -0.422625279 | 0.000426584 | -0.17235353 | 0.525870273 |
| **High_specific_down** | AL606534.2 | -0.422577826 | 0.005731532 | -0.279981307 | 0.462255132 |
| **High_specific_down** | AL161452.1 | -0.421497136 | 0.00345291 | -0.209406966 | 0.4568705 |
| **High_specific_down** | ZACN | -0.420177056 | 0.004718672 | -0.267317531 | 0.119244984 |
| **High_specific_down** | AL139423.1 | -0.419081211 | 0.008822346 | -0.235685343 | 0.354016019 |
| **High_specific_down** | AC069287.2 | -0.418733078 | 0.000332235 | -0.266651045 | 0.310251881 |
| **High_specific_down** | NIP7 | -0.418678488 | 0.002453523 | -0.23477438 | 0.312802109 |
| **High_specific_down** | NIT1 | -0.417833928 | 0.000544642 | -0.355552249 | 0.051643987 |
| **High_specific_down** | AL133453.1 | -0.417281058 | 0.007265403 | -0.257590755 | 0.374897211 |
| **High_specific_down** | SNRK-AS1 | -0.416234251 | 0.002330967 | -0.209665734 | 0.424180737 |
| **High_specific_down** | ZBTB11-AS1 | -0.415490299 | 0.006639929 | -0.362029248 | 0.109882126 |
| **High_specific_down** | P2RY11 | -0.414798401 | 0.002837113 | -0.201931201 | 0.422678445 |
| **High_specific_down** | AC008781.1 | -0.414160911 | 0.004559763 | -0.386074985 | 0.095379804 |
| **High_specific_down** | ZNF710-AS1 | -0.412573039 | 0.001849068 | -0.243166143 | 0.387709887 |
| **High_specific_down** | AC109587.1 | -0.412214485 | 0.001479496 | -0.177488516 | 0.551275325 |
| **High_specific_down** | NMT2 | -0.411741846 | 0.002954492 | -0.322317676 | 0.095081512 |
| **High_specific_down** | ACAD8 | -0.410148413 | 0.003363182 | -0.297614462 | 0.190609749 |
| **High_specific_down** | MROH8 | -0.409090149 | 0.008059643 | -0.055113219 | 0.898476571 |
| **High_specific_down** | TREH | -0.40871099 | 0.004564274 | -0.155434246 | 0.588261964 |
| **High_specific_down** | AC012640.4 | -0.408228484 | 0.000752064 | -0.328414051 | 0.074523851 |
| **High_specific_down** | PXN-AS1 | -0.408052666 | 0.003539649 | -0.297594334 | 0.149732387 |
| **High_specific_down** | RNF157-AS1 | -0.407848294 | 0.009301487 | -0.093866654 | 0.841762621 |
| **High_specific_down** | AC145207.3 | -0.406118844 | 0.000159754 | -0.193253957 | 0.485041556 |
| **High_specific_down** | THY1-AS1 | -0.403772834 | 0.005516183 | -0.251358474 | 0.331385133 |
| **High_specific_down** | TTI2 | -0.403620258 | 0.002670491 | -0.2938373 | 0.133604458 |
| **High_specific_down** | EAF1-AS1 | -0.403290364 | 0.006368902 | -0.333121031 | 0.067825907 |
| **High_specific_down** | STUB1 | -0.402472782 | 0.000738442 | -0.296742592 | 0.140390074 |
| **High_specific_down** | EXOSC9 | -0.402248855 | 0.00903065 | -0.32897458 | 0.172797146 |
| **High_specific_down** | COG1 | -0.400267706 | 0.000851702 | -0.292864465 | 0.130377933 |
| **High_specific_down** | AC012676.1 | -0.400176719 | 0.001209506 | -0.176881154 | 0.536006998 |
| **High_specific_down** | AC016727.1 | -0.400091105 | 0.002333696 | -0.27793988 | 0.31492638 |
| **High_specific_down** | AC087164.2 | -0.398863147 | 0.004515126 | -0.336523211 | 0.145210203 |
| **High_specific_down** | PTPRK-AS1 | -0.398560898 | 0.009402082 | -0.201174381 | 0.521145811 |
| **High_specific_down** | POLG | -0.398161678 | 0.001876026 | -0.33540133 | 0.117790229 |
| **High_specific_down** | TRRAP | -0.397333882 | 0.006300752 | -0.269064369 | 0.293443331 |
| **High_specific_down** | AC007938.2 | -0.395769165 | 0.001430762 | -0.251163962 | 0.178878895 |
| **High_specific_down** | TGFB1 | -0.393012938 | 0.003314867 | -0.198353645 | 0.500923696 |
| **High_specific_down** | AC104066.5 | -0.392470398 | 0.006207222 | -0.265240486 | 0.190609749 |
| **High_specific_down** | AC009120.2 | -0.391224988 | 0.002290158 | -0.254689855 | 0.277910386 |
| **High_specific_down** | AC139887.2 | -0.390972823 | 0.002207833 | -0.175176917 | 0.461589837 |
| **High_specific_down** | POLR1C | -0.390442482 | 0.001872447 | -0.263917804 | 0.166301401 |
| **High_specific_down** | EME2 | -0.389223228 | 0.003597227 | -0.261300033 | 0.182864594 |
| **High_specific_down** | CIITA | -0.388917617 | 0.008715564 | -0.172894504 | 0.544704613 |
| **High_specific_down** | NRDE2 | -0.387862156 | 0.001021704 | -0.313369681 | 0.099531633 |
| **High_specific_down** | CNTD1 | -0.385521001 | 0.00260448 | -0.293597664 | 0.206422229 |
| **High_specific_down** | HAS3 | -0.38382059 | 0.001705134 | -0.388088595 | 0.010805775 |
| **High_specific_down** | H2AZ1-DT | -0.383442147 | 0.009194991 | -0.194365178 | 0.327446266 |

| Category | | High_vs_control | | Low_vs_control | |
|---|---|---|---|---|---|
| *Type* | *gene* | *FC(fold change)* | *FDR* | *FC(fold change)* | *FDR* |
| **High_specific_down** | AFAP1-AS1 | -0.383312124 | 0.006534889 | -0.059934816 | 0.882780346 |
| **High_specific_down** | PRPF39 | -0.383198044 | 0.003433094 | -0.297196261 | 0.170521242 |
| **High_specific_down** | AC005726.2 | -0.380879743 | 0.003569453 | -0.125248221 | 0.630185633 |
| **High_specific_down** | AL136988.1 | -0.377874402 | 0.002257887 | -0.098683594 | 0.759069472 |
| **High_specific_down** | AC073593.2 | -0.37673371 | 0.002469702 | -0.030076158 | 0.958450485 |
| **High_specific_down** | DNASE1 | -0.375512885 | 0.001466117 | -0.186721003 | 0.411860195 |
| **High_specific_down** | AC011462.5 | -0.374964327 | 0.00662956 | -0.231195686 | 0.329740425 |
| **High_specific_down** | CEBPZOS | -0.373450015 | 0.001865896 | -0.309720963 | 0.073520164 |
| **High_specific_down** | AC005606.2 | -0.373386622 | 0.006985522 | -0.270799998 | 0.213527715 |
| **High_specific_down** | EMG1 | -0.371907065 | 0.002554038 | -0.27470325 | 0.170091179 |
| **High_specific_down** | MTFR1 | -0.371295145 | 0.007522872 | -0.211842446 | 0.467944453 |
| **High_specific_down** | CASKIN1 | -0.369399666 | 0.002557897 | -0.231186039 | 0.374857796 |
| **High_specific_down** | ZBTB25 | -0.363496412 | 0.00803187 | -0.275958758 | 0.164565311 |
| **High_specific_down** | AC009133.4 | -0.362370683 | 0.003401907 | -0.257723385 | 0.229391528 |
| **High_specific_down** | LENG1 | -0.360016427 | 0.00342802 | -0.127043563 | 0.716390013 |
| **High_specific_down** | WDR38 | -0.359215539 | 0.008736765 | -0.161839462 | 0.509003473 |
| **High_specific_down** | AL139099.1 | -0.358704469 | 0.007343735 | -0.186459819 | 0.383511825 |
| **High_specific_down** | POLR3H | -0.358013249 | 0.00177168 | -0.194744179 | 0.430321178 |
| **High_specific_down** | AC022558.3 | -0.357678095 | 0.00731252 | -0.366237428 | 0.103916145 |
| **High_specific_down** | AC120057.3 | -0.357578352 | 0.003220846 | -0.302164812 | 0.115057414 |
| **High_specific_down** | CATSPER2P1 | -0.355987563 | 0.001630137 | -0.141589061 | 0.517862651 |
| **High_specific_down** | AC004882.3 | -0.349467362 | 0.007366623 | -0.232597433 | 0.331385133 |
| **High_specific_down** | BTD | -0.348597493 | 0.008035805 | -0.104181031 | 0.725507051 |
| **High_specific_down** | AP001267.1 | -0.348570824 | 0.009981749 | -0.221587404 | 0.407308257 |
| **High_specific_down** | AL358115.1 | -0.345168302 | 0.003920573 | -0.127941143 | 0.605080684 |
| **High_specific_down** | B9D1 | -0.34358673 | 0.008876728 | -0.240267497 | 0.305187081 |
| **High_specific_down** | AP003392.3 | -0.340875406 | 0.009765811 | -0.278765386 | 0.205455313 |
| **High_specific_down** | DOP1A | -0.339439853 | 0.008074494 | -0.16614454 | 0.551338114 |
| **High_specific_down** | DCAF1 | -0.336819796 | 0.00825553 | -0.226016888 | 0.304364105 |
| **High_specific_down** | HNRNPU | -0.333354495 | 0.005950106 | -0.376731279 | 0.026623576 |
| **High_specific_down** | CISD3 | -0.332935986 | 0.001903981 | -0.199340874 | 0.494507259 |
| **High_specific_down** | PRMT5-AS1 | -0.320417309 | 0.008996324 | -0.169910093 | 0.505612791 |
| **High_specific_down** | EHMT2-AS1 | -0.320075674 | 0.007638592 | -0.113362927 | 0.639282217 |
| **High_specific_down** | AL590133.2 | -0.315170793 | 0.006974409 | -0.160731175 | 0.582152407 |
| **common_down** | GMFG | -4.285839476 | 7.88E-07 | -3.661535409 | 0.000140598 |
| **common_down** | MTCO1P12 | -3.921159267 | 9.34E-08 | -3.71170698 | 1.86E-05 |
| **common_down** | MTATP6P1 | -3.648440032 | 4.83E-13 | -4.106682466 | 7.31E-14 |
| **common_down** | MTND1P23 | -2.732236652 | 1.45E-18 | -3.268845807 | 1.65E-24 |
| **common_down** | MTND2P28 | -2.306761684 | 0.00526885 | -3.398151496 | 7.31E-05 |
| **common_down** | AGAP2 | -2.066441808 | 4.54E-05 | -1.413260412 | 0.009232861 |
| **common_down** | SLC51B | -1.955897433 | 1.13E-08 | -1.247059646 | 0.000846348 |
| **common_down** | CDHR2 | -1.871426634 | 1.52E-29 | -1.387493587 | 7.96E-07 |
| **common_down** | AC241644.3 | -1.730806627 | 7.19E-12 | -1.044251857 | 0.000287275 |
| **common_down** | SNAI3-AS1 | -1.635852745 | 6.13E-14 | -1.204431319 | 3.18E-06 |
| **common_down** | AC087289.5 | -1.589469558 | 1.02E-12 | -0.737339556 | 0.009749358 |
| **common_down** | AC113189.1 | -1.585458578 | 8.74E-16 | -0.858216979 | 0.000175661 |
| **common_down** | TSPAN18 | -1.558967639 | 8.07E-12 | -0.782859199 | 0.007423917 |
| **common_down** | AC012317.2 | -1.554093205 | 0.000215325 | -1.602374791 | 0.003905727 |
| **common_down** | FKBP15 | -1.5328007 | 8.28E-12 | -0.78376866 | 0.003698842 |
| **common_down** | ZFYVE19 | -1.526155761 | 5.91E-13 | -0.834786161 | 0.005808948 |
| **common_down** | HTR1A | -1.525534295 | 0.009016562 | -1.916821991 | 0.009145343 |
| **common_down** | URGCP | -1.509092522 | 1.31E-05 | -1.096353376 | 0.009145343 |

| Category | | High_vs_control | | Low_vs_control | |
|---|---|---|---|---|---|
| *Type* | *gene* | *FC(fold change)* | *FDR* | *FC(fold change)* | *FDR* |
| **common_down** | MPV17L2 | -1.505329542 | 7.17E-18 | -1.219822828 | 1.21E-06 |
| **common_down** | AC008079.1 | -1.486749104 | 3.11E-13 | -0.892625973 | 5.80E-05 |
| **common_down** | AC007036.5 | -1.43228667 | 6.74E-17 | -1.239507226 | 1.40E-10 |
| **common_down** | IL17D | -1.430066773 | 2.07E-13 | -0.828384629 | 0.000485733 |
| **common_down** | MOCS1 | -1.420466708 | 1.35E-10 | -1.116771141 | 1.04E-05 |
| **common_down** | SLC41A3 | -1.420329053 | 0.000552124 | -1.241830803 | 0.004903094 |
| **common_down** | PKMYT1 | -1.417189939 | 9.96E-18 | -0.9259295 | 8.96E-11 |
| **common_down** | AL159174.1 | -1.37603267 | 0.00462248 | -1.322905307 | 0.006925702 |
| **common_down** | AP006621.4 | -1.359402558 | 1.54E-14 | -0.878606078 | 0.000143996 |
| **common_down** | CIRBP | -1.356736947 | 1.71E-20 | -0.92328636 | 5.31E-07 |
| **common_down** | AC010503.2 | -1.346908596 | 1.08E-17 | -0.882940159 | 0.001568587 |
| **common_down** | MIR3187 | -1.320551312 | 1.96E-16 | -0.725262663 | 0.002511768 |
| **common_down** | SMAP1 | -1.292974622 | 4.58E-07 | -1.268378025 | 9.24E-06 |
| **common_down** | TPSG1 | -1.277149279 | 6.77E-08 | -1.043233062 | 1.91E-05 |
| **common_down** | AL592078.1 | -1.251888104 | 1.27E-08 | -1.48807877 | 1.13E-09 |
| **common_down** | AP003071.3 | -1.246002341 | 8.04E-10 | -0.714875054 | 0.001956806 |
| **common_down** | AC106739.1 | -1.239969716 | 8.85E-05 | -1.249240213 | 0.000764236 |
| **common_down** | AC145207.2 | -1.224120393 | 8.14E-29 | -0.745032929 | 1.72E-07 |
| **common_down** | AL929472.3 | -1.215101342 | 1.32E-12 | -0.786344071 | 0.001066576 |
| **common_down** | THRIL | -1.187164045 | 9.00E-18 | -0.776649581 | 1.53E-05 |
| **common_down** | ZNF771 | -1.17783613 | 1.54E-16 | -0.828104723 | 1.70E-09 |
| **common_down** | SUPT20HL2 | -1.174890115 | 6.39E-05 | -1.196120905 | 0.000288371 |
| **common_down** | GLB1L2 | -1.159623007 | 0.001137815 | -1.115689856 | 0.00649976 |
| **common_down** | POLR2J | -1.137630314 | 2.77E-09 | -0.946868209 | 0.000427961 |
| **common_down** | SLC29A2 | -1.135724302 | 4.12E-05 | -1.009275002 | 0.00088406 |
| **common_down** | AP003032.2 | -1.12407706 | 5.83E-20 | -0.800150178 | 3.01E-08 |
| **common_down** | ERCC1 | -1.121663971 | 1.70E-13 | -0.77281163 | 3.27E-08 |
| **common_down** | MMEL1 | -1.11702885 | 8.64E-11 | -0.738719348 | 5.31E-07 |
| **common_down** | AC135050.1 | -1.111788422 | 2.12E-20 | -0.792958009 | 3.27E-07 |
| **common_down** | SLC46A1 | -1.109914055 | 1.88E-19 | -0.751462429 | 2.13E-05 |
| **common_down** | DZIP1 | -1.099665676 | 2.88E-11 | -0.830302504 | 0.000634422 |
| **common_down** | AC008686.1 | -1.098037091 | 3.77E-19 | -0.714471467 | 2.16E-05 |
| **common_down** | AC119403.1 | -1.082908399 | 2.37E-13 | -0.87278328 | 1.82E-05 |
| **common_down** | AC022966.1 | -1.079687098 | 4.13E-14 | -0.634884369 | 0.000580198 |
| **common_down** | SLCO4A1-AS1 | -1.072513661 | 2.55E-17 | -0.726369873 | 1.95E-05 |
| **common_down** | FGF22 | -1.070981086 | 8.46E-05 | -1.248862296 | 2.13E-05 |
| **common_down** | AC046143.1 | -1.069602146 | 5.91E-12 | -0.605521151 | 0.004125449 |
| **common_down** | AP000763.4 | -1.055006621 | 2.72E-08 | -0.603878502 | 0.004670076 |
| **common_down** | AC139100.1 | -1.044572618 | 3.35E-06 | -0.859282313 | 9.38E-05 |
| **common_down** | AC005841.1 | -1.03736833 | 3.10E-21 | -0.5992792 | 0.000165006 |
| **common_down** | AP006287.3 | -1.034946284 | 3.58E-14 | -0.670160881 | 1.94E-05 |
| **common_down** | CDC42SE1 | -1.033203921 | 1.24E-18 | -0.665378102 | 0.000104787 |
| **common_down** | CGREF1 | -1.029412879 | 2.10E-07 | -0.758782452 | 0.001070996 |
| **common_down** | AC105020.2 | -1.025227642 | 5.23E-18 | -0.708222359 | 1.14E-06 |
| **common_down** | PDXP-DT | -1.02109513 | 3.08E-16 | -0.658664824 | 9.37E-06 |
| **common_down** | NR1I3 | -1.021019795 | 3.71E-15 | -0.617910415 | 0.000168087 |
| **common_down** | AC109322.1 | -1.017949371 | 1.81E-08 | -0.718202174 | 2.36E-05 |
| **common_down** | AC000068.2 | -1.017311997 | 1.18E-17 | -0.575049451 | 0.000226236 |
| **common_down** | SERHL | -1.017009965 | 4.66E-11 | -0.802272925 | 1.51E-10 |
| **common_down** | AC113208.4 | -1.010762261 | 1.44E-09 | -0.805606291 | 1.04E-05 |
| **common_down** | BSCL2 | -1.006675168 | 9.78E-06 | -0.69608841 | 0.001048016 |
| **common_down** | AC021491.2 | -1.00414913 | 0.000344095 | -0.932243602 | 0.008479902 |

| Category | | High_vs_control | | Low_vs_control | |
|---|---|---|---|---|---|
| *Type* | *gene* | *FC(fold change)* | *FDR* | *FC(fold change)* | *FDR* |
| **common_down** | SAMD14 | -0.999151954 | 2.50E-15 | -0.564045105 | 0.000661958 |
| **common_down** | FAM166A | -0.992100985 | 4.06E-21 | -0.638788432 | 4.11E-05 |
| **common_down** | ACO2 | -0.990203382 | 5.89E-08 | -0.698324659 | 7.86E-07 |
| **common_down** | BZW1-AS1 | -0.99004497 | 4.56E-10 | -0.642224734 | 0.000309343 |
| **common_down** | AL807752.3 | -0.989964199 | 4.80E-16 | -0.615824232 | 5.80E-05 |
| **common_down** | AL583722.1 | -0.98054317 | 4.10E-12 | -0.559599453 | 0.007866817 |
| **common_down** | CCNA2 | -0.971987557 | 3.57E-14 | -0.776163147 | 2.16E-05 |
| **common_down** | MED31 | -0.957990098 | 4.59E-06 | -0.892541211 | 0.000423433 |
| **common_down** | PC | -0.955483942 | 7.60E-19 | -0.598790644 | 0.000485733 |
| **common_down** | AL049830.3 | -0.948976059 | 8.70E-09 | -0.719057081 | 0.001657701 |
| **common_down** | AL669831.3 | -0.944933755 | 4.07E-05 | -0.803718932 | 0.0022807 |
| **common_down** | PTPMT1 | -0.943139393 | 1.99E-14 | -0.817259953 | 1.71E-08 |
| **common_down** | ORMDL2 | -0.942919569 | 1.29E-13 | -0.592661931 | 6.83E-05 |
| **common_down** | BLCAP | -0.940830143 | 5.74E-16 | -0.731480841 | 0.000793561 |
| **common_down** | CFAP157 | -0.940226584 | 4.68E-12 | -0.525045979 | 0.005665715 |
| **common_down** | RPS29 | -0.939949018 | 4.24E-11 | -0.580174058 | 0.000301523 |
| **common_down** | INTS8 | -0.934468936 | 9.87E-06 | -0.905391985 | 1.09E-05 |
| **common_down** | TMEM184A | -0.934123763 | 8.94E-13 | -0.786145968 | 1.59E-07 |
| **common_down** | AL357033.4 | -0.933259231 | 3.37E-06 | -0.703423723 | 0.004346867 |
| **common_down** | ELOA-AS1 | -0.932607875 | 9.34E-15 | -0.765996376 | 2.06E-08 |
| **common_down** | ARL10 | -0.930704752 | 7.36E-10 | -0.617462252 | 5.96E-06 |
| **common_down** | BX255925.4 | -0.930148248 | 1.24E-05 | -0.904711274 | 4.89E-05 |
| **common_down** | AC008894.2 | -0.929396154 | 7.92E-18 | -0.671450071 | 2.07E-06 |
| **common_down** | AC027228.2 | -0.927498807 | 6.53E-07 | -0.775872195 | 1.71E-05 |
| **common_down** | AL357079.2 | -0.927037715 | 2.12E-09 | -0.612857179 | 7.83E-05 |
| **common_down** | AC027237.3 | -0.926593298 | 1.15E-10 | -0.682332656 | 3.29E-05 |
| **common_down** | ACAT2 | -0.922424329 | 6.26E-15 | -0.649376052 | 0.001881044 |
| **common_down** | AL355075.3 | -0.920218364 | 1.83E-12 | -0.670245071 | 1.07E-05 |
| **common_down** | TMEM120B | -0.918412998 | 7.76E-16 | -0.629655385 | 8.21E-06 |
| **common_down** | RBM38-AS1 | -0.911180871 | 1.76E-13 | -0.730529099 | 8.91E-05 |
| **common_down** | MFSD11 | -0.908405819 | 3.04E-12 | -0.597563 | 0.000102013 |
| **common_down** | AL135999.2 | -0.905434442 | 1.85E-07 | -0.689909109 | 0.00137726 |
| **common_down** | AC022211.2 | -0.904658294 | 2.53E-11 | -0.67403395 | 3.82E-05 |
| **common_down** | STARD4-AS1 | -0.902886946 | 1.17E-21 | -0.58348688 | 0.000385467 |
| **common_down** | AC011481.2 | -0.899677804 | 6.87E-11 | -0.651737843 | 1.95E-05 |
| **common_down** | AC012173.1 | -0.89947656 | 1.35E-05 | -0.580084385 | 0.000171569 |
| **common_down** | AC090425.3 | -0.89136922 | 1.04E-10 | -0.673516613 | 0.001932934 |
| **common_down** | AC091564.4 | -0.88144004 | 1.81E-06 | -0.719391434 | 0.000354858 |
| **common_down** | C6orf201 | -0.879105775 | 2.76E-15 | -0.551491779 | 0.002008157 |
| **common_down** | LINC01618 | -0.877778431 | 7.82E-09 | -0.644986399 | 0.001969515 |
| **common_down** | AC012615.3 | -0.87474746 | 1.87E-12 | -0.543977749 | 0.000168304 |
| **common_down** | AC092069.1 | -0.872348565 | 6.11E-16 | -0.605789646 | 0.000194968 |
| **common_down** | AC105020.6 | -0.863923166 | 6.60E-12 | -0.473203259 | 0.000916073 |
| **common_down** | CENPP | -0.863128933 | 3.37E-11 | -0.692677994 | 7.68E-06 |
| **common_down** | AL355310.2 | -0.861859897 | 4.06E-12 | -0.454560869 | 0.007675711 |
| **common_down** | TFIP11 | -0.851066343 | 3.88E-13 | -0.57381561 | 9.10E-05 |
| **common_down** | RCC1L | -0.849913337 | 3.77E-06 | -0.572691717 | 0.008479902 |
| **common_down** | CDNF | -0.844299608 | 1.66E-05 | -0.68532331 | 0.000970786 |
| **common_down** | AL139022.1 | -0.835056882 | 2.80E-11 | -0.687608005 | 0.00010031 |
| **common_down** | AC034102.4 | -0.833696962 | 1.07E-10 | -0.545751808 | 0.001363956 |
| **common_down** | SNAP47 | -0.831935279 | 4.09E-13 | -0.499705231 | 0.002100561 |
| **common_down** | AC010883.3 | -0.823276172 | 2.55E-10 | -0.553742066 | 0.001299104 |

| Category | | High_vs_control | | Low_vs_control | |
|---|---|---|---|---|---|
| *Type* | *gene* | *FC(fold change)* | *FDR* | *FC(fold change)* | *FDR* |
| **common_down** | AC011476.3 | -0.822872782 | 7.41E-06 | -0.674659828 | 0.008197266 |
| **common_down** | SLC52A2 | -0.81989547 | 3.63E-06 | -0.623311525 | 0.005548023 |
| **common_down** | FCHSD1 | -0.819785012 | 3.51E-08 | -0.657743121 | 1.70E-05 |
| **common_down** | H2BC15 | -0.819458532 | 8.38E-08 | -0.733017467 | 6.82E-09 |
| **common_down** | AC131953.1 | -0.817638032 | 1.27E-08 | -0.708360306 | 3.92E-05 |
| **common_down** | AC009097.2 | -0.817282189 | 1.25E-06 | -0.572536069 | 0.008479902 |
| **common_down** | SLC51A | -0.814391508 | 4.64E-07 | -0.702879884 | 0.000212351 |
| **common_down** | AC020928.2 | -0.810718396 | 1.96E-09 | -0.678538324 | 0.000226236 |
| **common_down** | AC084024.3 | -0.80436897 | 2.98E-09 | -0.71024667 | 1.04E-05 |
| **common_down** | BTG3-AS1 | -0.803745729 | 0.000165535 | -0.694145037 | 0.005487992 |
| **common_down** | NCAPH2 | -0.794884704 | 4.80E-05 | -0.699161559 | 0.003963742 |
| **common_down** | AC009630.1 | -0.786067839 | 1.11E-11 | -0.569566471 | 0.000580198 |
| **common_down** | UBE2A | -0.783698282 | 5.09E-11 | -0.637840488 | 6.63E-06 |
| **common_down** | AC009902.2 | -0.775973262 | 3.43E-08 | -0.479752475 | 0.003173975 |
| **common_down** | LAMA5 | -0.774439271 | 2.72E-11 | -0.52234711 | 0.000239896 |
| **common_down** | BCL2L12 | -0.771678664 | 1.54E-09 | -0.626380491 | 0.000227859 |
| **common_down** | CIAO3 | -0.768892892 | 0.000364902 | -0.701095678 | 0.002095184 |
| **common_down** | AL355388.2 | -0.768253459 | 6.98E-12 | -0.546494453 | 0.000514895 |
| **common_down** | AC012184.4 | -0.765878911 | 2.79E-10 | -0.569869679 | 0.00024489 |
| **common_down** | INTS11 | -0.760342548 | 0.001177382 | -0.855316867 | 5.91E-05 |
| **common_down** | AC024267.6 | -0.759612995 | 4.80E-09 | -0.598550581 | 2.92E-05 |
| **common_down** | AC145207.6 | -0.758079333 | 8.03E-07 | -0.546786906 | 0.002985606 |
| **common_down** | UBXN11 | -0.755865075 | 4.86E-12 | -0.494460817 | 0.000757706 |
| **common_down** | AC015912.1 | -0.753888209 | 4.40E-05 | -0.561905085 | 0.007893565 |
| **common_down** | CCDC73 | -0.750376235 | 6.43E-15 | -0.516567461 | 0.000477702 |
| **common_down** | BCS1L | -0.750144922 | 3.83E-08 | -0.527990622 | 0.002120286 |
| **common_down** | AC023593.1 | -0.746179806 | 1.58E-08 | -0.517952203 | 8.68E-05 |
| **common_down** | LTO1 | -0.745738945 | 1.81E-06 | -0.687736035 | 7.10E-09 |
| **common_down** | AC018362.2 | -0.738466227 | 4.59E-08 | -0.694671546 | 0.000224367 |
| **common_down** | ADCY3 | -0.735738368 | 2.14E-11 | -0.574897674 | 2.71E-05 |
| **common_down** | AC092849.1 | -0.735738282 | 9.55E-07 | -0.635132919 | 0.002714471 |
| **common_down** | ZNF235 | -0.73388616 | 2.40E-10 | -0.517291602 | 0.002120286 |
| **common_down** | PPT2-EGFL8 | -0.733276406 | 9.94E-11 | -0.485086226 | 0.004927959 |
| **common_down** | PNCK | -0.729312662 | 3.76E-06 | -0.562749651 | 0.001070996 |
| **common_down** | ZRANB2-AS1 | -0.729083043 | 5.30E-11 | -0.570266246 | 0.000169524 |
| **common_down** | AC005329.2 | -0.724567143 | 0.00028322 | -0.625567762 | 0.000238169 |
| **common_down** | PMEL | -0.720324024 | 1.14E-06 | -0.581655571 | 0.000580198 |
| **common_down** | FAM86GP | -0.719465034 | 7.45E-08 | -0.484281814 | 0.002120286 |
| **common_down** | AC135457.1 | -0.712160705 | 2.14E-12 | -0.576659088 | 3.90E-05 |
| **common_down** | RER1 | -0.708951464 | 8.46E-09 | -0.588899216 | 0.000700693 |
| **common_down** | EMSLR | -0.707460453 | 0.000471812 | -0.701052654 | 0.001571503 |
| **common_down** | AL354836.1 | -0.707037483 | 1.97E-05 | -0.437683397 | 0.001828579 |
| **common_down** | AL358333.3 | -0.703816406 | 7.05E-05 | -0.656665175 | 0.001345892 |
| **common_down** | AP000879.1 | -0.699590915 | 2.14E-07 | -0.483610186 | 0.009112632 |
| **common_down** | SARM1 | -0.69780146 | 1.49E-06 | -0.550697707 | 0.005592149 |
| **common_down** | BBS2 | -0.693763898 | 5.79E-08 | -0.619181883 | 0.000238046 |
| **common_down** | AC022167.1 | -0.691100427 | 2.66E-08 | -0.521390971 | 0.000797961 |
| **common_down** | AL590822.2 | -0.688823223 | 4.78E-06 | -0.585901607 | 0.009782131 |
| **common_down** | AC244517.5 | -0.684755905 | 4.67E-08 | -0.458917156 | 0.003899956 |
| **common_down** | KF459542.1 | -0.682939532 | 1.71E-05 | -0.580795742 | 0.009228956 |
| **common_down** | AC017083.3 | -0.681222595 | 2.25E-05 | -0.628851728 | 0.002119546 |
| **common_down** | ARHGAP45 | -0.680537062 | 9.28E-06 | -0.698481853 | 0.001840559 |

| Category | | High_vs_control | | Low_vs_control | |
|---|---|---|---|---|---|
| *Type* | *gene* | *FC(fold change)* | *FDR* | *FC(fold change)* | *FDR* |
| **common_down** | ACTR1A | -0.680255906 | 3.77E-06 | -0.585193075 | 0.001123072 |
| **common_down** | EEF1D | -0.678647963 | 0.000405674 | -0.76725049 | 0.000890143 |
| **common_down** | WDR54 | -0.674994512 | 0.006821332 | -0.654129743 | 0.008479902 |
| **common_down** | TLDC2 | -0.674505278 | 3.67E-07 | -0.531946757 | 0.00253602 |
| **common_down** | AC025259.1 | -0.66992714 | 1.65E-08 | -0.432997234 | 0.00683098 |
| **common_down** | MCM9 | -0.663924184 | 0.000397486 | -0.53090616 | 0.00589352 |
| **common_down** | CD37 | -0.662153051 | 7.80E-10 | -0.405249769 | 0.007675711 |
| **common_down** | AC091607.2 | -0.658867775 | 2.57E-05 | -0.510175574 | 0.004760893 |
| **common_down** | CREB3L4 | -0.658478755 | 0.002192367 | -0.668882505 | 0.003432054 |
| **common_down** | AC025162.1 | -0.656630848 | 0.00147368 | -0.560324506 | 0.002182032 |
| **common_down** | PAXIP1-AS2 | -0.65352725 | 1.82E-07 | -0.516249252 | 0.000644618 |
| **common_down** | ZRANB1 | -0.650396168 | 7.36E-07 | -0.569151009 | 0.00292425 |
| **common_down** | AC020763.4 | -0.650367314 | 1.93E-07 | -0.440456115 | 0.005741166 |
| **common_down** | GDF9 | -0.649321532 | 2.12E-05 | -0.496239925 | 0.006137034 |
| **common_down** | SLC39A9 | -0.635578456 | 3.78E-07 | -0.562622614 | 0.000896183 |
| **common_down** | AC006460.1 | -0.634840622 | 0.001521736 | -0.721849484 | 0.000581202 |
| **common_down** | C9orf43 | -0.63479177 | 0.000662349 | -0.591988564 | 0.000514895 |
| **common_down** | LCORL | -0.631457965 | 3.95E-08 | -0.528578725 | 0.000807244 |
| **common_down** | AC006441.3 | -0.615096547 | 6.57E-09 | -0.477439271 | 0.002484495 |
| **common_down** | NOP14 | -0.614025424 | 0.001668909 | -0.741429721 | 0.00145525 |
| **common_down** | WDR55 | -0.603071821 | 2.76E-05 | -0.506082555 | 0.008615011 |
| **common_down** | AC087190.1 | -0.601682698 | 0.00661866 | -0.589786128 | 0.00725298 |
| **common_down** | AD000671.3 | -0.597668434 | 0.00683956 | -0.619084853 | 0.004125449 |
| **common_down** | AC010973.2 | -0.594412187 | 8.70E-07 | -0.515461006 | 0.000807244 |
| **common_down** | AC069234.2 | -0.579327123 | 7.54E-08 | -0.423069699 | 0.001568587 |
| **common_down** | SRR | -0.569670191 | 9.65E-07 | -0.415858004 | 0.004227943 |
| **common_down** | C12orf73 | -0.569610274 | 1.30E-06 | -0.423808521 | 0.002747008 |
| **common_down** | AC073641.1 | -0.56540493 | 0.000426584 | -0.638667738 | 0.005084731 |
| **common_down** | AP000777.2 | -0.561559372 | 2.43E-06 | -0.411550876 | 0.007733855 |
| **common_down** | STRADB | -0.552218419 | 0.006050303 | -0.612669535 | 0.00435661 |
| **common_down** | IL16 | -0.541352824 | 0.006386107 | -0.62044693 | 0.003967648 |
| **common_down** | AC002094.2 | -0.537598601 | 0.000320796 | -0.585293411 | 0.000485733 |
| **common_down** | TMED1 | -0.529287829 | 0.000685312 | -0.598050358 | 0.009449406 |
| **common_down** | AC015813.5 | -0.516048318 | 4.75E-05 | -0.404129461 | 0.002916524 |
| **common_down** | CIDEB | -0.509276903 | 4.36E-05 | -0.429400385 | 0.007747624 |
| **common_down** | AL162377.3 | -0.467241473 | 0.00016652 | -0.534428912 | 0.000219726 |
| **common_down** | FAM149B1 | -0.430449772 | 0.000286476 | -0.461608771 | 0.001789408 |
| **High_specific_up** | BASP1-AS1 | 6.832486478 | 5.02E-07 | 4.534652676 | 0.129200084 |
| **High_specific_up** | THAP10 | 6.09728243 | 0.000314716 | 5.507598714 | 0.032288697 |
| **High_specific_up** | SP5 | 6.055297537 | 0.00072006 | 5.359331601 | 0.015902085 |
| **High_specific_up** | LIF | 6.050490783 | 0.000351599 | 5.45582702 | 0.012968056 |
| **High_specific_up** | AP000569.1 | 5.97423108 | 0.004428175 | 4.892415563 | 0.040872798 |
| **High_specific_up** | FKBP7 | 5.946911893 | 0.000408826 | 5.067273049 | 0.079509931 |
| **High_specific_up** | OSTCP6 | 5.919723671 | 0.000245262 | 4.712034548 | 0.049891341 |
| **High_specific_up** | AC110995.1 | 5.848586717 | 0.000294024 | 4.34099453 | 0.108313017 |
| **High_specific_up** | AL662844.3 | 5.775443925 | 0.000719316 | 4.771493375 | 0.077157231 |
| **High_specific_up** | AC004687.3 | 5.773767733 | 7.17E-18 | 3.511498044 | 0.022235721 |
| **High_specific_up** | AL121753.1 | 5.737860161 | 0.00127308 | 5.294542931 | 0.045094953 |
| **High_specific_up** | MINAR1 | 5.703355149 | 0.002395396 | 5.5772461 | 0.012773021 |
| **High_specific_up** | RTEL1P1 | 5.461575483 | 0.008961205 | 4.859725226 | 0.039370548 |
| **High_specific_up** | AL137230.1 | 5.434076707 | 0.004672709 | 3.637356419 | 0.281251847 |
| **High_specific_up** | TMEM170B | 5.430933868 | 0.004620075 | 4.150446391 | 0.137659173 |

| Category | | High_vs_control | | Low_vs_control | |
|---|---|---|---|---|---|
| *Type* | *gene* | *FC(fold change)* | *FDR* | *FC(fold change)* | *FDR* |
| **High_specific_up** | HSPB1P2 | 5.390923935 | 0.006305865 | 4.576960741 | 0.074357858 |
| **High_specific_up** | CXCR2 | 5.340885616 | 0.007147093 | 2.790862532 | 0.483849589 |
| **High_specific_up** | NBPF20 | 5.324909704 | 0.004646906 | 0 | 1 |
| **High_specific_up** | AL512358.2 | 5.301826015 | 0.005579649 | 3.262468806 | 0.343379762 |
| **High_specific_up** | AP003392.5 | 5.278968081 | 0.009403218 | 3.619367109 | 0.338430698 |
| **High_specific_up** | PPIAP29 | 5.268977568 | 0.009181798 | 2.731388029 | 0.489993364 |
| **High_specific_up** | MIR4520-2 | 5.24126384 | 0.007822411 | 3.934220166 | 0.238690668 |
| **High_specific_up** | TCHH | 5.238354374 | 0.008708048 | 3.163306332 | 0.405053773 |
| **High_specific_up** | ZNF567 | 5.238350989 | 0.008708048 | 1.966004692 | 0.639282217 |
| **High_specific_up** | ZNF484 | 5.202222112 | 0.009915369 | 3.616948302 | 0.272117786 |
| **High_specific_up** | EIF4A1P11 | 5.117367232 | 0.008822346 | 4.615385344 | 0.109794812 |
| **High_specific_up** | AC098588.1 | 4.914286942 | 1.31E-05 | 0.915885644 | 0.860370712 |
| **High_specific_up** | CFAP97D2 | 4.680915608 | 0.003869638 | 1.801968182 | 0.604595124 |
| **High_specific_up** | AP001922.6 | 4.549145761 | 0.000133434 | 1.416305354 | 0.776500193 |
| **High_specific_up** | AC008751.3 | 4.511469103 | 9.97E-05 | 2.592357721 | 0.344284084 |
| **High_specific_up** | G0S2 | 4.433936674 | 0.000168906 | 3.290931457 | 0.115057414 |
| **High_specific_up** | ADM2 | 4.424641839 | 8.63E-07 | 3.470342503 | 0.015099363 |
| **High_specific_up** | AC010615.2 | 4.342250309 | 0.001037716 | 3.209834358 | 0.189071439 |
| **High_specific_up** | AC106882.1 | 4.305451796 | 0.000714414 | 2.141215876 | 0.575387329 |
| **High_specific_up** | RPL17P34 | 4.292899922 | 0.002805353 | 3.9372861 | 0.011727402 |
| **High_specific_up** | GRM2 | 4.214563035 | 3.75E-05 | 3.267009707 | 0.044108381 |
| **High_specific_up** | MTIF2P1 | 4.125331388 | 0.00696355 | 2.819521579 | 0.373774074 |
| **High_specific_up** | RPL7P8 | 4.111162582 | 0.006721667 | 2.58956372 | 0.427551895 |
| **High_specific_up** | AL359881.3 | 4.039151069 | 0.004541692 | 3.284181203 | 0.105007001 |
| **High_specific_up** | LINC02348 | 4.037189228 | 0.000120414 | 2.576625818 | 0.272117786 |
| **High_specific_up** | SYNPR | 3.979928444 | 0.003923444 | 2.099945465 | 0.560808627 |
| **High_specific_up** | NDUFA3P2 | 3.965858411 | 0.005034452 | 2.345091564 | 0.413122153 |
| **High_specific_up** | HMX1 | 3.952410238 | 0.005683702 | 1.70303049 | 0.711783952 |
| **High_specific_up** | AL354950.2 | 3.929208151 | 0.003556795 | 2.975011457 | 0.277670486 |
| **High_specific_up** | AC243654.3 | 3.830123374 | 0.008708048 | 3.152462522 | 0.176350828 |
| **High_specific_up** | IER3IP1 | 3.802533697 | 5.19E-06 | 1.88675171 | 0.360730239 |
| **High_specific_up** | LAMP5-AS1 | 3.801082045 | 0.000288426 | 3.1132028 | 0.153691822 |
| **High_specific_up** | SPINK13 | 3.789317226 | 0.008340245 | 3.692837346 | 0.030476258 |
| **High_specific_up** | C17orf102 | 3.727608665 | 0.000485634 | 3.01341872 | 0.028047202 |
| **High_specific_up** | ENTPD3 | 3.599168743 | 0.001605529 | 2.533379299 | 0.315795504 |
| **High_specific_up** | TEX52 | 3.586221217 | 0.000449837 | 2.593694504 | 0.273723144 |
| **High_specific_up** | AL138713.1 | 3.564071012 | 0.006808132 | 3.169404235 | 0.159349315 |
| **High_specific_up** | ADGRD1-AS1 | 3.554933808 | 0.000425145 | 1.044881337 | 0.725621233 |
| **High_specific_up** | AC008280.1 | 3.554638965 | 0.006910105 | 2.284939935 | 0.332334307 |
| **High_specific_up** | MTND2P8 | 3.518848507 | 0.004426076 | 2.952865766 | 0.108313017 |
| **High_specific_up** | MT1G | 3.500204599 | 0.001214635 | 2.564247157 | 0.117531157 |
| **High_specific_up** | NWD1 | 3.498992825 | 0.002622026 | 1.218213104 | 0.701987188 |
| **High_specific_up** | LNC-LBCS | 3.481696053 | 0.00064814 | 3.139929835 | 0.011286228 |
| **High_specific_up** | AC008115.3 | 3.46816261 | 0.005359731 | 2.674510636 | 0.335598941 |
| **High_specific_up** | CD48 | 3.46262395 | 6.84E-05 | 1.96754587 | 0.322104633 |
| **High_specific_up** | MIOS | 3.451853505 | 0.006963746 | 1.761426132 | 0.559172993 |
| **High_specific_up** | ALG5 | 3.378495488 | 0.002275773 | 0.502354484 | 0.91787782 |
| **High_specific_up** | DDX19A | 3.339173287 | 0.000555001 | 2.011957364 | 0.395138112 |
| **High_specific_up** | CYP2D7 | 3.335374271 | 0.000124019 | 2.623240995 | 0.029933409 |
| **High_specific_up** | MYO18B-AS1 | 3.319793406 | 0.005969906 | 2.74022666 | 0.170226762 |
| **High_specific_up** | AC068389.1 | 3.251350369 | 0.000771075 | 1.278456853 | 0.653553556 |
| **High_specific_up** | GDF15 | 3.109109434 | 0.003202062 | 2.30847357 | 0.183444278 |

| Category | | High_vs_control | | Low_vs_control | |
|---|---|---|---|---|---|
| *Type* | *gene* | *FC(fold change)* | *FDR* | *FC(fold change)* | *FDR* |
| **High_specific_up** | AP000619.3 | 3.069018179 | 0.004133886 | 2.156947121 | 0.220344935 |
| **High_specific_up** | AC091729.3 | 3.047192846 | 1.92E-05 | 2.409591639 | 0.021466506 |
| **High_specific_up** | CYSRT1 | 3.028029723 | 0.00041941 | 2.485501848 | 0.104246996 |
| **High_specific_up** | AL662907.1 | 3.022381516 | 0.003619662 | 2.175596423 | 0.343379762 |
| **High_specific_up** | AC005086.3 | 3.016768794 | 0.009891695 | 0.182536729 | 0.98203655 |
| **High_specific_up** | AC008074.1 | 2.896400118 | 0.000131519 | 2.083898566 | 0.11798105 |
| **High_specific_up** | SLC25A36P1 | 2.889152516 | 0.0004442 | 2.341838027 | 0.024602634 |
| **High_specific_up** | AL356413.1 | 2.886707346 | 1.53E-05 | 2.443480718 | 0.016029673 |
| **High_specific_up** | AC093620.1 | 2.882221993 | 0.003959903 | 2.419320021 | 0.067640158 |
| **High_specific_up** | AL731566.3 | 2.860936632 | 0.003214055 | 1.91756656 | 0.285535297 |
| **High_specific_up** | SEC22B4P | 2.81504408 | 4.75E-05 | 1.407170566 | 0.463625648 |
| **High_specific_up** | TAS2R2P | 2.792214394 | 0.003618933 | 1.751451023 | 0.261563546 |
| **High_specific_up** | DDIT4 | 2.776761548 | 1.95E-05 | 2.068790666 | 0.029160197 |
| **High_specific_up** | STAC3 | 2.76462898 | 2.24E-09 | 1.743395459 | 0.021466506 |
| **High_specific_up** | AL359643.2 | 2.752054375 | 0.006593384 | 2.373279191 | 0.088103466 |
| **High_specific_up** | ZFP36 | 2.750302186 | 0.001120138 | 1.689085807 | 0.318884279 |
| **High_specific_up** | NTF4 | 2.747272477 | 0.000769551 | 2.586568584 | 0.016182356 |
| **High_specific_up** | AC122129.1 | 2.738986954 | 0.001430762 | 1.958939352 | 0.1425369 |
| **High_specific_up** | JOSD2 | 2.696013543 | 0.00430661 | 2.632654169 | 0.024540608 |
| **High_specific_up** | MSC | 2.6800005 | 0.000818877 | 2.189119292 | 0.052130576 |
| **High_specific_up** | AC100803.3 | 2.679201717 | 0.003218382 | 2.354396414 | 0.124453823 |
| **High_specific_up** | SNIP1 | 2.666746652 | 1.46E-06 | 2.116668721 | 0.017491627 |
| **High_specific_up** | SHISA7 | 2.663451773 | 0.000405213 | 2.344538605 | 0.019678998 |
| **High_specific_up** | CYP27B1 | 2.6625551 | 0.001036545 | 1.213278093 | 0.590601938 |
| **High_specific_up** | XG | 2.64714732 | 0.001527626 | 1.070164954 | 0.621569842 |
| **High_specific_up** | TMEM218 | 2.638073824 | 7.65E-07 | 1.849682067 | 0.096614804 |
| **High_specific_up** | CHAC1 | 2.590219117 | 0.000240815 | 1.334227756 | 0.379746344 |
| **High_specific_up** | GMCL2 | 2.58542999 | 0.0020176 | 2.096806427 | 0.115797254 |
| **High_specific_up** | LINC01843 | 2.570614369 | 0.004841244 | 2.12432341 | 0.176463683 |
| **High_specific_up** | AC092171.1 | 2.52429995 | 0.00035867 | 1.683654212 | 0.183909866 |
| **High_specific_up** | FOXJ1 | 2.501351819 | 7.08E-07 | 0.962571311 | 0.496725356 |
| **High_specific_up** | PGPEP1 | 2.480713179 | 0.008736765 | 2.460483676 | 0.040764821 |
| **High_specific_up** | DCTN6 | 2.466110094 | 0.00294857 | 1.865469451 | 0.105007001 |
| **High_specific_up** | FGF21 | 2.39061784 | 0.001193512 | 1.757124504 | 0.131335146 |
| **High_specific_up** | ACVRL1 | 2.387289023 | 0.006180744 | 2.375523102 | 0.035546253 |
| **High_specific_up** | TTC9B | 2.382903864 | 0.008065274 | 2.627292716 | 0.010193158 |
| **High_specific_up** | SMIM14 | 2.371171318 | 2.54E-06 | 1.598852113 | 0.040164592 |
| **High_specific_up** | AL645939.3 | 2.366180396 | 0.000166472 | 2.058558325 | 0.021599744 |
| **High_specific_up** | ZNF614 | 2.360857973 | 6.09E-05 | 1.491821762 | 0.209498988 |
| **High_specific_up** | ITGA2B | 2.347096195 | 9.60E-05 | 1.561419858 | 0.055471481 |
| **High_specific_up** | WEE2 | 2.33491146 | 0.005159955 | 2.140562869 | 0.11490449 |
| **High_specific_up** | AC006116.5 | 2.329251133 | 0.007392578 | 1.474452242 | 0.554235987 |
| **High_specific_up** | AC108073.2 | 2.325132152 | 0.000530946 | 1.077109253 | 0.402124946 |
| **High_specific_up** | ZNF24 | 2.309708289 | 1.06E-06 | 1.693011875 | 0.024711888 |
| **High_specific_up** | AL158212.4 | 2.29735462 | 0.002175999 | 1.571362178 | 0.270674738 |
| **High_specific_up** | STK38L | 2.283871585 | 0.003018295 | 1.510777887 | 0.312094334 |
| **High_specific_up** | ANKRD53 | 2.281805216 | 0.005543702 | 1.271673116 | 0.440932366 |
| **High_specific_up** | TXNP6 | 2.279767666 | 0.001032232 | 1.911696641 | 0.075734735 |
| **High_specific_up** | TRIB3 | 2.270558584 | 0.001612631 | 1.611841229 | 0.176377636 |
| **High_specific_up** | AC004687.1 | 2.233546786 | 0.003556795 | 0.754129948 | 0.751121439 |
| **High_specific_up** | ARGFX | 2.222313512 | 3.84E-05 | 1.477380099 | 0.163931207 |
| **High_specific_up** | SLC22A11 | 2.213322184 | 2.07E-06 | 0.914435872 | 0.423869796 |

| Category | | High_vs_control | | Low_vs_control | |
|---|---|---|---|---|---|
| *Type* | *gene* | *FC(fold change)* | *FDR* | *FC(fold change)* | *FDR* |
| **High_specific_up** | TACR1 | 2.189809067 | 0.009654094 | 1.125637506 | 0.557093085 |
| **High_specific_up** | NHLRC2 | 2.183089382 | 0.003311109 | 1.283215052 | 0.539529562 |
| **High_specific_up** | LINC02036 | 2.182878969 | 0.004954973 | -0.712795295 | 0.804343092 |
| **High_specific_up** | AL359915.1 | 2.180713879 | 0.001036776 | 1.870950798 | 0.034496727 |
| **High_specific_up** | AC089984.1 | 2.178279936 | 0.00058895 | 1.780709275 | 0.050280388 |
| **High_specific_up** | LRAT | 2.170237859 | 0.004011238 | 1.65034348 | 0.170510286 |
| **High_specific_up** | PABPC1P11 | 2.165923878 | 0.006975113 | 1.838757174 | 0.113702463 |
| **High_specific_up** | AL359076.1 | 2.144447491 | 5.69E-09 | 1.069735782 | 0.133099301 |
| **High_specific_up** | LINC02736 | 2.137260476 | 0.002849495 | 1.660730206 | 0.166301401 |
| **High_specific_up** | AC010538.1 | 2.122775948 | 0.005786832 | 1.690476269 | 0.089846785 |
| **High_specific_up** | AL662844.4 | 2.115270722 | 0.000181147 | 1.829723226 | 0.010204931 |
| **High_specific_up** | TMEM204 | 2.114776692 | 7.31E-05 | 1.498847661 | 0.145269065 |
| **High_specific_up** | SSR4P1 | 2.108571412 | 0.001646235 | 1.202226708 | 0.401779691 |
| **High_specific_up** | ERVW-1 | 2.107087411 | 0.001124495 | 1.400387612 | 0.2059685 |
| **High_specific_up** | AC145285.2 | 2.093634154 | 0.003027432 | 1.014732734 | 0.485905583 |
| **High_specific_up** | SPATA46 | 2.085966189 | 0.000161814 | 1.114783871 | 0.250071326 |
| **High_specific_up** | FAM120B | 2.085550286 | 0.000786713 | 1.766417603 | 0.052391005 |
| **High_specific_up** | AC007991.3 | 2.060213808 | 0.000338933 | 1.396691032 | 0.089846785 |
| **High_specific_up** | CCDC80 | 2.056402674 | 0.000382931 | 0.409401958 | 0.852421816 |
| **High_specific_up** | AC010618.2 | 2.055505989 | 7.29E-05 | 1.340845509 | 0.207848726 |
| **High_specific_up** | AP001527.1 | 2.035364821 | 0.002487186 | 1.271809585 | 0.329136388 |
| **High_specific_up** | C9orf131 | 2.03043641 | 2.56E-05 | 1.468430351 | 0.08826069 |
| **High_specific_up** | PYY2 | 2.026830174 | 0.002943208 | 1.842497419 | 0.092592928 |
| **High_specific_up** | FOXL2 | 2.024601234 | 0.00852719 | 1.682781806 | 0.141265775 |
| **High_specific_up** | FAM3A | 2.024097224 | 0.000716712 | 0.98222941 | 0.436544731 |
| **High_specific_up** | F2RL3 | 1.987752795 | 0.004329819 | 1.771576281 | 0.130864167 |
| **High_specific_up** | SESN2 | 1.983446244 | 0.006450701 | 1.16341444 | 0.385304319 |
| **High_specific_up** | NRP2 | 1.963611358 | 0.001455382 | 1.40608621 | 0.217533731 |
| **High_specific_up** | HCN4 | 1.959383194 | 0.005515159 | 1.339887068 | 0.295326345 |
| **High_specific_up** | SRGAP1 | 1.958357754 | 0.006801293 | 1.8124506 | 0.043426881 |
| **High_specific_up** | AC004233.2 | 1.951291865 | 0.006501153 | 1.140511731 | 0.295889546 |
| **High_specific_up** | NPRL2 | 1.947194426 | 0.006894058 | 1.065800611 | 0.339559178 |
| **High_specific_up** | NEIL2 | 1.943836259 | 8.18E-13 | 0.969721904 | 0.044108381 |
| **High_specific_up** | POU6F2-AS2 | 1.929507322 | 0.001919791 | 1.361060485 | 0.183970972 |
| **High_specific_up** | KCNE2 | 1.90749103 | 0.002275773 | 1.307676562 | 0.212587261 |
| **High_specific_up** | RNF157 | 1.900442506 | 3.56E-07 | 1.236471213 | 0.02464638 |
| **High_specific_up** | ROCR | 1.896772484 | 2.98E-06 | 0.627888702 | 0.552888604 |
| **High_specific_up** | CLIP2 | 1.893326018 | 0.009095604 | 1.773757195 | 0.091595192 |
| **High_specific_up** | JDP2 | 1.871486138 | 2.08E-05 | 1.404996695 | 0.021209009 |
| **High_specific_up** | CHCHD5 | 1.865691516 | 0.008401101 | 1.301980893 | 0.240344959 |
| **High_specific_up** | BRIX1 | 1.859683303 | 0.000468081 | 1.350828348 | 0.073900378 |
| **High_specific_up** | AC090517.5 | 1.851987259 | 0.001188508 | 1.529285265 | 0.08431348 |
| **High_specific_up** | KLHL35 | 1.828110631 | 0.008235637 | 1.627829285 | 0.029358371 |
| **High_specific_up** | AL590666.2 | 1.818557043 | 2.04E-05 | 1.204845715 | 0.036504155 |
| **High_specific_up** | PANK1 | 1.812839697 | 6.09E-06 | 1.28473256 | 0.021313858 |
| **High_specific_up** | AC104984.5 | 1.812809145 | 0.006492181 | 1.527313574 | 0.146448054 |
| **High_specific_up** | AFAP1L1 | 1.805044323 | 0.005754047 | 0.20795711 | 0.931877611 |
| **High_specific_up** | SEMA3B-AS1 | 1.787497259 | 0.000695779 | 1.576160691 | 0.032347961 |
| **High_specific_up** | TMEM9B | 1.783126422 | 0.000107963 | 1.225094609 | 0.088103466 |
| **High_specific_up** | ATXN3 | 1.765109223 | 2.10E-06 | 1.020066367 | 0.08826069 |
| **High_specific_up** | LEP | 1.76510491 | 0.00177168 | 1.429118787 | 0.05276467 |
| **High_specific_up** | HHIP-AS1 | 1.76229426 | 0.000327913 | 0.342394117 | 0.778568336 |

| Category | | High_vs_control | | Low_vs_control | |
|---|---|---|---|---|---|
| *Type* | *gene* | *FC(fold change)* | *FDR* | *FC(fold change)* | *FDR* |
| **High_specific_up** | AC092295.2 | 1.762012628 | 0.000386381 | 1.143562565 | 0.198199256 |
| **High_specific_up** | MFSD4B | 1.75236742 | 0.003556795 | 1.105243954 | 0.194068424 |
| **High_specific_up** | RNASEH2B | 1.738275321 | 0.008600131 | 1.178310845 | 0.356404521 |
| **High_specific_up** | SLC16A6 | 1.730965407 | 2.31E-05 | 1.130484198 | 0.087476502 |
| **High_specific_up** | LINC01138 | 1.72235683 | 0.000332235 | 1.187509988 | 0.170693724 |
| **High_specific_up** | NAPSA | 1.720387783 | 0.00662956 | 1.25183722 | 0.241201343 |
| **High_specific_up** | IL20RB-AS1 | 1.709764409 | 0.000831181 | 1.245269477 | 0.071563482 |
| **High_specific_up** | NOL8 | 1.709714488 | 0.002855648 | 1.316340006 | 0.125671644 |
| **High_specific_up** | GNAI3 | 1.705691347 | 0.004396459 | 0.423513335 | 0.849007487 |
| **High_specific_up** | ANK2-AS1 | 1.698392317 | 0.00068675 | 1.703951426 | 0.028955464 |
| **High_specific_up** | LINC02593 | 1.696373751 | 5.51E-06 | 1.185455589 | 0.038546632 |
| **High_specific_up** | UGDH-AS1 | 1.693372185 | 0.004622333 | 1.196724072 | 0.245983701 |
| **High_specific_up** | AC011481.3 | 1.686127139 | 4.14E-08 | 0.82316785 | 0.104017368 |
| **High_specific_up** | PLEKHA4 | 1.684869311 | 0.000373559 | 1.526401427 | 0.01039154 |
| **High_specific_up** | AP000787.1 | 1.681740667 | 0.000745285 | 1.226768583 | 0.105518671 |
| **High_specific_up** | AC027307.2 | 1.675275957 | 0.001009377 | 1.416087769 | 0.016594401 |
| **High_specific_up** | LINC01391 | 1.672726625 | 0.003074269 | 0.997599558 | 0.413241839 |
| **High_specific_up** | RGS17 | 1.666989414 | 1.65E-05 | 0.97034477 | 0.255199533 |
| **High_specific_up** | ALKBH6 | 1.664883586 | 0.002373221 | 0.680726007 | 0.576775509 |
| **High_specific_up** | SMARCA2 | 1.658286102 | 0.001707847 | 0.742801581 | 0.559786746 |
| **High_specific_up** | LINC01873 | 1.657893941 | 0.004114808 | 1.460692043 | 0.046278972 |
| **High_specific_up** | FXYD7 | 1.656297112 | 0.000587299 | 1.36457517 | 0.041302625 |
| **High_specific_up** | PEX5L-AS1 | 1.653976202 | 0.003311109 | 0.891770479 | 0.497329709 |
| **High_specific_up** | AL138479.2 | 1.641047607 | 0.003468708 | 0.909050776 | 0.421251425 |
| **High_specific_up** | PIP5KL1 | 1.641000429 | 0.000676988 | 1.154702537 | 0.252290867 |
| **High_specific_up** | AC058791.1 | 1.63892235 | 0.001178106 | 1.118998835 | 0.052715611 |
| **High_specific_up** | AL713922.2 | 1.630129062 | 0.006161582 | 1.388144844 | 0.213527715 |
| **High_specific_up** | UBXN8 | 1.623163532 | 0.009843373 | 1.126764123 | 0.293336313 |
| **High_specific_up** | RN7SKP64 | 1.623041382 | 0.009641457 | 0.996688905 | 0.374897211 |
| **High_specific_up** | FAM221B | 1.619646587 | 1.49E-05 | 1.309190524 | 0.010789044 |
| **High_specific_up** | MIS12 | 1.614710558 | 0.001984635 | 0.80761421 | 0.491261094 |
| **High_specific_up** | LINC02576 | 1.6082404 | 0.00051251 | 0.770863168 | 0.400877316 |
| **High_specific_up** | AC012366.1 | 1.594930707 | 0.001288556 | 0.869702734 | 0.214603675 |
| **High_specific_up** | AMDHD2 | 1.578586196 | 0.007284623 | 0.691112191 | 0.623643207 |
| **High_specific_up** | AC016954.1 | 1.574321406 | 0.005867404 | 0.826990353 | 0.476990141 |
| **High_specific_up** | AL139317.5 | 1.573422188 | 1.44E-05 | 0.741996648 | 0.286766162 |
| **High_specific_up** | CCR10 | 1.566641937 | 3.70E-05 | 1.174017059 | 0.024602634 |
| **High_specific_up** | AC000065.1 | 1.545726449 | 0.003714887 | 1.114945569 | 0.163209014 |
| **High_specific_up** | AC007384.1 | 1.545072498 | 0.003714887 | 1.346533281 | 0.013969069 |
| **High_specific_up** | ALG13 | 1.544681299 | 0.005584546 | 0.949687517 | 0.346197826 |
| **High_specific_up** | SPTY2D1 | 1.541403154 | 0.000846022 | 0.695606612 | 0.427935921 |
| **High_specific_up** | BBC3 | 1.540369769 | 3.21E-08 | 1.036518985 | 0.038731094 |
| **High_specific_up** | ZBED3 | 1.530745621 | 2.20E-10 | 0.925138501 | 0.044223635 |
| **High_specific_up** | RPL5P23 | 1.526818193 | 0.008485637 | 0.847138943 | 0.442681289 |
| **High_specific_up** | AL450163.1 | 1.524891662 | 0.009682386 | 1.098372311 | 0.36490522 |
| **High_specific_up** | IL12RB2 | 1.514460161 | 0.001183919 | 1.04427084 | 0.098854471 |
| **High_specific_up** | TP53I3 | 1.512722074 | 0.000934576 | 0.833041606 | 0.325066785 |
| **High_specific_up** | MPL | 1.509792062 | 0.001175154 | 1.025971117 | 0.11212385 |
| **High_specific_up** | GLIDR | 1.502419232 | 0.002127432 | 1.203170521 | 0.108313017 |
| **High_specific_up** | AC093690.1 | 1.500278014 | 0.003279469 | 1.25421483 | 0.15548483 |
| **High_specific_up** | ITGB7 | 1.499903139 | 0.000285387 | 1.208896306 | 0.069338464 |
| **High_specific_up** | AL354855.1 | 1.497490769 | 0.001208756 | 1.22221287 | 0.08985495 |

| Category | | High_vs_control | | Low_vs_control | |
|---|---|---|---|---|---|
| *Type* | *gene* | *FC(fold change)* | *FDR* | *FC(fold change)* | *FDR* |
| **High_specific_up** | SNCA | 1.496641467 | 0.001017705 | 1.258034542 | 0.028367579 |
| **High_specific_up** | AC025774.1 | 1.494037514 | 0.004437808 | 1.160919022 | 0.240608337 |
| **High_specific_up** | RUNX2 | 1.493274265 | 0.003584091 | 0.963097979 | 0.254263288 |
| **High_specific_up** | LINC01409 | 1.488689898 | 0.001044292 | 1.073737049 | 0.159343095 |
| **High_specific_up** | LIPT2 | 1.488646687 | 0.007086617 | 1.452177445 | 0.028955464 |
| **High_specific_up** | CLMP | 1.484723142 | 0.000816468 | 0.824402106 | 0.250692534 |
| **High_specific_up** | ATAT1 | 1.481784828 | 4.51E-07 | 0.764469315 | 0.08826069 |
| **High_specific_up** | PLXNB3 | 1.47713821 | 0.000247013 | -0.130743541 | 0.939661102 |
| **High_specific_up** | S100A1 | 1.476435392 | 3.78E-07 | 0.637291528 | 0.228745462 |
| **High_specific_up** | RIMS4 | 1.474997724 | 0.006226059 | 0.368800402 | 0.772162919 |
| **High_specific_up** | RSPH3 | 1.474100673 | 0.008146136 | 1.485681905 | 0.055100189 |
| **High_specific_up** | KMT2C | 1.473798423 | 0.006974409 | 1.16700848 | 0.098030086 |
| **High_specific_up** | TPPP2 | 1.472987629 | 0.004939055 | 0.745508731 | 0.509331577 |
| **High_specific_up** | ZNF862 | 1.472634749 | 8.04E-05 | 0.434678703 | 0.654157581 |
| **High_specific_up** | LMNTD2-AS1 | 1.462503566 | 2.21E-05 | 1.109697885 | 0.048895127 |
| **High_specific_up** | KRR1P1 | 1.460530642 | 4.93E-05 | 0.816093295 | 0.244297409 |
| **High_specific_up** | BCDIN3D | 1.459837289 | 0.004352866 | 1.049407858 | 0.076142223 |
| **High_specific_up** | HDGFL3 | 1.457826968 | 6.29E-07 | 0.889848648 | 0.043659099 |
| **High_specific_up** | ASAH2B | 1.445887188 | 0.005883122 | 0.290022276 | 0.861537045 |
| **High_specific_up** | CLYBL-AS2 | 1.444641314 | 0.001930368 | 0.576829868 | 0.572526229 |
| **High_specific_up** | CUL5 | 1.436932722 | 0.006658533 | 0.993808285 | 0.318430401 |
| **High_specific_up** | AC023818.1 | 1.433859846 | 0.00226207 | 1.183799855 | 0.123982822 |
| **High_specific_up** | TULP4 | 1.432754249 | 0.003293379 | 0.468305846 | 0.675338181 |
| **High_specific_up** | ARRDC3 | 1.428115832 | 0.00039991 | 0.660995247 | 0.504225485 |
| **High_specific_up** | AL158163.1 | 1.424254381 | 0.004708269 | 1.13997398 | 0.037805249 |
| **High_specific_up** | ARMC9 | 1.422670951 | 6.64E-05 | 0.677888004 | 0.31931113 |
| **High_specific_up** | RGMA | 1.421676556 | 3.64E-06 | 0.707950619 | 0.313149834 |
| **High_specific_up** | TMED10 | 1.415785416 | 0.00018339 | 0.981653375 | 0.159143039 |
| **High_specific_up** | HSPA4L | 1.415566178 | 0.001867692 | 1.2835701 | 0.047680152 |
| **High_specific_up** | MLYCD | 1.413214953 | 0.007841526 | 0.48457105 | 0.689804983 |
| **High_specific_up** | AL391056.2 | 1.410419128 | 0.003569453 | 1.040578527 | 0.036907635 |
| **High_specific_up** | ESYT1 | 1.404825484 | 0.000352776 | 0.884599756 | 0.08826069 |
| **High_specific_up** | ZNF350 | 1.40200343 | 0.000685312 | 1.092595591 | 0.036559209 |
| **High_specific_up** | C8orf88 | 1.400149952 | 0.001154549 | 1.173570172 | 0.038731094 |
| **High_specific_up** | RCE1 | 1.400042177 | 9.87E-06 | 0.737195064 | 0.213301192 |
| **High_specific_up** | AK5 | 1.398426222 | 0.000937151 | 0.675652022 | 0.439947748 |
| **High_specific_up** | PTPN9 | 1.391655889 | 0.003182445 | 0.902770938 | 0.264517905 |
| **High_specific_up** | AC138951.1 | 1.389188726 | 7.73E-06 | 1.293954361 | 0.012190696 |
| **High_specific_up** | AP000769.2 | 1.388480256 | 0.000442826 | 1.125939473 | 0.018930276 |
| **High_specific_up** | INTS6 | 1.376641888 | 1.39E-09 | 0.826712469 | 0.021130456 |
| **High_specific_up** | AL139274.2 | 1.372539953 | 0.008981417 | 1.311037075 | 0.050067971 |
| **High_specific_up** | MIEF1 | 1.371532123 | 0.005382648 | 1.227668238 | 0.108313017 |
| **High_specific_up** | PPM1L | 1.371503925 | 0.00169866 | -0.097873581 | 0.964917907 |
| **High_specific_up** | DPH5 | 1.369571652 | 2.58E-06 | 0.969937849 | 0.010070889 |
| **High_specific_up** | MAVS | 1.36616043 | 7.05E-05 | 0.657316661 | 0.281251847 |
| **High_specific_up** | ARHGDIA | 1.362220663 | 8.58E-11 | 0.859078883 | 0.01597918 |
| **High_specific_up** | MYL6B | 1.357414987 | 6.31E-10 | 0.690745102 | 0.076630643 |
| **High_specific_up** | AL049869.3 | 1.354935876 | 0.000104531 | 0.642259755 | 0.326985557 |
| **High_specific_up** | FLVCR1 | 1.350355028 | 2.66E-08 | 0.709816302 | 0.091322714 |
| **High_specific_up** | AP000866.4 | 1.346484889 | 0.008200792 | 0.348765191 | 0.815765771 |
| **High_specific_up** | OSGEPL1 | 1.336823173 | 0.008526643 | 0.873153048 | 0.257005178 |
| **High_specific_up** | OR5H8 | 1.335831497 | 4.45E-07 | 0.87608223 | 0.012338188 |

| Category | | High_vs_control | | Low_vs_control | |
|---|---|---|---|---|---|
| *Type* | *gene* | *FC(fold change)* | *FDR* | *FC(fold change)* | *FDR* |
| **High_specific_up** | DICER1 | 1.33446887 | 0.007540935 | 1.045915117 | 0.077789375 |
| **High_specific_up** | AC010542.2 | 1.33361897 | 2.13E-05 | 0.462026808 | 0.54159705 |
| **High_specific_up** | AC009095.1 | 1.320627052 | 0.002263091 | 0.924990187 | 0.152621941 |
| **High_specific_up** | CA11 | 1.32024194 | 0.008350181 | 0.631467359 | 0.500511937 |
| **High_specific_up** | SPG7 | 1.315340041 | 5.78E-05 | 0.875859809 | 0.051171157 |
| **High_specific_up** | AL031709.1 | 1.313799359 | 0.004739413 | 0.687930117 | 0.509837718 |
| **High_specific_up** | NUDT8 | 1.312825519 | 4.03E-14 | 0.782548037 | 0.019346215 |
| **High_specific_up** | RN7SKP127 | 1.311924877 | 4.12E-06 | 1.15121198 | 0.020639402 |
| **High_specific_up** | AL162231.1 | 1.311356613 | 0.004646906 | 1.346603081 | 0.017491627 |
| **High_specific_up** | AL136310.1 | 1.308354922 | 0.008653622 | 1.086812121 | 0.131335146 |
| **High_specific_up** | AC018410.1 | 1.307217652 | 0.00666823 | 1.126812123 | 0.206697584 |
| **High_specific_up** | CXXC1 | 1.29741904 | 9.67E-06 | 0.805779083 | 0.109991304 |
| **High_specific_up** | MALAT1 | 1.295771285 | 4.29E-07 | 0.670969036 | 0.128852592 |
| **High_specific_up** | AL139246.3 | 1.294822143 | 0.001867692 | 0.29055841 | 0.810146864 |
| **High_specific_up** | HIF1A-AS3 | 1.293172205 | 0.001036776 | 0.88647045 | 0.152724517 |
| **High_specific_up** | PPP1R16A | 1.290455319 | 4.80E-05 | 0.957586113 | 0.024236318 |
| **High_specific_up** | ZBED3-AS1 | 1.28760881 | 8.26E-08 | 0.785161748 | 0.021471089 |
| **High_specific_up** | SLC2A14 | 1.281995171 | 0.002984083 | 0.738932634 | 0.28865769 |
| **High_specific_up** | AC068587.4 | 1.279542471 | 1.23E-08 | 1.10442501 | 0.011377255 |
| **High_specific_up** | FAM136A | 1.273775643 | 0.001890562 | 0.91293505 | 0.139824377 |
| **High_specific_up** | PSORS1C3 | 1.272642612 | 0.001721253 | 0.587290161 | 0.474019468 |
| **High_specific_up** | ZNF557 | 1.270434932 | 0.009665937 | 0.661588084 | 0.51277778 |
| **High_specific_up** | ARHGEF26 | 1.266126963 | 0.000588999 | 0.82672017 | 0.091595192 |
| **High_specific_up** | SLC34A3 | 1.262141527 | 0.001679038 | 0.592964354 | 0.287370564 |
| **High_specific_up** | REM2 | 1.259980315 | 0.008379042 | 0.155684014 | 0.927090163 |
| **High_specific_up** | RFX7 | 1.257158498 | 0.000166114 | 0.708233376 | 0.218277451 |
| **High_specific_up** | MARS1 | 1.254501832 | 7.67E-09 | 0.478061084 | 0.164158192 |
| **High_specific_up** | LONRF1 | 1.252688118 | 0.00040703 | 0.987240774 | 0.048402093 |
| **High_specific_up** | ZNF175 | 1.252603048 | 0.004064577 | 0.739193446 | 0.335598941 |
| **High_specific_up** | SLC35A4 | 1.250167229 | 0.000369404 | 0.204870578 | 0.864983633 |
| **High_specific_up** | BNIP3P37 | 1.249173584 | 0.005405914 | 1.314144554 | 0.041246507 |
| **High_specific_up** | NBEAL2 | 1.246927738 | 0.000430229 | 0.874534371 | 0.068401133 |
| **High_specific_up** | AC016245.2 | 1.246705325 | 0.001054578 | 0.568076787 | 0.509003473 |
| **High_specific_up** | AC004241.5 | 1.244897575 | 0.00397466 | 0.635313458 | 0.462431492 |
| **High_specific_up** | CCDC78 | 1.235282072 | 1.08E-07 | 0.611070376 | 0.224052077 |
| **High_specific_up** | AC005840.4 | 1.235052418 | 0.005405914 | 0.613008902 | 0.523019846 |
| **High_specific_up** | SWSAP1 | 1.234988816 | 0.002564705 | 0.781305595 | 0.237133551 |
| **High_specific_up** | RNPEPL1 | 1.233534867 | 0.000472436 | 0.809812813 | 0.121291049 |
| **High_specific_up** | AJM1 | 1.230713219 | 2.38E-08 | 0.559560819 | 0.131962885 |
| **High_specific_up** | AC091132.3 | 1.230421361 | 0.004708269 | 1.251887528 | 0.128128732 |
| **High_specific_up** | AC105233.4 | 1.229989798 | 0.000408826 | 1.173311282 | 0.041019192 |
| **High_specific_up** | AL157871.3 | 1.227625669 | 0.006236598 | 0.992410084 | 0.182851058 |
| **High_specific_up** | LINC00514 | 1.226168455 | 0.000352776 | 0.954505403 | 0.029118561 |
| **High_specific_up** | THOC7-AS1 | 1.225776913 | 0.001397038 | 0.869483399 | 0.162572556 |
| **High_specific_up** | AC097376.3 | 1.221582526 | 0.004541692 | 0.550018734 | 0.563196881 |
| **High_specific_up** | AC004877.1 | 1.21494722 | 0.002091386 | 0.440744728 | 0.523230968 |
| **High_specific_up** | FSIP2 | 1.21149706 | 0.001696897 | 0.751023981 | 0.190576945 |
| **High_specific_up** | CSNK2A2 | 1.211239023 | 0.00205867 | 0.826461741 | 0.104017368 |
| **High_specific_up** | EPHX1 | 1.20826901 | 0.000820102 | 0.952716462 | 0.025099607 |
| **High_specific_up** | SNX6P1 | 1.20741877 | 0.000424668 | 0.72604431 | 0.442121742 |
| **High_specific_up** | AC004890.3 | 1.202552408 | 0.001620174 | 0.481530796 | 0.532265573 |
| **High_specific_up** | RAP1AP | 1.194777681 | 0.001385015 | 0.935114194 | 0.052523744 |

| Category | | High_vs_control | | Low_vs_control | |
|---|---|---|---|---|---|
| *Type* | *gene* | *FC(fold change)* | *FDR* | *FC(fold change)* | *FDR* |
| **High_specific_up** | MAPK7 | 1.188937906 | 5.32E-05 | 0.450407957 | 0.526551577 |
| **High_specific_up** | AC022210.1 | 1.18670284 | 1.46E-06 | 0.505702547 | 0.297713602 |
| **High_specific_up** | B3GALT4 | 1.186460437 | 0.007787769 | 0.488977315 | 0.634063067 |
| **High_specific_up** | AC245060.6 | 1.183454347 | 0.001652905 | 1.172975348 | 0.089011346 |
| **High_specific_up** | CTDSPL2 | 1.182389028 | 0.001253332 | 0.64189837 | 0.33507277 |
| **High_specific_up** | AC012645.4 | 1.180045919 | 0.009332959 | 0.760387655 | 0.225495396 |
| **High_specific_up** | NFE2L1 | 1.178813562 | 3.84E-05 | 0.936948928 | 0.018588331 |
| **High_specific_up** | DGKQ | 1.177693786 | 0.008336666 | 0.7231353 | 0.318884279 |
| **High_specific_up** | AL356057.1 | 1.174606581 | 0.00361173 | 0.516320966 | 0.550917778 |
| **High_specific_up** | SMIM5 | 1.17095036 | 2.50E-06 | 0.935860146 | 0.021864848 |
| **High_specific_up** | TTYH1 | 1.167539495 | 0.001098398 | 0.812112289 | 0.198565814 |
| **High_specific_up** | ZFP3 | 1.165737058 | 0.001585379 | 1.006624711 | 0.078790616 |
| **High_specific_up** | KCNIP2 | 1.15838697 | 0.008564994 | 0.855829107 | 0.23363677 |
| **High_specific_up** | AC005358.1 | 1.146322801 | 2.66E-08 | 0.689586373 | 0.047772539 |
| **High_specific_up** | ZNF335 | 1.145265917 | 3.89E-05 | 0.683986302 | 0.13565369 |
| **High_specific_up** | ZNF542P | 1.144824444 | 0.009843373 | 0.873192768 | 0.213646097 |
| **High_specific_up** | BSN | 1.144218626 | 3.49E-05 | 0.693044441 | 0.043695009 |
| **High_specific_up** | AC021424.2 | 1.143432025 | 0.00657582 | 1.11566198 | 0.078693528 |
| **High_specific_up** | GSEC | 1.137387351 | 0.004848988 | -0.170132303 | 0.902701145 |
| **High_specific_up** | AC126177.5 | 1.135045946 | 0.000968615 | 0.878595627 | 0.17380827 |
| **High_specific_up** | ZNF571-AS1 | 1.134943578 | 4.82E-06 | 0.551618937 | 0.120724207 |
| **High_specific_up** | ACAP1 | 1.131839613 | 0.00024407 | 0.933886106 | 0.02074388 |
| **High_specific_up** | AC137932.1 | 1.128041235 | 0.000315598 | 0.779134315 | 0.029688833 |
| **High_specific_up** | AC006449.7 | 1.12273623 | 4.78E-05 | 0.803983554 | 0.154478105 |
| **High_specific_up** | STC2 | 1.119776794 | 0.00226207 | 0.563717429 | 0.396660848 |
| **High_specific_up** | AC092123.1 | 1.11918221 | 0.007983598 | 0.624954084 | 0.442473054 |
| **High_specific_up** | PIANP | 1.119061866 | 0.000672705 | 0.539238197 | 0.43614412 |
| **High_specific_up** | GNB3 | 1.117902965 | 4.17E-06 | 0.525661332 | 0.131335146 |
| **High_specific_up** | TRIP10 | 1.113719628 | 0.001430252 | 0.759077406 | 0.253104845 |
| **High_specific_up** | AC107959.2 | 1.109558115 | 0.000334281 | 0.623255158 | 0.204776632 |
| **High_specific_up** | AL096840.1 | 1.108558177 | 0.002315303 | 0.582688206 | 0.47631287 |
| **High_specific_up** | WNK4 | 1.105731686 | 0.000796209 | 0.707522265 | 0.161199522 |
| **High_specific_up** | CCN2 | 1.099749555 | 0.001620174 | 1.207430499 | 0.013852628 |
| **High_specific_up** | TMEM134 | 1.097387544 | 7.75E-05 | 0.528568087 | 0.352076387 |
| **High_specific_up** | MTND1P9 | 1.097386926 | 0.004153863 | 0.673076586 | 0.242822434 |
| **High_specific_up** | AP005899.1 | 1.093590257 | 0.008184429 | 0.995574569 | 0.02471894 |
| **High_specific_up** | PIGW | 1.092870422 | 0.001941961 | 0.251127952 | 0.776050095 |
| **High_specific_up** | AC018413.1 | 1.083268674 | 1.44E-05 | 0.805354482 | 0.028582782 |
| **High_specific_up** | MORN1 | 1.082562292 | 0.005045854 | 0.860919428 | 0.119244984 |
| **High_specific_up** | NFYC | 1.081565376 | 0.000542724 | 0.779751137 | 0.06790882 |
| **High_specific_up** | AP005265.1 | 1.081514879 | 0.00386923 | 0.870091791 | 0.191029749 |
| **High_specific_up** | ADGRL1 | 1.077615487 | 6.80E-08 | 0.637909764 | 0.035546253 |
| **High_specific_up** | PPP1R27 | 1.076331529 | 0.001165193 | 0.55834416 | 0.38823687 |
| **High_specific_up** | MLLT6 | 1.074053512 | 2.48E-05 | 0.749102641 | 0.034308628 |
| **High_specific_up** | AC138028.1 | 1.070099026 | 0.006400354 | 0.869040555 | 0.108313017 |
| **High_specific_up** | FBXW4 | 1.070094651 | 0.001655996 | 0.913563359 | 0.034496727 |
| **High_specific_up** | BID | 1.069717503 | 7.27E-06 | 0.886818141 | 0.044355586 |
| **High_specific_up** | ZNF789 | 1.069474711 | 0.004351909 | 1.025171235 | 0.017810208 |
| **High_specific_up** | ZNF840P | 1.069432503 | 0.002143561 | 0.007786316 | 0.998809695 |
| **High_specific_up** | SRPK3 | 1.067272957 | 1.53E-06 | 0.463925538 | 0.165172228 |
| **High_specific_up** | MDN1 | 1.062770168 | 0.000469807 | 0.730927846 | 0.084743439 |
| **High_specific_up** | METRN | 1.060851424 | 1.16E-05 | 0.460788076 | 0.249949554 |

| Category | | High_vs_control | | Low_vs_control | |
|---|---|---|---|---|---|
| *Type* | *gene* | *FC(fold change)* | *FDR* | *FC(fold change)* | *FDR* |
| **High_specific_up** | SSTR2 | 1.060822754 | 1.13E-05 | 0.399856108 | 0.434828475 |
| **High_specific_up** | HSD3B7 | 1.059461437 | 0.000881029 | 0.81791421 | 0.061936344 |
| **High_specific_up** | CNPY4 | 1.05790873 | 3.54E-06 | 0.537233349 | 0.183444278 |
| **High_specific_up** | AL050343.2 | 1.05735266 | 5.05E-06 | 0.625205597 | 0.063524728 |
| **High_specific_up** | LINC00910 | 1.050823568 | 0.006482557 | 0.792224947 | 0.172797146 |
| **High_specific_up** | MUC6 | 1.04893138 | 0.00201663 | 0.682168582 | 0.301144699 |
| **High_specific_up** | CCDC183 | 1.042757737 | 0.0067141 | 0.619644774 | 0.276860331 |
| **High_specific_up** | AP001178.3 | 1.040336704 | 0.003088375 | 0.545735418 | 0.449963316 |
| **High_specific_up** | RBM4 | 1.040181736 | 0.000585822 | 0.694981404 | 0.068996865 |
| **High_specific_up** | GABPB1 | 1.039636454 | 2.59E-08 | 0.567627832 | 0.013382289 |
| **High_specific_up** | ATG14 | 1.037257531 | 0.005350197 | 0.521467779 | 0.506895164 |
| **High_specific_up** | VEGFA | 1.030637044 | 0.002532623 | 0.779908189 | 0.130377933 |
| **High_specific_up** | ETF1P2 | 1.028047258 | 0.00039928 | 1.053191624 | 0.021864848 |
| **High_specific_up** | AC073111.1 | 1.021621057 | 4.41E-05 | 0.628421787 | 0.078078331 |
| **High_specific_up** | WDR45BP1 | 1.021279164 | 0.002275773 | 0.794084119 | 0.126029776 |
| **High_specific_up** | SLFNL1 | 1.020296598 | 3.02E-06 | 0.658175098 | 0.026570725 |
| **High_specific_up** | STARD9 | 1.019933656 | 0.009623366 | 0.76726742 | 0.20636235 |
| **High_specific_up** | AC104335.2 | 1.018300147 | 0.002823377 | 0.812833253 | 0.096614804 |
| **High_specific_up** | ZFAND1 | 1.016748752 | 0.002257088 | 0.540403167 | 0.366214234 |
| **High_specific_up** | CCDC24 | 1.016064778 | 9.61E-07 | 0.626521541 | 0.067212121 |
| **High_specific_up** | NUP62 | 1.015829436 | 3.72E-07 | 0.266217461 | 0.476561596 |
| **High_specific_up** | AL356094.1 | 1.013280046 | 0.009407047 | 0.7696019 | 0.181475015 |
| **High_specific_up** | AL158151.1 | 1.010792104 | 0.000737397 | 0.635979362 | 0.176463683 |
| **High_specific_up** | EPM2A | 1.009922443 | 0.008177093 | 0.594286452 | 0.35880408 |
| **High_specific_up** | MZF1 | 1.000976429 | 7.10E-07 | 0.725821945 | 0.03522556 |
| **High_specific_up** | LZTR1 | 0.995281268 | 0.009995947 | 0.549369197 | 0.51664717 |
| **High_specific_up** | VPS9D1-AS1 | 0.994682839 | 0.005367329 | 0.819296741 | 0.147628211 |
| **High_specific_up** | C8orf82 | 0.991324809 | 3.90E-06 | 0.519231143 | 0.169796956 |
| **High_specific_up** | UTP15 | 0.990240604 | 0.001411687 | 0.511028526 | 0.518360256 |
| **High_specific_up** | GNPNAT1 | 0.989064005 | 0.000769551 | 0.815521081 | 0.055106602 |
| **High_specific_up** | H2BC19P | 0.988474114 | 2.58E-05 | 0.555069369 | 0.010735971 |
| **High_specific_up** | PXK | 0.988015228 | 8.91E-06 | 0.725740344 | 0.016248893 |
| **High_specific_up** | HPS3 | 0.986394843 | 0.000421043 | 0.435319909 | 0.438595771 |
| **High_specific_up** | ARHGAP9 | 0.981065638 | 4.33E-06 | 0.534814464 | 0.180003441 |
| **High_specific_up** | ARRDC3-AS1 | 0.978747493 | 0.003973173 | 0.660435126 | 0.224050503 |
| **High_specific_up** | AC103705.1 | 0.975509663 | 0.00013275 | 0.292077235 | 0.613361834 |
| **High_specific_up** | AL355483.2 | 0.97247666 | 0.001045039 | 1.011159404 | 0.031622122 |
| **High_specific_up** | AC010542.4 | 0.970574987 | 0.008824285 | 0.826935969 | 0.082250633 |
| **High_specific_up** | AC011472.1 | 0.96746545 | 0.006665579 | 0.520886313 | 0.22702161 |
| **High_specific_up** | ZNF486 | 0.967154461 | 0.004011238 | 1.083715719 | 0.03053642 |
| **High_specific_up** | SOX2-OT | 0.964123657 | 0.001898682 | 0.46132255 | 0.432482598 |
| **High_specific_up** | DMPK | 0.963022612 | 0.001649434 | 0.59613372 | 0.197559053 |
| **High_specific_up** | HMGCR | 0.959377881 | 0.009179914 | 0.720694912 | 0.181475015 |
| **High_specific_up** | VLDLR | 0.959230419 | 0.003725428 | 0.469641353 | 0.442473054 |
| **High_specific_up** | AC137630.5 | 0.9587692 | 0.004658993 | 0.781718581 | 0.115797254 |
| **High_specific_up** | RORA | 0.95570527 | 0.000184338 | 0.678935467 | 0.089181035 |
| **High_specific_up** | AL021707.9 | 0.955465755 | 0.004832939 | 0.641172106 | 0.331385133 |
| **High_specific_up** | ZNF295-AS1 | 0.953885101 | 5.90E-06 | 0.669024358 | 0.021496521 |
| **High_specific_up** | AL772363.1 | 0.951496767 | 0.001916164 | 0.598601493 | 0.403620447 |
| **High_specific_up** | PARD6G | 0.950909947 | 0.000344129 | 0.752346838 | 0.158034856 |
| **High_specific_up** | ZFHX2 | 0.95058346 | 0.000378962 | 0.723673226 | 0.118920483 |
| **High_specific_up** | AL162253.2 | 0.9496592 | 0.003784733 | 0.633975628 | 0.232049646 |

| Category | | High_vs_control | | Low_vs_control | |
|---|---|---|---|---|---|
| *Type* | *gene* | *FC(fold change)* | *FDR* | *FC(fold change)* | *FDR* |
| **High_specific_up** | TMC4 | 0.945371047 | 9.60E-05 | 0.757456471 | 0.021140819 |
| **High_specific_up** | SH3BP5 | 0.944778548 | 0.005404244 | 0.540481461 | 0.252290867 |
| **High_specific_up** | SLC7A5 | 0.942116854 | 0.00395783 | 0.567219857 | 0.169677104 |
| **High_specific_up** | ZNF324 | 0.941730071 | 0.001984635 | 0.895578582 | 0.010553182 |
| **High_specific_up** | CEBPB | 0.93791116 | 0.005244785 | 0.502761245 | 0.274579668 |
| **High_specific_up** | RAB6B | 0.93461944 | 0.00046834 | 0.831176436 | 0.02374798 |
| **High_specific_up** | TUB | 0.93318988 | 1.92E-05 | 0.394482 | 0.216255649 |
| **High_specific_up** | DENND2B | 0.931269566 | 4.44E-05 | 0.48062475 | 0.331723185 |
| **High_specific_up** | SEC14L2 | 0.930314786 | 2.24E-05 | 0.870513949 | 0.019361119 |
| **High_specific_up** | AC006058.4 | 0.928706964 | 0.000109671 | 0.619175409 | 0.091449022 |
| **High_specific_up** | AC245100.4 | 0.928132281 | 0.00279558 | 0.631489311 | 0.069711734 |
| **High_specific_up** | ADAM11 | 0.927863087 | 0.003314867 | 0.571899435 | 0.193711827 |
| **High_specific_up** | DXO | 0.920253227 | 2.31E-08 | 0.325668542 | 0.464928685 |
| **High_specific_up** | LRRC46 | 0.920173362 | 0.000263903 | 0.716775457 | 0.018684069 |
| **High_specific_up** | AC022137.4 | 0.919738527 | 1.89E-05 | 0.718092517 | 0.020501447 |
| **High_specific_up** | CUL9 | 0.917362359 | 0.001552398 | 0.449170619 | 0.396848036 |
| **High_specific_up** | AL031123.3 | 0.917295719 | 0.00473489 | 0.294692782 | 0.7239624 |
| **High_specific_up** | PELI3 | 0.913288221 | 0.000554474 | 0.568795989 | 0.153691822 |
| **High_specific_up** | COL10A1 | 0.912622423 | 0.000311994 | 0.987203894 | 0.010308597 |
| **High_specific_up** | AC127024.4 | 0.906035263 | 0.009009894 | 0.824856308 | 0.05022144 |
| **High_specific_up** | LINC00645 | 0.905821738 | 0.006947127 | 0.72839412 | 0.312743495 |
| **High_specific_up** | ZSWIM8 | 0.900917135 | 1.67E-06 | 0.450440836 | 0.124453823 |
| **High_specific_up** | PDZD2 | 0.890924538 | 0.002926777 | 0.565403227 | 0.165543434 |
| **High_specific_up** | AC005726.4 | 0.886978821 | 5.70E-05 | 0.522455365 | 0.0627693 |
| **High_specific_up** | KPNA1 | 0.884713472 | 0.000897471 | 0.552428616 | 0.145986398 |
| **High_specific_up** | MYC | 0.884014518 | 0.00278773 | 0.531414529 | 0.29076112 |
| **High_specific_up** | NRL | 0.877786891 | 0.000204297 | 0.657585254 | 0.073311068 |
| **High_specific_up** | AL353748.2 | 0.872621325 | 0.008824285 | 0.57892877 | 0.381469801 |
| **High_specific_up** | AL355075.2 | 0.871212882 | 0.001532269 | 0.498422667 | 0.186960001 |
| **High_specific_up** | CLDN20 | 0.870789706 | 0.003289941 | 0.765149132 | 0.098094715 |
| **High_specific_up** | AC008105.3 | 0.869927995 | 0.004913691 | 0.691850383 | 0.1683926 |
| **High_specific_up** | VSIG10 | 0.865605237 | 2.51E-05 | 0.245728625 | 0.63933039 |
| **High_specific_up** | AC008581.1 | 0.864583647 | 4.19E-09 | 0.448752217 | 0.228800679 |
| **High_specific_up** | WDR4 | 0.862266668 | 0.00524008 | 0.593140562 | 0.269175329 |
| **High_specific_up** | RASGRP2 | 0.859412186 | 0.006982074 | 0.777873106 | 0.025643345 |
| **High_specific_up** | TFPI | 0.857203208 | 0.000304819 | 0.33842242 | 0.475979273 |
| **High_specific_up** | FMNL1 | 0.855690094 | 1.70E-05 | 0.52244081 | 0.034252409 |
| **High_specific_up** | RYR3 | 0.855282688 | 2.63E-08 | 0.534181834 | 0.011760078 |
| **High_specific_up** | ICA1 | 0.854754954 | 0.006746199 | 0.634331642 | 0.142441206 |
| **High_specific_up** | ALDH3B1 | 0.847723996 | 8.46E-05 | 0.234288373 | 0.571216549 |
| **High_specific_up** | RDH13 | 0.845517855 | 0.000473799 | 0.521785324 | 0.059316205 |
| **High_specific_up** | SLC35A3 | 0.844852025 | 0.002298764 | 0.432734883 | 0.386171843 |
| **High_specific_up** | SPDYE1 | 0.844206761 | 0.001466117 | 0.436812229 | 0.296864531 |
| **High_specific_up** | AC007463.1 | 0.842916338 | 0.002918598 | 0.508122499 | 0.073688956 |
| **High_specific_up** | AC135279.2 | 0.837524529 | 0.007726833 | 0.425273118 | 0.422659107 |
| **High_specific_up** | FXR1 | 0.83455878 | 0.007791043 | 0.574148208 | 0.32583607 |
| **High_specific_up** | RAPGEF3 | 0.833106799 | 0.00267572 | 0.524226971 | 0.184687322 |
| **High_specific_up** | SMG7-AS1 | 0.831158229 | 3.77E-05 | 0.43371896 | 0.273705205 |
| **High_specific_up** | BCAR1 | 0.829066144 | 0.004051635 | 0.784876378 | 0.02374798 |
| **High_specific_up** | SP2-AS1 | 0.828494644 | 0.005617416 | 0.489963576 | 0.381018517 |
| **High_specific_up** | GPR153 | 0.826426115 | 8.36E-07 | 0.29176846 | 0.497133125 |
| **High_specific_up** | CDKL1 | 0.826161737 | 0.000570737 | 0.619194651 | 0.048045743 |

| Category | | High_vs_control | | Low_vs_control | |
|---|---|---|---|---|---|
| *Type* | *gene* | *FC(fold change)* | *FDR* | *FC(fold change)* | *FDR* |
| **High_specific_up** | AC006116.10 | 0.819382463 | 3.03E-05 | 0.413316252 | 0.185317066 |
| **High_specific_up** | LHX4 | 0.815447586 | 1.06E-05 | 0.30422647 | 0.359838924 |
| **High_specific_up** | MFSD6 | 0.814113031 | 0.001284415 | 0.584331355 | 0.171698482 |
| **High_specific_up** | CEBPB-AS1 | 0.812269233 | 5.97E-06 | 0.431295699 | 0.154589285 |
| **High_specific_up** | CHRNG | 0.810458022 | 0.001753119 | 0.425152554 | 0.301144699 |
| **High_specific_up** | AC134407.3 | 0.808698128 | 0.003530363 | 0.709360753 | 0.020708403 |
| **High_specific_up** | RNF215 | 0.805297946 | 1.24E-06 | 0.520874671 | 0.014645275 |
| **High_specific_up** | TSSK4 | 0.804791204 | 3.82E-05 | 0.244378706 | 0.613456795 |
| **High_specific_up** | CIRBP-AS1 | 0.804187978 | 8.14E-06 | 0.23393932 | 0.672027899 |
| **High_specific_up** | AC036176.1 | 0.803922934 | 0.000853192 | 0.146270656 | 0.803142464 |
| **High_specific_up** | ADH6 | 0.803351188 | 0.00377995 | -0.118682132 | 0.897338344 |
| **High_specific_up** | ZBTB32 | 0.801487129 | 2.54E-05 | 0.400849797 | 0.123982822 |
| **High_specific_up** | HM13-AS1 | 0.798524946 | 0.006626089 | 0.667663045 | 0.154478105 |
| **High_specific_up** | AC092944.1 | 0.793057257 | 0.001670768 | 0.431170848 | 0.238870516 |
| **High_specific_up** | MASP2 | 0.791994506 | 0.000592654 | 0.425200113 | 0.328653312 |
| **High_specific_up** | C11orf95 | 0.79156507 | 0.001630472 | 0.362358223 | 0.440557743 |
| **High_specific_up** | WIPI1 | 0.785172579 | 0.003338548 | 0.45903079 | 0.235614729 |
| **High_specific_up** | WNT2B | 0.774859199 | 1.38E-05 | 0.4199968 | 0.095081512 |
| **High_specific_up** | ZNF532 | 0.772823046 | 0.00596628 | 0.215213566 | 0.753097443 |
| **High_specific_up** | PBLD | 0.771626224 | 0.005372699 | 0.3482147 | 0.343778554 |
| **High_specific_up** | AC010998.2 | 0.770413021 | 0.002590626 | 0.243295625 | 0.645384628 |
| **High_specific_up** | SLC1A4 | 0.769050332 | 0.00284569 | 0.772133089 | 0.056800259 |
| **High_specific_up** | PALLD | 0.768887034 | 0.002265888 | 0.687979872 | 0.049022844 |
| **High_specific_up** | ZNF587 | 0.768290113 | 0.000397221 | 0.413268953 | 0.253101174 |
| **High_specific_up** | MARCHF1 | 0.747673313 | 0.005610483 | 0.433910829 | 0.140316992 |
| **High_specific_up** | GALNT7 | 0.747089243 | 0.003826216 | 0.42672117 | 0.391807537 |
| **High_specific_up** | MZF1-AS1 | 0.744413166 | 0.000170015 | 0.507479775 | 0.060093245 |
| **High_specific_up** | AC093752.3 | 0.744048617 | 0.002780112 | 0.080373005 | 0.912356483 |
| **High_specific_up** | AC009090.6 | 0.739735467 | 6.79E-05 | 0.350564127 | 0.203447058 |
| **High_specific_up** | ZFPM1 | 0.732128047 | 0.002767914 | 0.31663164 | 0.524283966 |
| **High_specific_up** | CARD14 | 0.728658383 | 0.000319477 | 0.406946401 | 0.206414285 |
| **High_specific_up** | NFATC4 | 0.727203975 | 0.003384612 | 0.219311283 | 0.710476632 |
| **High_specific_up** | TCF7L1 | 0.726328266 | 0.0020176 | 0.342749323 | 0.441176003 |
| **High_specific_up** | PHLDA1 | 0.725788799 | 0.000737397 | 0.547561166 | 0.074048905 |
| **High_specific_up** | SLC27A1 | 0.721538274 | 0.006187574 | 0.302168809 | 0.575155247 |
| **High_specific_up** | TIMP3 | 0.716209897 | 0.001519201 | 0.639354697 | 0.070452626 |
| **High_specific_up** | TFPT | 0.712753108 | 3.11E-05 | 0.460338998 | 0.094470193 |
| **High_specific_up** | ANO8 | 0.71244533 | 0.000408826 | 0.410792216 | 0.217209117 |
| **High_specific_up** | AC084018.2 | 0.711931172 | 0.004133886 | 0.268267839 | 0.518360256 |
| **High_specific_up** | DGAT1 | 0.711637623 | 0.002151271 | 0.221601022 | 0.676028522 |
| **High_specific_up** | DUOX1 | 0.710563824 | 0.001466117 | 0.344503534 | 0.476561596 |
| **High_specific_up** | PRPF40A | 0.705384811 | 0.003166027 | 0.402230423 | 0.32534487 |
| **High_specific_up** | RNF40 | 0.699117369 | 0.005980461 | 0.421923256 | 0.327650362 |
| **High_specific_up** | PBRM1 | 0.698655485 | 0.001581265 | 0.132512737 | 0.831968573 |
| **High_specific_up** | MYH7B | 0.698259017 | 0.000669076 | 0.598129901 | 0.026250906 |
| **High_specific_up** | EMC1 | 0.694013965 | 0.002442442 | 0.376668724 | 0.403796252 |
| **High_specific_up** | C2orf68 | 0.693173268 | 0.002414469 | 0.655731549 | 0.030090128 |
| **High_specific_up** | MED8-AS1 | 0.692910461 | 7.69E-06 | 0.430829666 | 0.056410335 |
| **High_specific_up** | SPON1 | 0.692195614 | 0.000622984 | 0.573492373 | 0.018370304 |
| **High_specific_up** | ABTB2 | 0.691124246 | 0.001870732 | 0.119960434 | 0.859830327 |
| **High_specific_up** | AL162739.1 | 0.680416775 | 0.006845734 | 0.549774882 | 0.017037884 |
| **High_specific_up** | PCOLCE | 0.676201331 | 0.007539232 | 0.558802029 | 0.347264415 |

| Category | | High_vs_control | | Low_vs_control | |
|---|---|---|---|---|---|
| *Type* | *gene* | *FC(fold change)* | *FDR* | *FC(fold change)* | *FDR* |
| **High_specific_up** | AL392023.2 | 0.675866362 | 0.007317447 | 0.385736324 | 0.322104633 |
| **High_specific_up** | TEX2 | 0.674964321 | 0.000342116 | 0.267457251 | 0.250685991 |
| **High_specific_up** | PDE6B | 0.674502129 | 0.003035627 | 0.258127894 | 0.468967312 |
| **High_specific_up** | HYI | 0.667712122 | 2.05E-05 | 0.246088615 | 0.515837358 |
| **High_specific_up** | AL391839.2 | 0.664136609 | 0.002211288 | 0.660795569 | 0.023033697 |
| **High_specific_up** | AC138409.1 | 0.660952924 | 4.07E-05 | 0.740865634 | 0.06652698 |
| **High_specific_up** | DDX19B | 0.660498853 | 0.009515648 | 0.500839824 | 0.170676745 |
| **High_specific_up** | ASCC1 | 0.648363199 | 0.000201765 | 0.449115274 | 0.051574658 |
| **High_specific_up** | TSPAN4 | 0.647041805 | 3.50E-05 | 0.286385795 | 0.33505683 |
| **High_specific_up** | FHOD1 | 0.64548765 | 0.006400382 | 0.329544121 | 0.476349713 |
| **High_specific_up** | BTBD2 | 0.643782511 | 0.001840633 | 0.331367457 | 0.314078752 |
| **High_specific_up** | AC009065.3 | 0.643558206 | 0.004801363 | 0.639982978 | 0.017110576 |
| **High_specific_up** | AC138028.2 | 0.638441572 | 1.44E-06 | 0.475733123 | 0.011832985 |
| **High_specific_up** | SLC20A2 | 0.635098107 | 0.003937505 | 0.57302188 | 0.017765627 |
| **High_specific_up** | AC060780.1 | 0.634561125 | 0.000738442 | 0.380256594 | 0.259466484 |
| **High_specific_up** | TRIM41 | 0.633289888 | 0.000913326 | 0.369504538 | 0.263095917 |
| **High_specific_up** | AL121787.1 | 0.626381891 | 0.009623366 | 0.436545996 | 0.239950169 |
| **High_specific_up** | AKT1S1 | 0.626003331 | 0.009319128 | 0.14196738 | 0.820215265 |
| **High_specific_up** | MAMDC4 | 0.624611987 | 0.001652905 | 0.134121607 | 0.790896271 |
| **High_specific_up** | TRAF3IP3 | 0.623340564 | 0.008249842 | 0.249796961 | 0.642995748 |
| **High_specific_up** | AC015849.3 | 0.611313228 | 0.001584436 | 0.371055823 | 0.355044218 |
| **High_specific_up** | TYMS | 0.607104279 | 0.000477751 | 0.344744939 | 0.343369086 |
| **High_specific_up** | PTOV1 | 0.603236146 | 0.000407569 | 0.37016009 | 0.20727758 |
| **High_specific_up** | AC008537.4 | 0.591456566 | 3.60E-05 | 0.532790327 | 0.031888529 |
| **High_specific_up** | CYP1B1-AS1 | 0.590407898 | 0.002179537 | 0.511508087 | 0.046530771 |
| **High_specific_up** | SAE1 | 0.582878617 | 0.00064403 | 0.425055291 | 0.164304928 |
| **High_specific_up** | NAPSB | 0.576865411 | 0.002584692 | 0.275882802 | 0.486803362 |
| **High_specific_up** | LMNTD2 | 0.573249689 | 0.003409947 | 0.263334129 | 0.54441565 |
| **High_specific_up** | ZNF827 | 0.571184572 | 0.000648875 | 0.715024886 | 0.068261179 |
| **High_specific_up** | WAC | 0.571136175 | 0.005439367 | 0.5398726 | 0.065610099 |
| **High_specific_up** | CDR1 | 0.569988414 | 4.43E-05 | 0.503327636 | 0.037719474 |
| **High_specific_up** | DM1-AS | 0.566074916 | 5.92E-05 | 0.354589843 | 0.178069609 |
| **High_specific_up** | AC002563.1 | 0.562457142 | 0.004617048 | 0.4457817 | 0.092151609 |
| **High_specific_up** | NEIL1 | 0.561890897 | 2.57E-05 | 0.416562213 | 0.026538738 |
| **High_specific_up** | SDR39U1 | 0.556877255 | 0.000200227 | 0.415045848 | 0.030476258 |
| **High_specific_up** | AC138028.4 | 0.552860635 | 0.000108364 | 0.436508494 | 0.021341091 |
| **High_specific_up** | GMPR2 | 0.547059707 | 0.005579649 | 0.361435167 | 0.263735097 |
| **High_specific_up** | NEPRO | 0.537703064 | 8.14E-05 | 0.198659775 | 0.470174311 |
| **High_specific_up** | AC008269.1 | 0.532574372 | 0.004376304 | 0.573320637 | 0.026571328 |
| **High_specific_up** | AC073130.3 | 0.52507678 | 0.001267063 | 0.271631652 | 0.325274518 |
| **High_specific_up** | LRP1-AS | 0.518377408 | 0.004054061 | 0.530199137 | 0.02985885 |
| **High_specific_up** | THUMPD3 | 0.517505334 | 0.009016562 | 0.359302442 | 0.240908539 |
| **High_specific_up** | CDK4 | 0.515308754 | 0.007264997 | 0.27720305 | 0.421723889 |
| **High_specific_up** | LAMA5-AS1 | 0.510363774 | 0.001029748 | 0.270357607 | 0.291562602 |
| **High_specific_up** | ARMC5 | 0.508360806 | 0.008599401 | 0.200279723 | 0.612094257 |
| **High_specific_up** | CIZ1 | 0.507218432 | 0.000599299 | 0.12816866 | 0.693955349 |
| **High_specific_up** | WDR73 | 0.507175619 | 0.002670491 | 0.218741732 | 0.468967312 |
| **High_specific_up** | DPY19L2P3 | 0.504566262 | 0.001488046 | 0.274449942 | 0.275926885 |
| **High_specific_up** | AC011611.3 | 0.501817208 | 9.20E-05 | 0.337614757 | 0.072281344 |
| **High_specific_up** | NRIP2 | 0.501801684 | 0.005417894 | 0.411256015 | 0.103933551 |
| **High_specific_up** | GARS1-DT | 0.497791815 | 0.006769277 | 0.357830091 | 0.088657608 |
| **High_specific_up** | LINC00847 | 0.495278498 | 0.002536446 | 0.229128212 | 0.410160427 |

| Category | | High_vs_control | | Low_vs_control | |
|---|---|---|---|---|---|
| *Type* | *gene* | *FC(fold change)* | *FDR* | *FC(fold change)* | *FDR* |
| **High_specific_up** | AL353593.1 | 0.492146763 | 0.00131829 | 0.592479392 | 0.011699122 |
| **High_specific_up** | AC087521.4 | 0.480781556 | 0.001278188 | 0.530568848 | 0.011560086 |
| **High_specific_up** | AC131571.1 | 0.47297669 | 0.007244236 | 0.396445779 | 0.108313017 |
| **High_specific_up** | ATXN2 | 0.459848183 | 0.002211288 | 0.606976001 | 0.010455425 |
| **High_specific_up** | AC234782.4 | 0.453841195 | 0.0004442 | 0.359643789 | 0.037935248 |
| **High_specific_up** | AC126696.2 | 0.45194211 | 0.000420713 | 0.315709397 | 0.113776064 |
| **High_specific_up** | MPI | 0.443350393 | 0.006126015 | 0.291618549 | 0.186746205 |
| **High_specific_up** | CARS1-AS1 | 0.438606848 | 0.0002035 | 0.339244687 | 0.0413443 |
| **High_specific_up** | RGL4 | 0.435832128 | 0.009866952 | 0.289999372 | 0.268033673 |
| **High_specific_up** | FBXO25 | 0.429758277 | 0.003114412 | 0.263853202 | 0.327042976 |
| **High_specific_up** | AL135999.1 | 0.428617016 | 0.00507509 | 0.313697575 | 0.120724207 |
| **High_specific_up** | AC008403.2 | 0.427448778 | 0.008489784 | 0.188035508 | 0.596403286 |
| **High_specific_up** | TCP11L1 | 0.426682278 | 0.001193512 | 0.299540011 | 0.183006963 |
| **High_specific_up** | PPM1F-AS1 | 0.417042301 | 0.001620174 | 0.252415113 | 0.213527715 |
| **High_specific_up** | ARPC4 | 0.414379377 | 0.006652234 | 0.383728676 | 0.242899454 |
| **High_specific_up** | FAM220CP | 0.411371808 | 0.005405914 | 0.526871145 | 0.142355761 |
| **High_specific_up** | KDM4A-AS1 | 0.346609222 | 0.004859071 | 0.221265401 | 0.36490522 |
| **common_up** | LINC02252 | 7.464521684 | 2.63E-09 | 5.567615904 | 0.008637358 |
| **common_up** | CBX4 | 5.753005407 | 0.001309858 | 5.539293278 | 0.009421454 |
| **common_up** | AC061975.4 | 5.187202699 | 2.57E-07 | 4.324269418 | 0.001288355 |
| **common_up** | AC105105.1 | 5.08978794 | 3.20E-31 | 3.738926761 | 7.10E-09 |
| **common_up** | AC027243.1 | 4.81658374 | 3.42E-38 | 3.92611551 | 2.58E-12 |
| **common_up** | HTR5A-AS1 | 4.762760186 | 0.000262568 | 4.428278161 | 0.008300301 |
| **common_up** | PYY | 4.617834496 | 0.000110047 | 4.270947079 | 0.002182594 |
| **common_up** | TMC2 | 4.511905088 | 1.17E-07 | 4.457279563 | 0.000580198 |
| **common_up** | IKZF5 | 4.365503224 | 0.000582162 | 4.590390359 | 0.000194343 |
| **common_up** | CROCC2 | 4.342266215 | 6.80E-05 | 3.937741778 | 0.000485733 |
| **common_up** | AC007326.2 | 4.22405831 | 1.08E-10 | 3.961991386 | 2.36E-05 |
| **common_up** | LRIT2 | 4.030729296 | 4.28E-06 | 3.77241571 | 0.002231433 |
| **common_up** | AC092894.1 | 3.984299914 | 0.003705639 | 4.14826884 | 0.006862968 |
| **common_up** | ANKRD34A | 3.931752029 | 0.000496493 | 4.434566747 | 4.74E-06 |
| **common_up** | AL355388.1 | 3.794895251 | 6.25E-28 | 2.352609975 | 5.50E-05 |
| **common_up** | TMPRSS13 | 3.756898482 | 2.41E-11 | 3.52132049 | 1.27E-06 |
| **common_up** | IL21R-AS1 | 3.719466427 | 3.24E-67 | 3.48234805 | 3.03E-48 |
| **common_up** | DDIT4-AS1 | 3.518292362 | 1.15E-80 | 2.839974373 | 3.03E-48 |
| **common_up** | AC012313.1 | 3.339218832 | 3.43E-65 | 3.162480299 | 1.11E-26 |
| **common_up** | LINC00963 | 2.969841842 | 3.84E-07 | 2.533229641 | 0.002372469 |
| **common_up** | AC005326.1 | 2.855956086 | 1.56E-17 | 2.219915391 | 2.72E-06 |
| **common_up** | AC016769.4 | 2.773297297 | 0.000753017 | 2.893668568 | 0.007817552 |
| **common_up** | AL157414.1 | 2.695975443 | 7.97E-10 | 2.793853349 | 5.34E-11 |
| **common_up** | ARSL | 2.670721154 | 1.32E-06 | 2.479678361 | 1.77E-05 |
| **common_up** | TBC1D26 | 2.661661075 | 7.70E-08 | 3.041386769 | 6.78E-10 |
| **common_up** | GSS | 2.636114076 | 4.19E-13 | 2.835149529 | 1.35E-13 |
| **common_up** | MYO15A | 2.629154781 | 8.14E-29 | 2.246248334 | 1.13E-20 |
| **common_up** | MVD | 2.628135932 | 5.30E-11 | 2.268500353 | 1.04E-05 |
| **common_up** | IQCE | 2.591132936 | 0.002502785 | 2.683108076 | 0.0040463 |
| **common_up** | SNAI3 | 2.519908613 | 1.78E-52 | 2.281054969 | 2.50E-35 |
| **common_up** | SIVA1 | 2.503789408 | 1.99E-08 | 2.01384247 | 0.009969581 |
| **common_up** | INHBC | 2.492741815 | 5.02E-25 | 1.939040242 | 1.73E-08 |
| **common_up** | AL109615.3 | 2.446752104 | 2.73E-59 | 2.237023846 | 9.52E-38 |
| **common_up** | SPIRE1 | 2.443861268 | 0.000344095 | 2.615198342 | 0.00045029 |
| **common_up** | LINC02042 | 2.355190761 | 0.009016562 | 2.910260579 | 0.001556765 |

| Category | | High_vs_control | | Low_vs_control | |
|---|---|---|---|---|---|
| *Type* | *gene* | *FC(fold change)* | *FDR* | *FC(fold change)* | *FDR* |
| **common_up** | NLRP7 | 2.337318882 | 0.000140154 | 2.043401357 | 0.009163769 |
| **common_up** | AC090616.1 | 2.291520026 | 9.27E-05 | 2.132856846 | 2.52E-06 |
| **common_up** | PLPPR2 | 2.28632853 | 1.45E-14 | 1.670587881 | 4.87E-07 |
| **common_up** | AC022506.2 | 2.27849262 | 1.04E-38 | 1.832749647 | 3.17E-18 |
| **common_up** | DIXDC1 | 2.246993882 | 3.10E-07 | 2.419101435 | 4.46E-07 |
| **common_up** | NDUFA4L2 | 2.218114453 | 1.71E-20 | 1.333094191 | 0.002817098 |
| **common_up** | BEND6 | 2.186305457 | 0.000659911 | 2.420266335 | 0.000649572 |
| **common_up** | USP24 | 2.14661702 | 1.37E-31 | 1.975404493 | 1.03E-22 |
| **common_up** | OR10AD1 | 2.135933157 | 4.27E-06 | 1.730704515 | 0.000538378 |
| **common_up** | INSIG1 | 2.127786494 | 4.91E-09 | 1.664767293 | 0.000213701 |
| **common_up** | LINC01981 | 2.123383504 | 0.00026637 | 2.13470387 | 0.001363956 |
| **common_up** | CAMK4 | 2.120248646 | 4.40E-16 | 2.009058722 | 2.00E-24 |
| **common_up** | HIBCH | 2.099620908 | 5.78E-05 | 2.227284631 | 9.13E-05 |
| **common_up** | AC005062.1 | 2.080192222 | 6.10E-05 | 1.761600078 | 6.04E-08 |
| **common_up** | PEX1 | 2.030964512 | 0.000408826 | 2.120212746 | 0.001213747 |
| **common_up** | SPAG5 | 2.02005345 | 0.000369083 | 2.111923278 | 0.000580198 |
| **common_up** | AC113133.1 | 2.015332378 | 0.004653427 | 2.421342431 | 2.56E-05 |
| **common_up** | LIMK2 | 2.012546664 | 0.002579973 | 2.900427074 | 2.11E-07 |
| **common_up** | AMN1 | 2.006146152 | 9.28E-06 | 2.32555242 | 1.04E-05 |
| **common_up** | NPPA | 2.002335346 | 1.23E-07 | 1.745977461 | 1.15E-07 |
| **common_up** | STARD13-AS | 2.000473111 | 8.28E-12 | 1.462027661 | 5.13E-05 |
| **common_up** | AC010307.3 | 1.988625876 | 0.004281563 | 2.308254995 | 0.000880258 |
| **common_up** | SLC7A11-AS1 | 1.971629495 | 1.04E-38 | 1.511135784 | 7.82E-19 |
| **common_up** | SLITRK6 | 1.961433474 | 1.96E-09 | 2.194481779 | 4.34E-06 |
| **common_up** | SPIB | 1.951641617 | 3.45E-05 | 1.722423148 | 0.003651502 |
| **common_up** | ZNF132 | 1.926026837 | 1.96E-17 | 1.914961713 | 2.45E-13 |
| **common_up** | POU6F2 | 1.925479638 | 0.003663837 | 2.444135071 | 0.000216735 |
| **common_up** | ALPG | 1.903076639 | 2.22E-06 | 1.606087579 | 0.00646924 |
| **common_up** | AC022087.1 | 1.894566787 | 3.65E-09 | 1.317637489 | 9.93E-08 |
| **common_up** | MACC1 | 1.889193439 | 2.05E-06 | 1.325331904 | 3.92E-05 |
| **common_up** | PCSK9 | 1.881526076 | 1.38E-08 | 2.093266045 | 2.34E-10 |
| **common_up** | AC012313.5 | 1.85695656 | 8.03E-13 | 2.009299356 | 5.34E-14 |
| **common_up** | NPB | 1.834053542 | 3.06E-14 | 1.796008854 | 4.85E-12 |
| **common_up** | HCN3 | 1.816161074 | 2.34E-16 | 1.676033715 | 8.11E-13 |
| **common_up** | MAN2B2 | 1.80137233 | 1.22E-06 | 1.526949967 | 0.001443374 |
| **common_up** | HLA-J | 1.789486349 | 2.59E-09 | 1.244883925 | 5.80E-05 |
| **common_up** | INPP5J | 1.777667094 | 7.60E-19 | 1.638023427 | 3.51E-18 |
| **common_up** | AL445288.1 | 1.777243517 | 0.00019671 | 1.963419863 | 0.000229376 |
| **common_up** | GPRC5C | 1.769875599 | 0.000281715 | 1.714188818 | 0.003849393 |
| **common_up** | OLMALINC | 1.734810706 | 0.000592947 | 1.884442733 | 0.001401053 |
| **common_up** | AC025857.2 | 1.731197728 | 1.99E-07 | 1.499069943 | 0.001307736 |
| **common_up** | TAC4 | 1.717607339 | 7.52E-10 | 1.356112643 | 0.002014975 |
| **common_up** | AC124944.1 | 1.706644347 | 0.000164244 | 1.618876596 | 0.000624339 |
| **common_up** | AC068134.2 | 1.706201733 | 0.00167075 | 1.948634403 | 0.000310967 |
| **common_up** | ENG | 1.697348556 | 1.47E-12 | 1.058302157 | 0.001199705 |
| **common_up** | TYK2 | 1.696884254 | 3.27E-07 | 1.289640189 | 0.004346867 |
| **common_up** | MFSD4A | 1.685700469 | 1.19E-05 | 1.41883361 | 4.89E-06 |
| **common_up** | TMED4 | 1.672741781 | 6.20E-11 | 1.131152952 | 0.000224367 |
| **common_up** | AL136131.3 | 1.6659259 | 4.27E-25 | 1.062338391 | 3.07E-05 |
| **common_up** | RGMB-AS1 | 1.657407948 | 2.19E-27 | 1.040182013 | 1.72E-07 |
| **common_up** | AC017028.2 | 1.650327819 | 0.000709094 | 1.575127166 | 0.003880431 |
| **common_up** | ACSS2 | 1.644747114 | 4.12E-06 | 1.302513854 | 0.00137726 |

| Category | | High_vs_control | | Low_vs_control | |
|---|---|---|---|---|---|
| *Type* | *gene* | *FC(fold change)* | *FDR* | *FC(fold change)* | *FDR* |
| **common_up** | C12orf45 | 1.617534571 | 9.47E-12 | 1.189018863 | 0.000557028 |
| **common_up** | FLVCR2 | 1.606195504 | 3.33E-07 | 1.729461609 | 8.06E-08 |
| **common_up** | MYO15B | 1.602969022 | 1.18E-13 | 1.160699869 | 0.000124758 |
| **common_up** | AC084337.1 | 1.596446425 | 1.25E-06 | 1.241319002 | 0.001581704 |
| **common_up** | BNIP3L | 1.595322482 | 6.64E-11 | 1.152942416 | 0.000421997 |
| **common_up** | SLC16A11 | 1.583028689 | 6.20E-09 | 1.515005241 | 9.53E-06 |
| **common_up** | ENTPD1-AS1 | 1.577173384 | 6.20E-09 | 1.361507635 | 2.25E-11 |
| **common_up** | ZNRF1 | 1.573047392 | 5.38E-15 | 1.233192727 | 0.000216718 |
| **common_up** | AP001468.1 | 1.5720255 | 2.14E-08 | 1.513707389 | 4.53E-10 |
| **common_up** | PCA3 | 1.566042069 | 5.35E-34 | 1.784499994 | 1.44E-31 |
| **common_up** | DDX56 | 1.564882758 | 7.42E-07 | 1.613066962 | 1.28E-05 |
| **common_up** | AL034548.2 | 1.564659053 | 9.17E-13 | 1.25473217 | 5.75E-05 |
| **common_up** | ZNHIT2 | 1.556491735 | 7.83E-11 | 1.551744559 | 4.69E-10 |
| **common_up** | GCC2 | 1.549896649 | 0.000304819 | 1.500450215 | 3.60E-06 |
| **common_up** | TDRKH | 1.502266621 | 0.000121244 | 1.480052922 | 0.001636085 |
| **common_up** | KLF11 | 1.479147955 | 0.001840633 | 1.545743454 | 0.000957351 |
| **common_up** | AC099489.3 | 1.468508782 | 0.001082339 | 1.529629062 | 0.007594578 |
| **common_up** | CSKMT | 1.451706555 | 2.81E-11 | 2.124943484 | 1.93E-26 |
| **common_up** | RANGRF | 1.434764831 | 2.55E-08 | 1.24431416 | 2.92E-05 |
| **common_up** | AC010834.3 | 1.432113773 | 7.82E-09 | 1.490697002 | 8.07E-07 |
| **common_up** | MTND5P11 | 1.431003855 | 0.00204011 | 1.733648569 | 0.002232859 |
| **common_up** | LIN7B | 1.41707507 | 0.000430229 | 1.420752104 | 0.001473747 |
| **common_up** | KHK | 1.409318319 | 4.72E-09 | 1.169485247 | 1.04E-05 |
| **common_up** | BHLHE40-AS1 | 1.394794892 | 1.93E-08 | 1.32604051 | 1.13E-06 |
| **common_up** | AC011370.1 | 1.394170357 | 0.000928386 | 1.722820636 | 0.003963742 |
| **common_up** | BMP8A | 1.390839771 | 8.72E-13 | 0.757459316 | 0.009831638 |
| **common_up** | CHRM3-AS2 | 1.389554712 | 0.001098398 | 1.846369128 | 0.002484495 |
| **common_up** | SLC16A8 | 1.389311766 | 3.79E-06 | 1.151995081 | 0.003880431 |
| **common_up** | ACSM6 | 1.388821671 | 5.35E-07 | 0.941909623 | 0.000221796 |
| **common_up** | MUC16 | 1.386919181 | 0.000315887 | 1.956041896 | 0.00632684 |
| **common_up** | AL512353.1 | 1.38424128 | 4.05E-10 | 1.149214023 | 2.37E-06 |
| **common_up** | RASIP1 | 1.381296114 | 0.005184049 | 1.601587345 | 0.003665539 |
| **common_up** | MYOF | 1.378541334 | 4.29E-09 | 0.808534496 | 0.006969988 |
| **common_up** | AC114947.2 | 1.370119983 | 1.08E-32 | 1.444475547 | 1.93E-26 |
| **common_up** | TBX10 | 1.356753009 | 5.19E-12 | 0.910061084 | 0.000915253 |
| **common_up** | HEXIM1 | 1.343722458 | 8.37E-06 | 1.095085839 | 0.000379351 |
| **common_up** | MYO5B | 1.341502615 | 3.29E-06 | 1.185404774 | 0.002279312 |
| **common_up** | SRRM4 | 1.336493654 | 1.10E-08 | 1.221433321 | 1.23E-06 |
| **common_up** | HSPB7 | 1.320705056 | 1.35E-07 | 0.866310852 | 0.004881281 |
| **common_up** | C6orf223 | 1.298148216 | 1.24E-05 | 1.0931718 | 0.008252449 |
| **common_up** | TSPAN11 | 1.292578227 | 0.000132593 | 1.257840785 | 2.13E-05 |
| **common_up** | AC068831.6 | 1.289244231 | 0.002161237 | 1.268624789 | 0.003967648 |
| **common_up** | UFD1 | 1.285244032 | 4.36E-10 | 0.87635655 | 3.91E-05 |
| **common_up** | LINC01465 | 1.277032158 | 4.48E-14 | 0.760646509 | 0.001242103 |
| **common_up** | CCDC57 | 1.251772815 | 7.88E-09 | 1.044164935 | 3.16E-05 |
| **common_up** | SLC13A4 | 1.251279494 | 6.53E-07 | 0.936320919 | 0.000379351 |
| **common_up** | INHBE | 1.240977563 | 8.23E-10 | 1.289609506 | 9.38E-09 |
| **common_up** | NFE2L3 | 1.240924418 | 1.32E-10 | 0.820024948 | 1.32E-05 |
| **common_up** | CACFD1 | 1.238754012 | 3.77E-06 | 1.353932626 | 4.74E-06 |
| **common_up** | TNS2 | 1.233590525 | 3.04E-12 | 1.046578939 | 2.29E-08 |
| **common_up** | AL022322.2 | 1.233036055 | 1.02E-05 | 0.8412453 | 0.000807244 |
| **common_up** | AC022532.1 | 1.223286078 | 4.48E-14 | 1.115345213 | 3.95E-13 |

| Category | | High_vs_control | | Low_vs_control | |
|---|---|---|---|---|---|
| *Type* | *gene* | *FC(fold change)* | *FDR* | *FC(fold change)* | *FDR* |
| **common_up** | IGLON5 | 1.221123885 | 5.56E-05 | 1.060127692 | 0.002414199 |
| **common_up** | GP6 | 1.20931052 | 6.04E-06 | 1.159928387 | 9.92E-05 |
| **common_up** | CYP2D6 | 1.203368959 | 7.27E-08 | 0.822571795 | 0.002402344 |
| **common_up** | TSHZ2 | 1.196738543 | 1.14E-07 | 0.989737616 | 0.006862968 |
| **common_up** | IDI2-AS1 | 1.193382709 | 6.24E-28 | 1.184667811 | 1.31E-12 |
| **common_up** | AC092881.2 | 1.189395872 | 3.83E-08 | 0.975396381 | 9.67E-05 |
| **common_up** | AL445309.1 | 1.188409998 | 3.90E-06 | 1.028011017 | 0.002120286 |
| **common_up** | NUDCD2 | 1.188295059 | 4.57E-13 | 0.739300806 | 0.00025639 |
| **common_up** | FASN | 1.184563985 | 4.10E-12 | 1.269555531 | 1.86E-11 |
| **common_up** | FAM20A | 1.182526135 | 6.64E-11 | 0.906559748 | 0.001091382 |
| **common_up** | TMEM231 | 1.178301272 | 0.001052083 | 1.145038305 | 0.003285968 |
| **common_up** | CTSB | 1.168584775 | 1.03E-10 | 0.98073789 | 1.29E-10 |
| **common_up** | UQCC3 | 1.160189194 | 6.18E-07 | 1.869653119 | 3.93E-29 |
| **common_up** | C16orf54 | 1.149579281 | 6.53E-07 | 1.096777443 | 0.000950587 |
| **common_up** | GCNT1 | 1.147246449 | 1.04E-08 | 1.245728843 | 1.80E-08 |
| **common_up** | TCP1 | 1.14723544 | 3.82E-20 | 1.175813546 | 5.72E-17 |
| **common_up** | GPR39 | 1.129467807 | 1.91E-10 | 1.129175981 | 4.67E-07 |
| **common_up** | ARSG | 1.124847032 | 2.03E-08 | 1.050678169 | 3.12E-05 |
| **common_up** | DGCR6 | 1.118979745 | 1.07E-21 | 1.107177617 | 9.62E-14 |
| **common_up** | PSMA3 | 1.116046055 | 0.000137711 | 1.040677008 | 2.21E-05 |
| **common_up** | AL133346.1 | 1.115387643 | 7.14E-13 | 0.712169671 | 0.000885906 |
| **common_up** | MLST8 | 1.112892996 | 6.15E-09 | 0.893548286 | 0.000193335 |
| **common_up** | DHCR7 | 1.098174433 | 0.00255874 | 1.233910582 | 0.006165724 |
| **common_up** | LIPG | 1.095428294 | 6.34E-05 | 1.110858113 | 0.000368282 |
| **common_up** | YPEL4 | 1.088402911 | 7.01E-09 | 0.732930625 | 0.004790115 |
| **common_up** | AP001469.1 | 1.084983841 | 1.34E-21 | 1.11613227 | 1.78E-15 |
| **common_up** | MAP1B | 1.080417526 | 0.002127432 | 1.142499511 | 0.003841873 |
| **common_up** | CCNYL1 | 1.077028008 | 7.05E-07 | 0.874449202 | 8.78E-05 |
| **common_up** | USP45 | 1.076751445 | 0.00160402 | 0.813375307 | 0.009321276 |
| **common_up** | HEBP2 | 1.069981676 | 4.79E-05 | 0.99020855 | 0.002275262 |
| **common_up** | SAPCD1-AS1 | 1.069655669 | 1.16E-05 | 0.799926021 | 0.003527703 |
| **common_up** | TMEM30A-DT | 1.0578607 | 2.39E-18 | 0.629515506 | 1.29E-05 |
| **common_up** | VLDLR-AS1 | 1.05634658 | 4.16E-18 | 0.8090674 | 2.51E-08 |
| **common_up** | CERT1 | 1.033006397 | 1.06E-24 | 1.231312773 | 2.60E-24 |
| **common_up** | AC004477.1 | 1.029738802 | 4.08E-27 | 0.944749869 | 7.59E-11 |
| **common_up** | AC002351.1 | 1.021761287 | 2.51E-08 | 0.756327808 | 0.000901209 |
| **common_up** | AP001107.9 | 1.015536338 | 9.87E-11 | 1.083317826 | 5.22E-12 |
| **common_up** | LRFN4 | 1.014676697 | 0.00018059 | 0.918800716 | 0.002394735 |
| **common_up** | JAKMIP2-AS1 | 1.009448091 | 6.23E-09 | 1.192145993 | 2.48E-10 |
| **common_up** | SMARCB1 | 1.003937278 | 6.11E-16 | 1.212403578 | 4.39E-12 |
| **common_up** | AGBL3 | 0.997549677 | 0.00364007 | 1.258413779 | 0.000929781 |
| **common_up** | AL670729.3 | 0.989569934 | 8.21E-12 | 0.761972158 | 0.001255775 |
| **common_up** | AC104794.5 | 0.986970796 | 7.78E-06 | 0.831856617 | 0.009228962 |
| **common_up** | DBNL | 0.984565588 | 1.54E-05 | 0.796634118 | 0.00145525 |
| **common_up** | DHRS1 | 0.977966212 | 1.80E-08 | 0.759726022 | 0.001766931 |
| **common_up** | STRA6 | 0.974374038 | 3.23E-06 | 0.775486605 | 0.001371021 |
| **common_up** | AP001372.2 | 0.958647431 | 0.000338483 | 1.120017701 | 5.91E-05 |
| **common_up** | TSEN54 | 0.945475575 | 1.06E-09 | 0.853284294 | 9.93E-08 |
| **common_up** | DCPS | 0.937965949 | 0.003190931 | 0.93973008 | 0.000716061 |
| **common_up** | IFT20 | 0.931704892 | 9.42E-12 | 1.124638393 | 1.39E-14 |
| **common_up** | AC007743.1 | 0.928387525 | 0.00118559 | 1.069005287 | 6.19E-05 |
| **common_up** | ZSCAN30 | 0.923444181 | 0.007539232 | 0.807459092 | 0.008300301 |

| Category | | High_vs_control | | Low_vs_control | |
|---|---|---|---|---|---|
| *Type* | *gene* | *FC(fold change)* | *FDR* | *FC(fold change)* | *FDR* |
| **common_up** | AMZ1 | 0.920522715 | 0.002954848 | 1.231828609 | 0.000296409 |
| **common_up** | HEXIM2 | 0.917594784 | 2.05E-05 | 0.850789308 | 0.003860913 |
| **common_up** | BPTF | 0.906020211 | 3.16E-05 | 0.765355411 | 0.000581202 |
| **common_up** | AL450467.1 | 0.898126251 | 4.16E-12 | 0.735059238 | 4.95E-07 |
| **common_up** | AC108134.1 | 0.89736493 | 3.46E-11 | 0.485636692 | 0.006624115 |
| **common_up** | MVK | 0.881917673 | 3.53E-07 | 0.829425733 | 6.58E-05 |
| **common_up** | MUC3A | 0.881080947 | 5.42E-06 | 1.153094884 | 8.91E-05 |
| **common_up** | AP001469.3 | 0.877374945 | 2.94E-07 | 0.84243867 | 2.63E-05 |
| **common_up** | TMEM80 | 0.871481732 | 0.006226059 | 0.886787568 | 0.004929357 |
| **common_up** | AC006030.1 | 0.865434254 | 1.72E-12 | 0.673213429 | 1.25E-05 |
| **common_up** | TNFRSF25 | 0.853911823 | 6.31E-10 | 0.793661756 | 0.00013239 |
| **common_up** | C1orf167 | 0.853422906 | 1.04E-10 | 0.844938095 | 1.90E-07 |
| **common_up** | SMPD5 | 0.850673269 | 2.24E-05 | 0.76443037 | 0.001126705 |
| **common_up** | XACT | 0.848996503 | 6.60E-06 | 0.663728086 | 0.002289161 |
| **common_up** | PDE2A | 0.841409071 | 0.000177736 | 1.074755568 | 2.47E-06 |
| **common_up** | DDX6 | 0.838391101 | 7.86E-06 | 0.765659869 | 0.006507144 |
| **common_up** | PDE2A-AS2 | 0.831666936 | 0.008708048 | 1.266532537 | 3.68E-05 |
| **common_up** | CBY1 | 0.81354138 | 0.001043995 | 0.886096736 | 0.001268705 |
| **common_up** | ARHGAP33 | 0.812890921 | 0.005119097 | 0.820885573 | 0.007109278 |
| **common_up** | DYNC1LI1 | 0.805261661 | 0.003942829 | 0.790722677 | 0.006925702 |
| **common_up** | AC138028.5 | 0.804188928 | 1.48E-07 | 0.569624055 | 0.004929357 |
| **common_up** | ACBD6 | 0.80044295 | 0.002494527 | 1.165503086 | 9.92E-08 |
| **common_up** | LINC02603 | 0.794652859 | 0.006614124 | 0.548175448 | 0.005733516 |
| **common_up** | TLCD1 | 0.793504907 | 2.46E-05 | 0.620270938 | 0.009377214 |
| **common_up** | AC093462.1 | 0.784263466 | 0.000295657 | 0.785981965 | 0.004531444 |
| **common_up** | TMEM8B | 0.769570997 | 4.41E-05 | 0.681856097 | 0.003093896 |
| **common_up** | AC005632.6 | 0.765437161 | 1.42E-05 | 0.74643488 | 0.002001326 |
| **common_up** | CYP51A1-AS1 | 0.758733998 | 0.000277024 | 0.683722976 | 0.007888049 |
| **common_up** | AC079601.1 | 0.755067108 | 3.73E-09 | 0.493389751 | 0.003004893 |
| **common_up** | AC067969.2 | 0.750542412 | 0.000440488 | 0.733635231 | 0.004154418 |
| **common_up** | RNF213-AS1 | 0.749695629 | 6.77E-08 | 0.72719377 | 0.00470055 |
| **common_up** | MCM3AP | 0.748924706 | 2.13E-05 | 0.791519832 | 0.000102013 |
| **common_up** | AL049840.2 | 0.744758736 | 0.000514861 | 0.766153739 | 0.008410355 |
| **common_up** | CXCR6 | 0.7388634 | 0.001430762 | 0.882708866 | 0.004227943 |
| **common_up** | RUSC1-AS1 | 0.732396717 | 3.32E-10 | 0.735785924 | 9.06E-08 |
| **common_up** | SLC4A5 | 0.730930922 | 7.06E-07 | 0.63525695 | 0.000368282 |
| **common_up** | FDFT1 | 0.730722003 | 0.006975113 | 0.900612554 | 0.003905727 |
| **common_up** | OGFOD3 | 0.71175382 | 0.000905386 | 0.77563156 | 0.001917292 |
| **common_up** | CASC11 | 0.710104509 | 1.01E-11 | 0.62661592 | 1.04E-05 |
| **common_up** | LDLRAD2 | 0.703497897 | 2.93E-07 | 0.657816899 | 7.69E-06 |
| **common_up** | TBL3 | 0.701588406 | 0.007540935 | 1.041392708 | 0.000223235 |
| **common_up** | BACE1-AS | 0.697135153 | 1.38E-05 | 0.544126036 | 0.006138791 |
| **common_up** | AC125257.2 | 0.64465064 | 1.59E-10 | 0.798706094 | 5.09E-10 |
| **common_up** | RMC1 | 0.64163686 | 4.26E-05 | 0.652117595 | 0.000713997 |
| **common_up** | AC087521.2 | 0.636993067 | 3.27E-07 | 0.634819111 | 1.00E-05 |
| **common_up** | SPC24 | 0.625941404 | 3.89E-07 | 0.957333769 | 2.52E-12 |
| **common_up** | LINC01090 | 0.614497084 | 0.000451301 | 0.541599372 | 0.004760893 |
| **common_up** | SIGLEC10 | 0.609592906 | 0.000686885 | 0.618620999 | 5.90E-05 |
| **common_up** | AL121753.2 | 0.562114642 | 0.000738442 | 0.719614705 | 0.000580198 |
| **common_up** | AC011473.2 | 0.558293344 | 0.008670173 | 0.661677368 | 0.008637358 |
| **common_up** | PCAT1 | 0.546259105 | 7.49E-05 | 0.505244035 | 0.000731893 |
| **common_up** | MMP24OS | 0.542572811 | 0.001044675 | 0.702371648 | 5.13E-05 |

| Category | | High_vs_control | | Low_vs_control | |
|---|---|---|---|---|---|
| *Type* | *gene* | *FC(fold change)* | *FDR* | *FC(fold change)* | *FDR* |
| **common_up** | LTBR | 0.4642942 | 0.009138769 | 0.617316085 | 0.0040463 |
| **common_up** | SIK2 | 0.436205116 | 0.000814001 | 0.588674022 | 0.00855269 |
| **common_up** | FADS1 | 0.338237101 | 0.002670491 | 0.52980069 | 2.92E-05 |
| **Low_specific_down** | ALAD | -0.959737391 | 0.552298454 | -5.901407463 | 0.00292425 |
| **Low_specific_down** | PCDH17 | -1.717811727 | 0.214411549 | -5.820786977 | 0.005271018 |
| **Low_specific_down** | AC008443.7 | -1.177722 | 0.375064247 | -5.820786813 | 0.005271018 |
| **Low_specific_down** | PRKACB | -0.244435956 | 0.893790305 | -5.752483974 | 0.004884361 |
| **Low_specific_down** | RNU6-57P | -0.674530572 | 0.503327536 | -3.202854629 | 0.00760906 |
| **Low_specific_down** | FO393422.1 | -0.343931425 | 0.773022529 | -3.202637794 | 0.005538848 |
| **Low_specific_down** | PLEK2 | -1.349084974 | 0.11916588 | -2.786571441 | 0.008252449 |
| **Low_specific_down** | MESD | -0.41191508 | 0.666007724 | -2.662101547 | 0.004052744 |
| **Low_specific_down** | ASCC2 | -1.312738492 | 0.11047877 | -2.524366917 | 0.009090877 |
| **Low_specific_down** | ASS1P12 | -0.878090606 | 0.01256158 | -1.942535 | 0.000169769 |
| **Low_specific_down** | HSFX3 | -0.982125632 | 0.018028785 | -1.384627838 | 0.009560459 |
| **Low_specific_down** | DNAJC24 | -0.55466182 | 0.013241627 | -0.801027807 | 0.007147477 |
| **Low_specific_down** | AATBC | -0.578657393 | 0.011214178 | -0.692649569 | 0.002839926 |
| **Low_specific_down** | CHADL | -0.696004691 | 0.025504008 | -0.651123444 | 0.006148045 |
| **Low_specific_down** | KDM2B | -0.54633561 | 0.018035952 | -0.550719565 | 0.004290879 |
| **Low_specific_down** | CRBN | -0.356927215 | 0.024681169 | -0.485303724 | 0.006493048 |
| **Low_specific_up** | TRIM61 | 3.692100227 | 0.230135725 | 5.526937596 | 0.004567958 |
| **Low_specific_up** | LMX1B | 3.750793207 | 0.036035349 | 4.376486551 | 0.001363956 |
| **Low_specific_up** | IL21R | 2.269555868 | 0.224885775 | 3.95476227 | 0.007747624 |
| **Low_specific_up** | RPL23AP10 | 2.401266536 | 0.105280785 | 3.499348558 | 0.002048967 |
| **Low_specific_up** | AC018470.1 | 2.22862545 | 0.262488574 | 3.442177984 | 0.002463455 |
| **Low_specific_up** | TSC22D4 | 2.492361969 | 0.174458914 | 3.341081051 | 0.003636791 |
| **Low_specific_up** | Z82195.3 | 2.873394483 | 0.077718302 | 3.325843799 | 0.005623587 |
| **Low_specific_up** | MRPL23-AS1 | 2.524936863 | 0.041376052 | 3.317644396 | 0.001568587 |
| **Low_specific_up** | AC092910.1 | 1.887897076 | 0.270464951 | 3.170178332 | 0.00873122 |
| **Low_specific_up** | AC005086.2 | 1.835417348 | 0.146957128 | 3.081326144 | 0.003846541 |
| **Low_specific_up** | BBOX1 | 2.39062142 | 0.046441467 | 3.032730734 | 0.004927959 |
| **Low_specific_up** | AC236972.3 | 2.265833758 | 0.031573814 | 3.022385716 | 0.000514895 |
| **Low_specific_up** | AC008498.2 | 1.47132519 | 0.252754961 | 2.973780983 | 0.000581202 |
| **Low_specific_up** | DUPD1 | 0.893278331 | 0.741928861 | 2.772952323 | 0.009382607 |
| **Low_specific_up** | GPBP1L1 | 1.567526487 | 0.117395026 | 2.58072349 | 0.002119546 |
| **Low_specific_up** | AP000344.2 | 1.714397777 | 0.016013746 | 2.511407739 | 2.25E-05 |
| **Low_specific_up** | AC133552.4 | 1.968166654 | 0.041371154 | 2.453934358 | 0.001073294 |
| **Low_specific_up** | AL023755.1 | 1.471838255 | 0.05317794 | 2.441126227 | 0.003173975 |
| **Low_specific_up** | TATDN3 | 2.065437273 | 0.014015664 | 2.427691892 | 0.003285968 |
| **Low_specific_up** | AC037193.1 | 2.101477866 | 0.010851662 | 2.298556805 | 0.009145343 |
| **Low_specific_up** | TRPA1 | 1.450790577 | 0.091347309 | 2.292158131 | 0.005462069 |
| **Low_specific_up** | ALDH1L1 | 1.897682431 | 0.011884822 | 2.011795704 | 0.005487992 |
| **Low_specific_up** | PLA2G3 | 1.442443054 | 0.09759699 | 1.891937471 | 0.004504598 |
| **Low_specific_up** | FCF1P8 | 1.072076552 | 0.211584298 | 1.879222945 | 0.00873122 |
| **Low_specific_up** | MYH16 | 1.61113803 | 0.013272057 | 1.806094673 | 0.00649976 |
| **Low_specific_up** | NKILA | 1.480694642 | 0.014213605 | 1.734321325 | 0.007339072 |
| **Low_specific_up** | PLXDC1 | 1.837089792 | 0.022257152 | 1.710964597 | 0.009607903 |
| **Low_specific_up** | AC012557.1 | 1.345504049 | 0.096549524 | 1.686617811 | 0.00855269 |
| **Low_specific_up** | PDCL3P3 | 0.911011687 | 0.133442442 | 1.57863626 | 0.005046346 |
| **Low_specific_up** | GGA2 | 1.053358209 | 0.067217388 | 1.561268918 | 0.003949158 |
| **Low_specific_up** | KCNC1 | 0.672785877 | 0.298421528 | 1.534632573 | 0.002258446 |
| **Low_specific_up** | MOG | 1.035010945 | 0.03663395 | 1.531974551 | 0.000265298 |
| **Low_specific_up** | ENPP7P12 | 0.953417002 | 0.08866778 | 1.443864926 | 0.003436392 |

| Category | | High_vs_control | | Low_vs_control | |
|---|---|---|---|---|---|
| *Type* | *gene* | *FC(fold change)* | *FDR* | *FC(fold change)* | *FDR* |
| **Low_specific_up** | TNFRSF10A | 0.95675811 | 0.022414089 | 1.346733441 | 0.000102029 |
| **Low_specific_up** | AC010307.2 | 0.771948605 | 0.036355027 | 1.292014968 | 0.000182474 |
| **Low_specific_up** | AC008750.2 | 0.556173651 | 0.035717531 | 1.253098209 | 2.61E-08 |
| **Low_specific_up** | ZNF446 | 0.547741083 | 0.093266203 | 1.194848762 | 7.21E-09 |
| **Low_specific_up** | HLA-F | 0.76590147 | 0.138217356 | 1.181874261 | 0.003919329 |
| **Low_specific_up** | LINC02449 | 0.86980626 | 0.028280149 | 1.124529632 | 0.004164931 |
| **Low_specific_up** | PPIHP1 | 0.925193127 | 0.058619055 | 1.05385983 | 0.007866817 |
| **Low_specific_up** | GOLPH3L | 0.577809078 | 0.118627376 | 1.040452613 | 0.001203763 |
| **Low_specific_up** | CSNK1G2-AS1 | 0.653507468 | 0.054707273 | 0.990579024 | 0.002956624 |
| **Low_specific_up** | SPAG4 | 0.489567572 | 0.208103663 | 0.975854222 | 0.00566697 |
| **Low_specific_up** | CDC14B | 0.638168158 | 0.011866944 | 0.966745558 | 0.000354858 |
| **Low_specific_up** | AC129492.7 | 0.56358646 | 0.056265473 | 0.958911912 | 0.000182474 |
| **Low_specific_up** | TMEM64 | 0.513413085 | 0.193251501 | 0.958266624 | 0.003460589 |
| **Low_specific_up** | SCD | 0.554133546 | 0.1783838 | 0.942940107 | 0.002084198 |
| **Low_specific_up** | AUXG01000058.1 | 0.672537775 | 0.011225189 | 0.849218254 | 0.001547983 |
| **Low_specific_up** | MTHFR | 0.544938028 | 0.015459715 | 0.814302247 | 0.004227943 |
| **Low_specific_up** | AC083806.2 | 0.470940029 | 0.168274257 | 0.803964886 | 0.00500558 |
| **Low_specific_up** | CLIP3 | 0.403526708 | 0.134996071 | 0.803330334 | 0.000227859 |
| **Low_specific_up** | MCHR1 | 0.620876179 | 0.049430754 | 0.772642641 | 0.009738903 |
| **Low_specific_up** | AKAP9 | 0.696598952 | 0.014129382 | 0.770871746 | 0.007675711 |
| **Low_specific_up** | ISG20L2 | 0.472611277 | 0.116676418 | 0.758594693 | 0.004531444 |
| **Low_specific_up** | ZNF337 | 0.736511577 | 0.012287162 | 0.754227667 | 0.006162288 |
| **Low_specific_up** | HPN-AS1 | 0.566399748 | 0.024138794 | 0.728178664 | 6.58E-05 |
| **Low_specific_up** | TRIM39 | 0.397167059 | 0.086408542 | 0.639738393 | 0.005929586 |
| **Low_specific_up** | Z98884.1 | 0.400066509 | 0.014990024 | 0.618148175 | 0.000510628 |
| **Low_specific_up** | AC125611.4 | 0.387603016 | 0.052927469 | 0.59063033 | 0.003442381 |
| **Low_specific_up** | AL590989.1 | 0.315908088 | 0.287205812 | 0.569984966 | 0.007675711 |
| **Low_specific_up** | LINC01529 | 0.400219993 | 0.037099835 | 0.564627555 | 0.008173185 |
| **Low_specific_up** | AC096536.2 | 0.293358754 | 0.061589115 | 0.536040898 | 0.002991281 |
| **Low_specific_up** | AC009065.7 | 0.292344442 | 0.142097571 | 0.530610537 | 0.00887632 |
| **Low_specific_up** | AC096536.3 | 0.280647517 | 0.011944788 | 0.430206354 | 0.004760893 |

'High_specific_up/down' represents high dose-specific up/down-expression and 'Low_specific_up/down' represents low dose-specific up/down-expression. 'common_up/down' represents up/down-expression both high and low dose.

## Table S2. Biological function of DEG groups

| Group | GOTerm | Term PValue | Group PValue | GO Groups | % Associated Genes | Nr. Genes | Associated Genes Found |
|---|---|---|---|---|---|---|---|
| **High_specific_Up** | Retinol metabolism | 0.11 | 0.11 | Group00 | 4.48 | 3.00 | [ADH6, DGAT1, LRAT] |
| **High_specific_Up** | RUNX1 interacts with co-factors whose precise effect on RUNX1 targets is not known | 0.03 | 0.03 | Group01 | 7.89 | 3.00 | [CSNK2A2, PBRM1, SMARCA2] |
| **High_specific_Up** | FOXO-mediated transcription | 0.10 | 0.10 | Group02 | 4.62 | 3.00 | [ATXN3, BBC3, NFYC] |
| **High_specific_Up** | Response of EIF2AK1 (HRI) to heme deficiency | 0.00 | 0.00 | Group03 | 20.00 | 3.00 | [CEBPB, CHAC1, TRIB3] |
| **High_specific_Up** | Spinocerebellar ataxia | 0.01 | 0.01 | Group04 | 6.12 | 6.00 | [ATG14, ATXN2, ATXN3, RORA, VLDLR, WIPI1] |
| **High_specific_Up** | Bacterial invasion of epithelial cells | 0.13 | 0.13 | Group05 | 4.11 | 3.00 | [ARHGEF26, ARPC4, BCAR1] |
| **High_specific_Up** | Cellular hexose transport | 0.01 | 0.01 | Group06 | 14.29 | 3.00 | [FGF21, MFSD4B, SLC2A14] |
| **High_specific_Up** | Unfolded Protein Response (UPR) | 0.08 | 0.08 | Group07 | 4.26 | 4.00 | [CEBPB, CXXC1, NFYC, WIPI1] |
| **High_specific_Up** | Transcriptional regulation of white adipocyte differentiation | 0.06 | 0.06 | Group08 | 4.76 | 4.00 | [CDK4, CEBPB, FAM120B, LEP] |
| **High_specific_Up** | Ca2+ pathway | 0.09 | 0.09 | Group09 | 4.84 | 3.00 | [GNB3, PDE6B, TCF7L1] |
| **High_specific_Up** | Ion homeostasis | 0.07 | 0.07 | Group10 | 5.36 | 3.00 | [DMPK, FXYD7, RYR3] |
| **High_specific_Up** | SLC transporter disorders | 0.09 | 0.09 | Group11 | 4.08 | 4.00 | [NUP62, SLC20A2, SLC34A3, SLC35A3] |
| **High_specific_Up** | p53 signaling pathway | 0.01 | 0.03 | Group12 | 6.94 | 5.00 | [BBC3, BID, CDK4, SESN2, TP53I3] |
| **High_specific_Up** | TP53 Regulates Transcription of Cell Death Genes | 0.04 | 0.03 | Group12 | 6.82 | 3.00 | [BBC3, BID, TP53I3] |
| **High_specific_Up** | Metabolism of xenobiotics by cytochrome P450 | 0.04 | 0.06 | Group13 | 5.19 | 4.00 | [ADH6, ALDH3B1, CYP2D7, EPHX1] |
| **High_specific_Up** | Drug metabolism | 0.13 | 0.06 | Group13 | 4.17 | 3.00 | [ADH6, ALDH3B1, CYP2D7] |
| **High_specific_Up** | mTOR signaling pathway | 0.02 | 0.03 | Group14 | 4.52 | 7.00 | [AKT1S1, DDIT4, MIOS, NPRL2, SESN2, SLC7A5, WNT2B] |
| **High_specific_Up** | Amino acids regulate mTORC1 | 0.07 | 0.03 | Group14 | 5.45 | 3.00 | [MIOS, NPRL2, SESN2] |
| **High_specific_Up** | PPARA activates gene expression | 0.00 | 0.00 | Group15 | 5.98 | 7.00 | [FAM120B, G0S2, HMGCR, NFYC, RORA, SLC27A1, TRIB3] |
| **High_specific_Up** | Regulation of lipid metabolism by PPARalpha | 0.00 | 0.00 | Group15 | 5.88 | 7.00 | [FAM120B, G0S2, HMGCR, NFYC, RORA, SLC27A1, TRIB3] |
| **High_specific_Up** | Human cytomegalovirus infection | 0.02 | 0.02 | Group16 | 4.00 | 9.00 | [BCAR1, BID, CDK4, CXCR2, GNAI3, GNB3, MYC, NFATC4, VEGFA] |
| **High_specific_Up** | Kaposi sarcoma-associated herpesvirus infection | 0.02 | 0.02 | Group16 | 4.23 | 8.00 | [ATG14, BID, CDK4, GNB3, MYC, NFATC4, VEGFA, ZFP36] |
| **High_specific_Up** | Bladder cancer | 0.03 | 0.02 | Group16 | 7.32 | 3.00 | [CDK4, MYC, VEGFA] |
| **High_specific_Up** | Chaperonin-mediated protein folding | 0.08 | 0.06 | Group17 | 4.21 | 4.00 | [CSNK2A2, FBXW4, GNAI3, GNB3] |

| | | | | | | | |
|---|---|---|---|---|---|---|---|
| **High_specific_Up** | G alpha (z) signalling events | 0.05 | 0.06 | Group17 | 6.25 | 3.00 | [GNAI3, GNB3, RGS17] |
| **High_specific_Up** | Cooperation of PDCL (PhLP1) and TRiC/CCT in G-protein beta folding | 0.03 | 0.06 | Group17 | 7.14 | 3.00 | [CSNK2A2, GNAI3, GNB3] |
| **High_specific_Up** | Amino sugar and nucleotide sugar metabolism | 0.05 | 0.03 | Group18 | 6.25 | 3.00 | [AMDHD2, GNPNAT1, MPI] |
| **High_specific_Up** | Biosynthesis of the N-glycan precursor (dolichol lipid-linked oligosaccharide, LLO) and transfer to a nascent protein | 0.01 | 0.03 | Group18 | 6.41 | 5.00 | [ALG13, ALG5, AMDHD2, GNPNAT1, MPI] |
| **High_specific_Up** | Synthesis of substrates in N-glycan biosythesis | 0.02 | 0.03 | Group18 | 6.35 | 4.00 | [ALG5, AMDHD2, GNPNAT1, MPI] |
| **High_specific_Up** | Rap1 signaling pathway | 0.01 | 0.02 | Group19 | 4.29 | 9.00 | [BCAR1, F2RL3, FGF21, GNAI3, ITGA2B, PARD6G, RAPGEF3, RASGRP2, VEGFA] |
| **High_specific_Up** | Integrin signaling | 0.00 | 0.02 | Group19 | 14.81 | 4.00 | [BCAR1, ITGA2B, RAPGEF3, RASGRP2] |
| **High_specific_Up** | Platelet activation, signaling and aggregation | 0.00 | 0.02 | Group19 | 4.56 | 12.00 | [BCAR1, DGKQ, F2RL3, GNAI3, GNB3, ITGA2B, MPL, NHLRC2, RAPGEF3, RASGRP2, TIMP3, VEGFA] |
| **High_specific_Up** | Platelet Aggregation (Plug Formation) | 0.00 | 0.02 | Group19 | 12.82 | 5.00 | [BCAR1, ITGA2B, MPL, RAPGEF3, RASGRP2] |
| **High_specific_Down** | Regulation of TP53 Activity | 0.00 | 0.00 | Group0 | 6.25 | 10.00 | [BRD7, CHEK2, PPP1R13B, RAD17, RBBP7, RFC5, STK11, TAF10, TAF5, TAF6] |
| **High_specific_Down** | Regulation of TP53 Activity through Phosphorylation | 0.00 | 0.00 | Group1 | 7.61 | 7.00 | [CHEK2, RAD17, RFC5, STK11, TAF10, TAF5, TAF6] |
| **Common_Up** | Protein-protein interactions at synapses | 0.00 | 0.00 | Group0 | 4.65 | 4.00 | [DBNL, LIN7B, LRFN4, SLITRK6] |
| **Common_Up** | O-linked glycosylation of mucins | 0.01 | 0.01 | Group1 | 4.76 | 3.00 | [GCNT1, MUC16, MUC3A] |
| **Common_Up** | Regulation of cholesterol biosynthesis by SREBP (SREBF) | 0.00 | 0.00 | Group2 | 10.91 | 6.00 | [DHCR7, FASN, FDFT1, INSIG1, MVD, MVK] |
| **Common_Up** | Cholesterol biosynthesis | 0.00 | 0.00 | Group2 | 16.00 | 4.00 | [DHCR7, FDFT1, MVD, MVK] |
| **Common_Up** | Activation of gene expression by SREBF (SREBP) | 0.00 | 0.00 | Group2 | 11.90 | 5.00 | [DHCR7, FASN, FDFT1, MVD, MVK] |
| **Common_Down** | Bile secretion | 0.00 | 0.00 | Group0 | 4.17 | 3.00 | [ADCY3, SLC51A, SLC51B] |
| **Common_Down** | RNA polymerase II transcribes snRNA genes | 0.00 | 0.00 | Group1 | 4.05 | 3.00 | [INTS11, INTS8, POLR2J] |

There was no significant GO (gene ontology) terms for low specific DEGs
