# Supplementary material for: An Ethanol Extract of Coptidis rhizoma Induces Apoptotic Cell Death in Induced Pluripotent Stem Cells and Suppresses Teratoma Formation
Source: Nutrients. 2023 May 18;15(10):2364. doi: 10.3390/nu15102364 (PMC10221726; doi:10.3390/nu15102364)
Supplement: Supplementary file 1 [file nutrients-15-02364-s001.zip › Table 1, 2, 3.pdf]

Table 1. UHPLC condition for analysis

Parameter		Analytical conditions	
Column temperature		30°C	
UV		DAD at 350 nm	
Spectra range		190 to 500 nm	
Injection volume		2 μL	
Flow rate		0.5 mL/min	
Column		Phenomenex Luna C_{18} (4.6 x 250 mm, 5 μm)	
Sample reconstruction		2 mg/mL in MeOH	
**Mobile Phase**	**Time** (min)	**A (%)** (0.1% formic acid in water)	**B (%)** (Acetonitrile)
	0	72	28
	35	90	10
	50	Washing and equilibrium	

Table 2. Quantitation parameters

Constituent	Linear range (μg/mL)	Regression equation [a)]		Correlation coefficient, r^2	LOD (μg/mL)	LOQ (μg/mL)
		Slope	Intercept			
Coptisine	2 – 200	24405	8.067	0.9999	0.541	1.638
Palmatine	5 – 200	15315	5.335	0.9999	0.890	2.696
Berberine	1 - 200	35455	15.435	0.9999	0.263	0.797

[a)] y = ax + b, y and x indicate the peak area and the concentration of sample (μg/mL), respectively.

Table 3. The amount of each constituent in ECR

Compound	Content (mg/g)
Coptisine	31.62 ± 0.09
Palmatine	48.56 ± 0.23
Berberine	84.01 ± 0.11
